# Phylogenetic Signals of Salinity and Season in Bacterial Community Composition Across the Salinity Gradient of the Baltic Sea

**DOI:** 10.3389/fmicb.2016.01883

**Published:** 2016-11-24

**Authors:** Daniel P. R. Herlemann, Daniel Lundin, Anders F. Andersson, Matthias Labrenz, Klaus Jürgens

**Affiliations:** ^1^Leibniz Institute for Baltic Sea ResearchWarnemünde, Germany; ^2^Centre for Ecology and Evolution in Microbial Model Systems, Linnaeus UniversityKalmar, Sweden; ^3^Science for Life Laboratory, Division of Gene Technology, School of Biotechnology, KTH Royal Institute of TechnologyStockholm, Sweden

**Keywords:** ecological coherence, brackish microbiology, estuarine ecology, *Verrucomicrobia*, SAR11, microbial ecology

## Abstract

Understanding the key processes that control bacterial community composition has enabled predictions of bacterial distribution and function within ecosystems. In this study, we used the Baltic Sea as a model system to quantify the phylogenetic signal of salinity and season with respect to bacterioplankton community composition. The abundances of 16S rRNA gene amplicon sequencing reads were analyzed from samples obtained from similar geographic locations in July and February along a brackish to marine salinity gradient in the Baltic Sea. While there was no distinct pattern of bacterial richness at different salinities, the number of bacterial phylotypes in winter was significantly higher than in summer. Bacterial community composition in brackish vs. marine conditions, and in July vs. February was significantly different. Non-metric multidimensional scaling showed that bacterial community composition was primarily separated according to salinity and secondly according to seasonal differences at all taxonomic ranks tested. Similarly, quantitative phylogenetic clustering implicated a phylogenetic signal for both salinity and seasonality. Our results suggest that global patterns of bacterial community composition with respect to salinity and season are the result of phylogenetically clustered ecological preferences with stronger imprints from salinity.

## Introduction

The spatial and temporal variability of aquatic microbial communities is generally attributed to a combination of environmental factors that influence the composition of the biotic community. For animals and plants, their phylogenetic classification is of ecological relevance, since closely related taxa tend to occupy similar ecological niches. However, because microorganisms evolve quickly, their phylogenetic affiliation is a rather unreliable ecological indicator (e.g., Vasi et al., 1994). Yet, data from genome analyses and ecological studies suggest that phylogenetically clustered microorganisms exhibit a considerable degree of ecological similarity (Logares et al., 2009; Philippot et al., 2010). These so-called “phylogenetic signals” of the bacterial communities have been associated with different habitats (Andersson et al., 2009; Stegen et al., 2012; Morrissey and Franklin, 2015; Salazar et al., 2015), growth response (Goldfarb et al., 2011), and different ecological strategies (Evans and Wallenstein, 2014), indicating that phylogenetic clusters share strategies that distinguish them from other groups at broad taxonomic levels (Philippot et al., 2010). However, few studies exist comparing the impact of different environmental variables on the phylogenetic signal of bacterial communities. Salinity, temperature, and dissolved oxygen are among the most important environmental factors determining aquatic microbial community composition (Crump et al., 2004; Fuhrman et al., 2008; Herlemann et al., 2011). A global-scale meta-analysis of samples from different habitats suggested that salinity is the major determinant of bacterial communities (Lozupone and Knight, 2007), and strong seasonal shifts in the bacterial communities of marine as well as brackish environments have been demonstrated (Andersson et al., 2009; Gilbert et al., 2009; Lindh et al., 2015). In addition, long-term studies suggest predictable seasonal patterns of bacterial community dynamics (Fuhrman et al., 2008; Gilbert et al., 2012; Ladau et al., 2013).

The strength of different, simultaneously acting environmental factors on bacterial community composition is difficult to assess and related efforts have been limited. The reasons include the inconsistency of publicly available microbial gene sequences, incomparable experimental methods, co-varying environmental factors, and the inaccessibility of consistent environmental information. Moreover, the processes governing the variation in community composition may greatly differ between habitats. For example, a study in the Columbia River showed the dominance of salinity effects over seasonal changes (Fortunato et al., 2013), whereas a study in the Chesapeake Bay found that seasonal factors were stronger than spatial ones in determining bacterial community composition (Kan et al., 2007). However, in many estuaries the relative impact of different factors is difficult to determine, given the complex and highly dynamic hydrological conditions characteristic of these sites (Fortunato and Crump, 2015). The Baltic Sea, in contrast, is a more tractable system, with a stable salinity gradient that facilitates comparisons of the impact of salinity vs. other environmental factors. Moreover, the central Baltic Sea has a water residence time of 30 years (Reissmann et al., 2009), which has allowed the establishment of mesohaline (“brackish”) microbial communities (Herlemann et al., 2011; Dupont et al., 2014; Hugerth et al., 2015; Hu et al., 2016). The environmental conditions in the Baltic Sea show the typical seasonal changes of high-latitude ecosystems, including strong shifts in temperature, solar radiation, phytoplankton blooms, nutrient levels, and organic matter composition. Consequently, bacterial community composition in the Baltic Sea is strongly influenced by seasonal dynamics (Pinhassi and Hagström, 2000; Riemann et al., 2008; Andersson et al., 2009; Lindh et al., 2015).

In a previous study, we described the major role played by salinity in determining bacterial community composition in the Baltic Sea, with distinct bacterial communities living under oligohaline, mesohaline, and marine conditions during the summer (Herlemann et al., 2011). We also identified typical mesohaline bacterial members in the central Baltic Sea, including the verrucomicrobial taxon “*Candidatus* Spartobacterium balticum” (Herlemann et al., 2013; Bergen et al., 2014) and the “SAR11-IIIa” clade (Herlemann et al., 2014). Here, we extend these earlier analyses by analyzing a transect dataset sampled in winter and comparing bacterial community composition in winter and summer along the salinity gradient of the Baltic Sea. Our aim was to detect potential differences in the impacts of salinity and seasonality on bacterial phylogenetic composition. We show that both factors influence the bacterial community composition with stronger imprints from salinity.

## Materials and Methods

### Sampling

**FIGURE 1:**
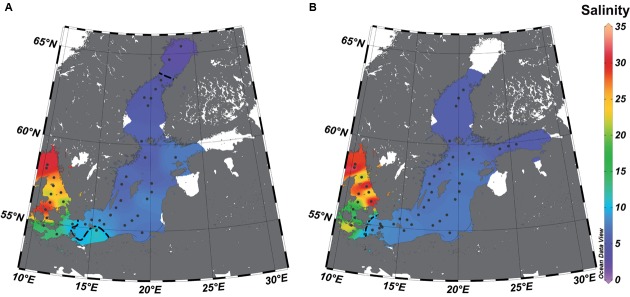
**Study area and sampling stations (dots) in the surface water along the salinity gradient of the Baltic Sea.** The salinities were extrapolated based on the values (see Supplementary Table S1) of the stations from July **(A)** and February **(B)** by piece-wise linear regression using Ocean Data View 4.7. The dotted line represents the estimated transitions between the salinity areas (salinity 10 = transition marine–mesohaline; salinity 4 = transition mesohaline–oligohaline), and the white areas those with unreliable extrapolations.

Water samples were obtained during a research cruise on the *R/V* Alkor in February 2009 (**Figure 1**, Supplementary Figure S1). Conductivity, temperature, pressure, and the dissolved oxygen content of the water samples were recorded using a conductivity/temperature/depth sensor (CTD) SeaBird 911 connected to a rosette of 24 10-L bottles (Supplementary Table S1). No samples were taken below a salinity of 4 in February since ice cover prevented sampling. Concentrations of inorganic nutrients and oxygen were analyzed according to standard methods (Grasshoff et al., 1983). Water samples (1 L) for DNA analysis were filtered (0.2-μm pore-size white polycarbonate filters), and DNA was extracted according to Weinbauer et al. (2002). Samples with an oxygen concentration <2 mg/L were excluded from the analysis because low-oxygen water is known to harbor distinct bacterial communities, which were not the objective of this study. The study includes also samples from the July transect study in 2008 (Herlemann et al., 2011), that have been prepared similar to those in February.

### PCR and 454 Sequencing

Filtered water samples were PCR-amplified as described in Herlemann et al. (2011). In brief, 30 ng of the extracted DNA was amplified using the primers Bakt_341F and Bakt_805R, complemented with 454 adapters and sample-specific 5-bp barcodes. The PCR conditions consisted of a denaturing step of 95° for 5 min, 25 cycles of 40 s at 95°C, 40 s at 53°C, and 60 s at 72°C, and a final extension step of 5 min at 72°C. The resulting amplicons were purified using Agencourt^©^ AMPure^®^ XP (Becker Coulter), quantified with the Picogreen assay (Molecular Probes), mixed in equimolar amounts, and sequenced from the reverse primer direction by MWG Eurofins using Roche/454 GS FLX Titanium technology. The raw sequences from the February cruise were deposited in the ENA Sequence Read Archive under accession number PRJEB14590 (July data are deposited under ENA accession number PRJEB1245).

### Sequence Processing

Raw sequences from a July transect study in 2008 (Herlemann et al., 2011) and the sequences obtained in this study were combined and denoised using AmpliconNoise (Quince et al., 2011). After truncation of the sequences to 400 bp, the primer sequences were removed. Processed sequences were clustered into phylotypes using the Usearch (Edgar, 2010) program based on a minimum of 99% sequence identity and the implemented chimera checking. A 99% similarity radius was chosen because seed-based clustering (based on radii) resembles 98% complete linkage clustering (based on diameters). The seed sequence, i.e., the most abundant sequence of each phylotype, was aligned to a local Silva database [SSURef_108_NR_99 downloaded in April 2012 (Quast et al., 2012)] using SINA (Pruesse et al., 2012). The operational taxonomic unit (OTU) was assigned the taxonomy of the best hit if the identity over ≥380 bp was ≥95%. Reads assigned to chloroplasts as well as singletons (reads present only once in the total dataset) were removed. Samples with <1000 reads were then excluded.

### Statistical Analysis

For richness and Shannon estimations Explicet (Robertson et al., 2013) was used, which performs a rarefaction-based analysis through bootstrapping. For all stations, bootstrap resampling was conducted at the size of the smallest library (1001 reads) at the rarefaction point, to compare OTUs between libraries at equal sampling efforts. A non-metric multidimensional scaling (NMDS) plot was based on sum-normalized OTU abundances and calculated using Bray—Curtis dissimilarities implemented in the PAST software package version 3.08 (Hammer et al., 2001). The environmental variables salinity, depth, season (July = 1; February = 2), and temperature were added as *post hoc* vectors to the NMDS graph representing the correlation coefficients between the environmental variables and the NMDS scores. An analysis of similarities (ANOSIM) was used to test statistically significant differences in bacterial community composition, using the Bray and Curtis dissimilarity index. A linear discriminant analysis (LDA) effect size (LEfSe version 1.0) analysis (Segata et al., 2011), with a minimum LDA = 2 and the “all against all” strategy, was used to identify differential abundance patterns among the different salinities and seasons (Supplementary Tables S2 and S3). The sequences of the abundant (>1%) OTUs identified by LEfSe were aligned using the SINA web aligner (Pruesse et al., 2012) and related full-length sequences were added. The latter were used to calculate a maximum-likelihood (ML) tree as implemented in ARB (Ludwig et al., 2004). Short sequences were added using the ARB parsimony tool, without changing the global tree topology.

### Phylogenetic Signal

To investigate if phylogenetic distance is related to niche differences of OTUs, i.e., if there exists a phylogenetic signal, we used the method of Stegen et al. (2012) in scripts for R Statistical Software (R Core Team, 2015), as described in Salazar et al. (2015). Only surface water samples of the congruent dataset (stations having one surface sample per station) were used in this analysis. For each OTU, two niche values were calculated, one for salinity and one for season. For salinity, the niche value for OTU *i* was calculated as (a_i1_ ×s_1_ + a_i2_ × s_2_ + … + a_iN_ × s_N_)/(a_i1_ + a_i2_ + … + a_iN_), where *a_ij_* is the relative abundance of OTU *i* in sample *j*, *s_j_* the salinity of sample *j*, and *N* the total number of samples. For season, the niche value was calculated accordingly, but here *s_j_* represented season of sample *j* and was set to 1 for the summer samples and 2 for the winter samples. Using this procedure each OTU received an abundance-weighted value for salinity and season. Subsequently, “between-OTU niche difference” was calculated for salinity and season for each pair of OTUs, as the absolute difference between their niche values. The between-OTU phylogenetic distance of all sequences from the dataset were determined by aligning the most abundant sequences of each OTU using the SINA web aligner (Pruesse et al., 2012) and importing them into ARB (Ludwig et al., 2004). Sequences with good alignment quality (pintail score >80) were merged with the Silva_123_NR tree containing high-quality full-length sequences using the quick add tool provided in ARB. After the short sequences were placed among the long sequences, the long sequences of the Silva_123_NR sequences were removed from the phylogenetic tree and the resulting tree was used to determine the phylogenetic distance between each OTU (“between-OTU phylogenetic distance”). Very long phylogenetic branches were excluded from this analysis (Supplementary Table S4; Supplementary Figure S3) since they were potential chimeric sequences or had exceptional high evolutionary rates. Finally, the OTU pairs where binned in phylogenetic distance intervals of 0.01 (arbitrary units). Within each bin the mean of the “between-OTU niche difference” values were calculated, and these were regressed against the bins’ phylogenetic distances.

### Maps

The maps of the vertical and horizontal salinity gradient were plotted using Ocean Data View (Schlitzer, 2011). Data from the July and February cruises were interpolated using a piecewise linear regression, which takes into account all data from the station as implemented in Ocean Data View.

## Results

### Bacterial Richness and Changes in Community Composition

We investigated 120 samples from February (winter samples) and 106 samples from July (summer samples), both covering a salinity range of 2.6–35.2, a temperature range of 0–19.4°C, and a depth range of 1–300 m (**Figure 1**; Supplementary Table S1; Supplementary Figure S1). After quality filtering, 326,089 sequencing reads (1,001–3,128 reads per sample) were clustered in 11,424 OTUs. In addition to its separation into summer and winter samples, the samples were classified (**Table 1**) into surface water samples (0–10 m; *n* = 72; 100,982 reads), mesopelagic (11–300 m) samples (*n* = 154; 218,123 reads), marine (salinity > 10) samples (*n* = 103; 140,916 reads), mesohaline (salinity 10–4) samples (*n* = 117; 169,476 reads), and oligohaline (salinity < 4) samples (*n* = 6; 8,733 reads).

**Table 1:** Number of sequences and operational taxonomic units (OTUs) in the different salinity and seasonal zones examined in this study.

Category		Samples	Reads	Number of OTUs^a^
Summer (July)	Total	106	147,661	1,238
Marine	Surface	13	17,038	709
	Congruent^b^	4	5,252	423
	Mesopelagic	41	55,621	1,348
Mesohaline	Surface	19	27,502	735
	Congruent	13	19,122	515
	Mesopelagic	27	37,573	1,045
Oligohaline	Surface	6	8,733	601

Winter (February)	Total	120	172,024	2,349
Marine	Surface	8	10,325	1,400
	Congruent^b^	4	5,034	788
	Mesopelagic	41	56,606	1,986
Mesohaline	Surface	26	36,600	1,412
	Congruent^b^	13	17,189	911
	Mesopelagic	45	65,774	1,608

*^a^Samples were combined according to the categories and rarefied to the smallest number of reads (8733 reads all data; 5034 congruent stations) to calculate richness.*

*^b^Congruent stations are surface water stations that were sampled at similar positions and depths in July and February.*

**FIGURE 2:**
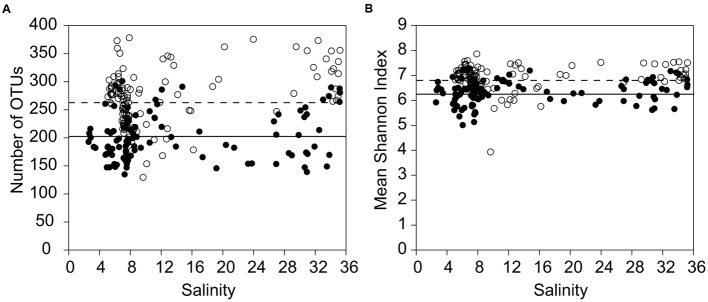
**Bacterial alpha-diversity at different salinities and seasons. (A)** Comparison of the bacterial richness between all samples in July and all samples in February, sorted by salinity. **(B)** Shannon index of the bacterial communities, including all July samples and all February samples, sorted by salinity. For all stations, bootstrap re-sampling was conducted at the size of the smallest library (1001 reads) at the rarefaction point, to compare the observed operational taxonomic units (OTUs) between libraries at equal sampling effort (filled circles = July, open circles = February). The dotted line gives the average for February; the bold line the average for July.

Bacterial richness and Shannon diversity, represented by the number of rarefied OTUs per sample, were significantly (Kruskal–Wallis, *p* < 0.05) higher in the February samples (130–378 OTUs per sample; 1,238 OTUs all samples combined) than in the July samples (135–301 OTUs per sample; 2,349 all samples combined; **Table 1**; **Figure 2**). By contrast, there was no clear pattern along the salinity gradient for either bacterial richness or the Shannon diversity (**Figure 2**). Especially in February, there were strong fluctuations in the number of OTUs, even within stations representing the same salinity region (e.g., salinity 8.2; 177 OTUs vs. salinity 7.8; 378 OTUs).

**FIGURE 3:**
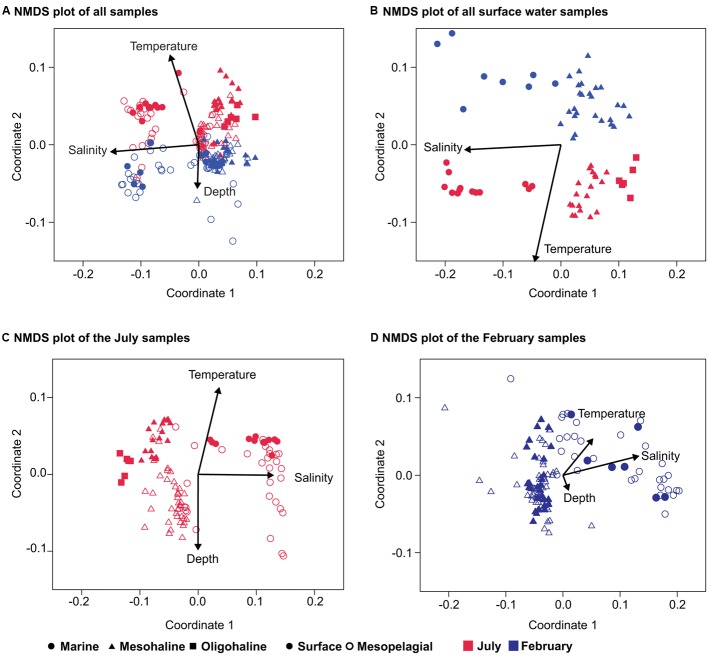
**Non-metric multidimensional scaling (NMDS) of the bacterial community composition in the Baltic Sea. (A)** NMDS of all samples (stress 0.24); **(B)** only surface-water samples (stress 0.13); **(C)** NMDS plot of the July samples (stress 0.17); and **(D)** NMDS plot of the bacterial community composition in the February samples (stress 0.09). The environmental variables salinity, depth, and temperature were added as *post hoc* vectors to the NMDS graph representing the correlation coefficients between the environmental variables and the NMDS scores. Bold symbols are surface samples (0–10 m), and open symbols the mesopelagic (11–300 m). Samples from the February cruise are indicated in blue and those from the July cruise in red.

An analysis of bacterial community composition by NMDS plots indicated a separation of the bacterial communities, with salinity inversely correlating with the first coordinate (Pearson correlation *r* = -0.93) and differences between the July and February samples (season) correlating with the second coordinate (Pearson correlation *r* = 0.70; **Figure 3A**). The second coordinate was also inversely correlated with depth (Pearson correlation *r* = -0.31). The analysis of the surface water samples supported the results obtained with the complete dataset and confirmed a clear separation between the July and February samples along the second coordinate (**Figure 3B**). Analyzing the July and February samples separately revealed a clear separation between surface and mesopelagic samples (stratification) along the second NMDS coordinate for the July samples (**Figure 3C**), but not for the February samples (**Figure 3D**). This is consistent with the bigger difference in temperature between these water layers in July (average 15°C, ± 2°C and average 7°C, ± 4°C for surface and mesopelagic samples, respectively) than in February (2 ± 1°C and 4 ± 2°C, respectively). For both the February and July samples the first NMDS coordinate correlated with salinity.

### Bacterial Phylotypes Characteristic of Salinity and Season

**FIGURE 4:**
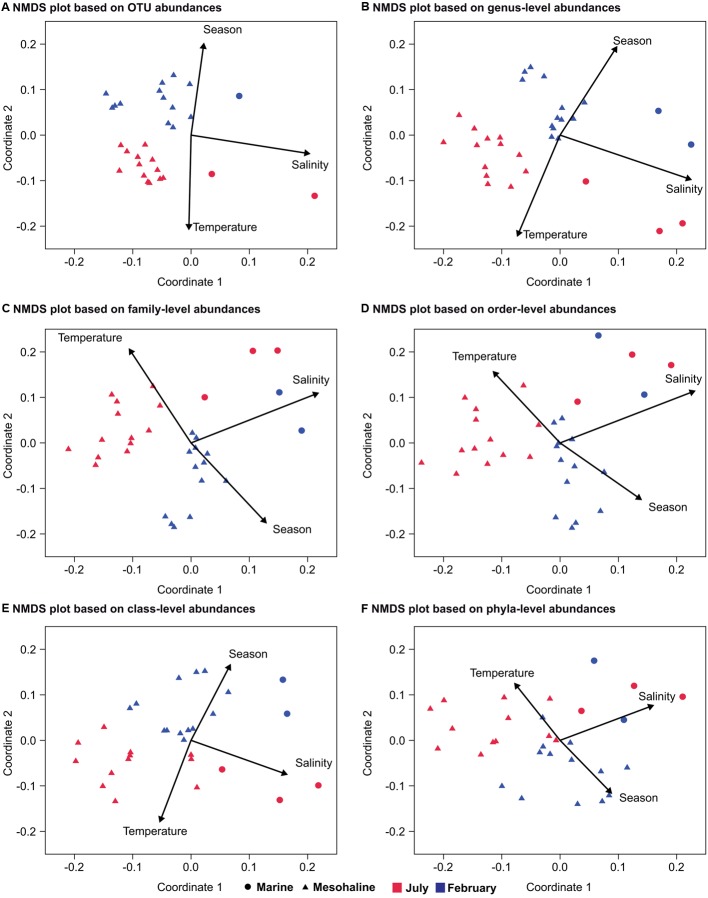
**Non-metric multidimensional scaling of bacterial communities from a congruent dataset (surface water and marine and mesohaline only) at different phylogenetic levels based on SILVA rank taxonomy.** NMDS at: **(A)** the OTU level (stress: 0.13); **(B)** the genus level (stress: 0.13); **(C)** the family level (stress: 0.14); **(D)** the order level (stress: 0.17); **(E)** the class level (stress: 0.15); and **(F)** the phylum level (stress: 0.13). The environmental variables salinity, season, and temperature were added as *post hoc* vectors to the NMDS graph representing the correlation coefficients between the environmental variables and the NMDS scores. Samples from the February cruise are indicated in blue and those from the July cruise in red.

To exclude the effects of increasing depth, which is linked to the stratification of temperature, light, and nutrients, on bacterial community composition, the following analysis included only the communities in the surface water samples. To make the summer and winter dataset consistent we only included stations sampled both in February and July (Supplementary Figure S1; **Table 1**). This resulted in 34 surface water samples and excluded the oligohaline stations that could not be sampled in February, due to ice cover. ANOSIM-based comparisons of the bacterial communities at the different salinities and during the two seasons revealed a larger *R*-value for salinity (ANOSIM *p* < 0.01, *R* = 0.84) than for season (ANOSIM *p* < 0.01, *R* = 0.41; **Table 2**). When the analysis was performed from the OTU to the phylum level, the *R*-values were lower but the salinity values were still higher than the seasonal values (**Table 2**). Consistent with these results, NMDS plots of the bacterial community composition at different taxonomic ranks showed a separation based on salinity along the first coordinate and separation of the July and February samples along the second coordinate (**Figure 4**). The separation based on the first and second coordinates of the NMDS plots was strongest at the OTU level (**Figure 4A**). At the genus level, the separation between the July and February samples and between salinity levels was still obvious, but the degree of correlation of the vectors with the first and second coordinates decreased. A decrease in the correlation with the first and second coordinates of the NMDS continued from the family level to the phylum level, together with a decrease in the strict separation between the July and February samples (**Figures 4C,D,F**). The separation at the class level of both the February and July samples and the marine and mesohaline samples was relatively clear (**Figure 4E**).

**Table 2:** Analysis of similarity (ANOSIM) of the bacterial community composition using a dataset with congruent stations at different salinities in February and July.

	Salinity		Season	
	*P*-value	*R*-value	*P*-value	*R*-value
OTU	<0.01	0.84	<0.01	0.41
Genus	<0.01	0.82	<0.01	0.33
Family	<0.01	0.75	<0.01	0.33
Order	<0.01	0.74	<0.01	0.32
Class	<0.01	0.74	<0.01	0.27
Phylum	<0.01	0.60	<0.01	0.28

**FIGURE 5:**
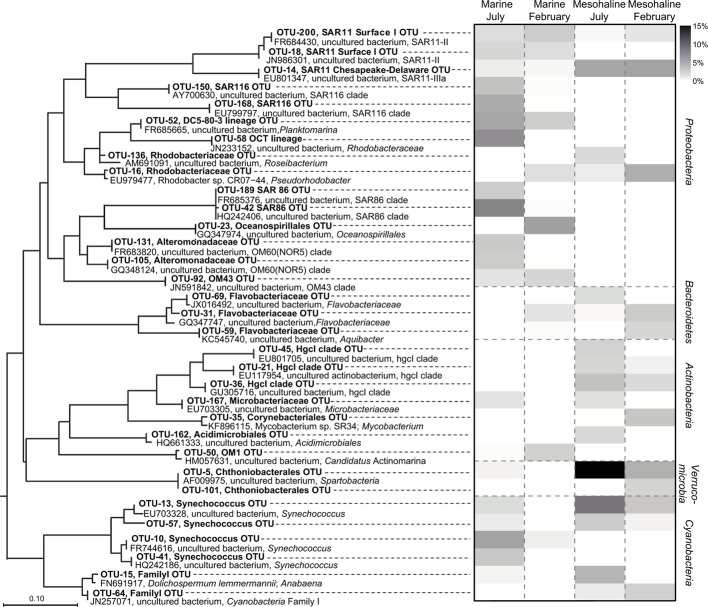
**Phylogenetic tree and heat map of high-abundant indicator operational taxonomic units (OTUs).** The heat map shows the relative abundances of the abundant (>1%) OTUs identified by a least discriminant analysis (LDA) effect size (LEfSe) analysis of the congruent dataset (bold). The OTUs are arranged based on a maximum-likelihood (ML) tree of full-length sequences chosen based on their close phylogenetic affiliation with the indicator OTU sequences. The short indicator OTU sequences from our study were added without changing the tree topology, after calculation of the ML-tree. The scale bar is only approximate because the procedure distorts branch length. Original sequence definitions were replaced by a consistent nomenclature, including Genbank accession number, name, and next defined taxonomic level.

Representative bacterial OTUs, classes, and phyla for season and salinity were identified by applying the LEfSe to the congruent dataset. This resulted in the identification of 280 OTUs for the marine samples and 51 OTUs for the mesohaline samples with significantly higher relative abundances at the respective salinity (Supplementary Table S2). Among the abundant OTUs (>1% relative abundance; Supplementary Table S3; **Figure 5**), different representatives of the cyanobacterial genus *Synechococcus* were typical for either the marine or the mesohaline samples (OTU-41, OTU-10 vs. OTU-13, OTU-57, OTU-64). Representatives of the SAR11 clade (“Pelagibacterales”) were present among the marine and mesohaline samples. While the mesohaline samples were dominated by a SAR11-IIIa OTU (OTU-14), in the marine samples two other OTUs from the SAR11 group (SAR11-II) were dominant (OTU-18, OTU-200). Other typical alphaproteobacterial OTUs in the marine samples were SAR116, *Roseobacter* OCT lineage, *Planktomarina*, the gammaproteobacteria SAR86, “unclassified Oceanospirillales,” “unclassified Alteromonadales,” NOR5/OM60 (*Alteromonadaceae*), a representative of OM43 (*Betaproteobacteria*) and “*Candidatus* Actinomarina” (*Actinobacteria*). In the mesohaline environment, after *Synechococcus*, an OTU from *Spartobacteria* was the most abundant, with other representative OTUs including those assigned to *Flavobacteriaceae (Bacteroidetes)*, *Rhodobacteriaceae* (*Alphaproteobacteria*), and the actinobacterial family *Corynebacteriales* as well as two OTUs from the hgcI-clade [also referred to as the “acI-clade” (Warnecke et al., 2005)].

The dominant OTUs in February (153 OTUs) and July (136 OTUs), were also determined (Supplementary Tables S2, S3). The most abundant OTUs in the July samples belonged to *Spartobacteria* and *Synechococcus* as well as to the hgcI-clade, “unclassified Microbacteriaceae,” “unclassified Acidimicrobiales”, and “unclassified Flavobacteriaceae” (**Figure 5**). The February samples comprised significantly higher abundances of OTUs belonging to SAR11-IIIa (*Alphaproteobacteria*), *Rhodobacter*, two *Flavobacteria*, *Corynebacteria*, and an “unclassified Spartobacterium”. The sequence identity of the February and July spartobacterial OTUs (OTU-5 and OTU-101, respectively) differed by 1% (based on 379 bp) and both had low-relative abundances (**Figure 5**).

**FIGURE 6:**
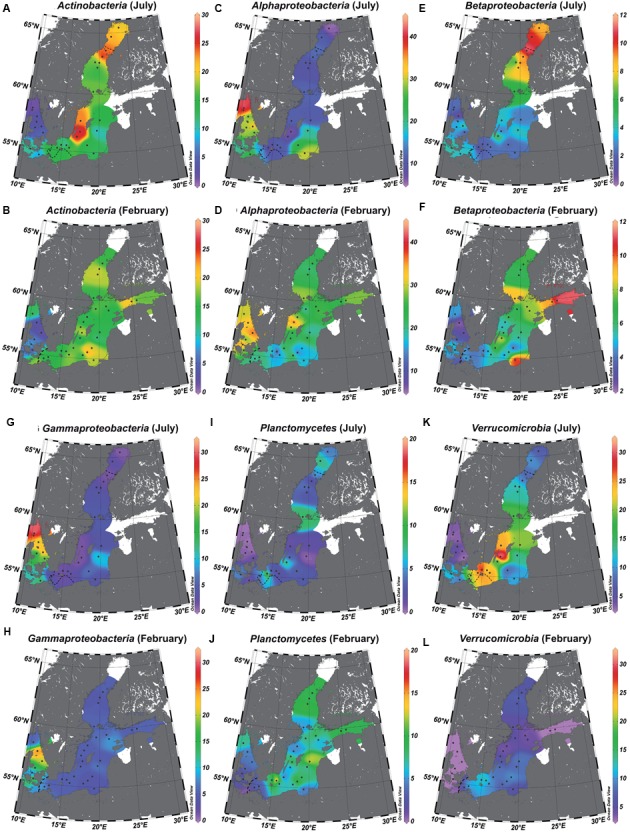
**Relative abundance of bacterial phyla/classes in the salinity gradient of the Baltic Sea.** The data were extrapolated to those of the surface water using piece-wise linear regression. *Actinobacteria* (**A** = July and **B** = February), *Alphaproteobacteria* (**C** = July and **D** = February), *Betaproteobacteria* (**E** = July and **F** = February), *Gammaproteobacteria* (**G** = July and **H** = February), *Planctomycetes* (**I** = July and **J** = February), *Verrucomicrobia* (**K** = July and **L** = February). The dotted line represents the estimated transitions between the salinity areas (salinity 10 = transition marine–mesohaline; salinity 4 = transition mesohaline–oligohaline), and the white areas those with unreliable extrapolations. Interpolation and maps were generated using Ocean Data View 4.7.

We extrapolated the surface water distribution of the characteristic, abundant bacterial phyla/classes—identified by LEfSe as having a significantly higher abundance at one of the salinity levels—to the salinity gradient of the Baltic Sea (**Figure 6**). In accordance with the OTU level analysis, the phyla/classes with significantly higher abundances in the mesohaline samples were *Actinobacteria* (**Figures 6A,B**), *Betaproteobacteria* (**Figures 6E,F**), *Planctomycetes* (**Figures 6I,J**), and *Verrucomicrobia* (**Figures 6K,L**). In the marine samples, they were *Alphaproteobacteria* (**Figures 6C,D**) and *Gammaproteobacteria* (**Figures 6G,H**). In **Figure 6**, *Cyanobacteria* were excluded, since cyanobacterial mats of filamentous *Cyanobacteria* may not have been sufficiently sampled by the applied sampling method.

**FIGURE 7:**
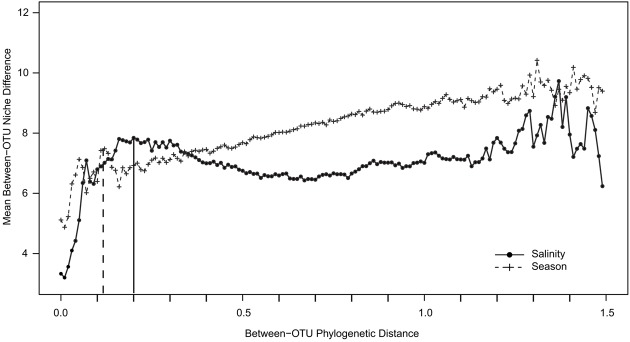
**Between-OTU niche difference as a function of between-OTU phylogenetic distance.** The data points represent means of OTU niche differences within phylogenetic distance bins. Plus signs and filled dots represent niche differences with respect to salinity and season, respectively. The dotted vertical line gives the maximum peak for season; the bold vertical line gives the maximum peak for salinity.

To determine whether closely related OTUs share ecological niches with respect to season and salinity (“phylogenetic signal”), an abundance-weighted niche value was defined for each OTU for salinity and season using the congruent dataset (see section “MATERIALS AND METHODS”). The niche value differences between pairs of OTUs were plotted against their phylogenetic distance. A steep positive relationship was observed between niche value difference and phylogenetic distance for both salinity and season at low phylogenetic distances (**Figure 7**). The slope of the curve declined for season around phylogenetic distance 0.1 while it declined later for salinity, around 0.2.

## Discussion

The bacterial communities identified along the salinity gradient of the Baltic Sea are consistent with salinity-driven global patterns of bacterial community composition. Marine waters are dominated by *Alpha*- and *Gammaproteobacteria*, and limnetic areas by *Actinobacteria* and *Betaproteobacteria* (Kirchman et al., 2005; Logares et al., 2009; Lefort and Gasol, 2013). The typical abundance shift of *Cyanobacteria* as well as changes in bacterial community composition and richness between July and February were also observed in this study (Fuhrman et al., 2008; Fortunato and Crump, 2015), with the dataset showing that both salinity-related and seasonal differences caused phylogenetically clustered shifts in bacterial community composition. However, the impact of salinity was stronger than the differences between July and February.

The richness of the mesohaline bacterial community in the central Baltic Sea was comparable with that of the marine and oligohaline bacterial communities in February (**Figure 2**). This is in accordance with the results of our previous study in which only the July data were considered (Herlemann et al., 2011). The absence of a decline in bacterial diversity in the brackish waters of the Baltic Sea contradicts Remane’s criteria of diversity in the Baltic Sea, deduced from benthic invertebrates, which included a species minimum in the brackish zone (Remane, 1934; Zettler et al., 2014). Deviations from the mesohaline species-minimum proposed for macrozoobenthos have also been noted in other estuaries for bacterial communities (Crump et al., 2004; Hewson and Fuhrman, 2004), zooplankton (Laprise and Dodson, 1994), phytoplankton (Muylaert et al., 2009; Schubert et al., 2011), ciliates (Dolan and Gallegos, 2001), and protists (Telesh et al., 2010). Telesh et al. (2013) suggested that the life strategies of unicellular planktonic organisms differ substantially from those of large multicellular bottom-dwelling organisms, resulting in deviations from the species-minimum concept, which is supported by the results from our study of bacterioplankton. However, we detected strong fluctuations in bacterial richness within very narrow salinity ranges, especially in February (**Figure 2**). This suggests that factors other than salinity influenced bacterial richness. Whereas the February samples were characterized by a significantly higher bacterial richness and Shannon diversity (**Figure 2**), prokaryotic cell numbers were significantly higher in July (Supplementary Table S1). We assume that the increased phytoplankton-derived production of labile dissolved organic matter in July was responsible for the increase in the cell numbers of heterotrophic prokaryotes and for the proliferation of several adapted taxa (Bunse et al., 2016). This and the fact that our sequencing efforts were designed to cover the dominant OTUs could explain the lower richness values in July. Accordingly, only a few dominant OTUs (e.g., OTU-5 and OTU-13) were identified as indicator OTUs in July whereas the distribution of indicator OTUs in February was relatively even (**Figure 5**; Supplementary Table S3). Bacterial diversity in February may have been further increased due to the mixing of different water masses containing distinct bacterial communities (**Figure 3D**). Our analysis showed that bacterial communities in the Baltic Sea are separated at the thermocline in July (**Figure 3C**). In February, the water masses again mix with the bacterial communities such that OTUs from the former mesopelagial are found within those of the surface water.

### Impact of Salinity and Seasonality on Bacterial Community Composition

The NMDS analyses of the surface water bacterial communities (**Figure 4**) indicate that both salinity and temperature had a significant impact on bacterial community composition. By contrast, factors such as oxygen concentration, inorganic phosphate, SiO_2_, and NO_3_^-^ showed no clear correlation with either the primary or the secondary coordinate of the NMDS analysis (Supplementary Figure S2). Like Stegen et al. (2012), we used a regression of “between-OTU niche value” difference (in this study, salinity and season) vs. “between-OTU phylogenetic distance” to investigate the relationship between the ecological niche and phylogenetic distance. The result revealed a steep positive relationship between them and supports therefore the presence of a phylogenetic signal. For salinity, the steep positive relationship between phylogenetic distance and niche difference continued until a phylogenetic distance of 0.2, while for season the slope declined earlier (∼0.1; **Figure 7**). An impact on broader phylogenetic levels for salinity was also supported by the changes of the correlation coefficients in the ANOSIM analysis (**Table 2**). A strong decrease in the R-value for season was observed between the OTU and genus levels (0.41–0.33) while these levels gave almost similar *R*-values for salinity (0.84–0.82). For salinity, the largest drop in *R*-value was instead between the genus and family levels (0.82–0.75) and between the class and phylum levels (0.74–0.60).

The strong phylogenetic signal linked to salinity is in line with a previous study demonstrating a phylogenetic signal of wetland soil bacteria based on salinity, albeit using a different approach (Morrissey and Franklin, 2015). The mechanisms causing changes in bacterial community composition at different salinities are currently unclear. A metagenomic study in the Baltic Sea was able to link salinity to differences in the key metabolic capabilities of bacteria, including differences in the relative abundance of genes associated with respiration, glycolysis, quinone biosynthesis, and osmolyte transport (Dupont et al., 2014). Based on the short generation times of many bacteria together with their rapid evolution and remarkable trophic versatility, environmental boundaries can be crossed more frequently than is the case for plants or animals. Therefore, in a connected system like the Baltic Sea there should be more salinity-generalists. However, with the exception of *Synechococcus*, the abundant bacteria found in marine and mesohaline waters (e.g., *Planktomarina*, “Unclassified Spartobacteria”) are phylogenetically unrelated, which suggests a deeply rooted divergent evolution for the communities at different salinity levels. However, other biotic factors, such as water clarity (Yannarell and Triplett, 2005), phytoplankton (Pinhassi et al., 2004), grazing (Jürgens and Matz, 2002), and viral lysis (Suttle, 1994), also shape bacterial community composition. Since these factors also change along the salinity gradient of the Baltic Sea (Riemann and Middelboe, 2002; Hu et al., 2016), the observed bacterial community composition patterns may be a result of factors that co-correlate with salinity.

Although the mesohaline samples contained *Synechococcus* (**Figure 5**, OTU-13; OTU-57) at high abundance, related *Synechococcus* OTUs (OTU-10 and OTU-41) were also found in the marine samples. These observations support the ubiquitous presence of closely related *Synechococcus* in different salinity environments in the Baltic Sea. However, the phylogenetic separation of *Synechococcus* based on 16S rRNA genes has also been shown to be weak (e.g., Haverkamp et al., 2009). In contrast to S*ynechococcus*, the SAR11 representatives identified in the marine (OTU-200 and OTU-18) and mesohaline (OTU-14) samples were phylogenetically distinct (**Figure 5**). In accordance with our previous study in the Baltic Sea, based on fluorescence *in situ* hybridization (Herlemann et al., 2014), the SAR11-IIIa lineage detected in this study was highly abundant under mesohaline conditions, especially in February. The SAR11-IIIa lineage found in brackish zones was replaced by the marine SAR11-II lineage in marine waters of the Baltic Sea. In contrast to investigations of the bacterial communities along the shoreline of the Gulf of Gdansk (Piwosz et al., 2013), we found no representative of the freshwater SAR11-IIIb clade (formerly “LD-12”) in the oligohaline samples of the Baltic Sea. However, the oligohaline areas of the Baltic Sea could not be sampled in February, and SAR11-IIIb may have been absent in our sampling campaign in July since SAR11-IIIb are poor competitors during phytoplankton blooms (Heinrich et al., 2013). The brackish and marine bacterial communities differed both in summer and in winter (**Figure 4**), which is in line with the results of a previous metagenome study (Dupont et al., 2014; Hugerth et al., 2015). In our analysis, *Verrucomicrobia*, and specifically those OTUs assigned to *Spartobacteria*, were particularly abundant in the brackish zone in July and February (**Figure 5**). Spartobacterial OTUs are known to co-occur with phytoplankton blooms (Herlemann et al., 2013; Lindh et al., 2013; Bergen et al., 2014) which are highly abundant in the brackish part of the Baltic Sea (Wasmund et al., 2011).

The differences between the July and February indicator taxa supported the NMDS and ANOSIM results suggesting a difference in bacterial community composition between seasons. The differences in the bacterial community composition between July and February indicate that these communities are not functionally redundant but are adapted phylogenetic groups, consistent with our detection of a phylogenetic signal for season (**Figure 7**). However, because the impact of season occurred at a finer phylogenetic distance than that of salinity, we propose that they act on different phylogenetic levels. Nonetheless, our investigation was limited to the annual amplitude of two contrasting seasons (July and February) and did not analyze the detailed seasonal dynamics of specific populations within years (Fuhrman et al., 2008; Andersson et al., 2009; Lindh et al., 2015).

In conclusion, our study showed significant differences in bacterial richness between seasons. Salinity was a stronger determinant of bacterial community composition than season. The impact of salinity and seasonality were also present on different phylogenetic levels, where seasonality acted at a finer phylogenetic level than salinity. Overall our results support the use of broad-level phylogenetic clusters as ecological indicators especially for salinity, since it allows predicting the distribution of bacterial taxa in salinity gradients. Moreover, phylogenetic information can be used to estimate the impact of perturbations on bacterial distribution patterns and abundances in a changing environment.

## Author Contributions

DH, AA, ML, and KJ conceived and designed the study. DH performed the experiments, and DH, DL, AA, and KJ analyzed the data. DH, DL, and AA contributed analysis tools. DH, DL, AA, ML, and KJ wrote the paper.

## Conflict of Interest Statement

The authors declare that the research was conducted in the absence of any commercial or financial relationships that could be construed as a potential conflict of interest.
