# Supplementary material for: Phylogenetic Signals of Salinity and Season in Bacterial Community Composition Across the Salinity Gradient of the Baltic Sea
Source: Front Microbiol. 2016 Nov 24;7:1883. doi: 10.3389/fmicb.2016.01883 (PMC5121245; doi:10.3389/fmicb.2016.01883)
Supplement: Supplementary file 1 [file Data_Sheet_1.PDF]

Supplementary Information

Phylogenetic signal of salinity and season on bacterial community compositions in the salinity gradient of the Baltic Sea

Daniel PR Herlemann, Daniel Lundin, Anders F Andersson, Matthias Labrenz, Klaus Jürgens

Supplementary Figures

Figure S1. Section plots of salinity and temperature in summer and winter

Figure S2. NMDS plots including all measured parameters

Figure S3. Phylogenetic tree of the sequences used for the phylogenetic signal

Supplementary Tables

Table S1. Stations and measured environmental parameters

Table S2. LDA scores of the LefSe Analysis

Table S3. Abundant indicator OTUs and their abundances

Table S4. Long branches excluded from the analysis of the phylogenetic signal

(A)

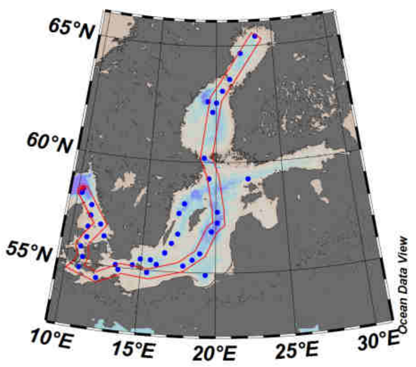

(B)

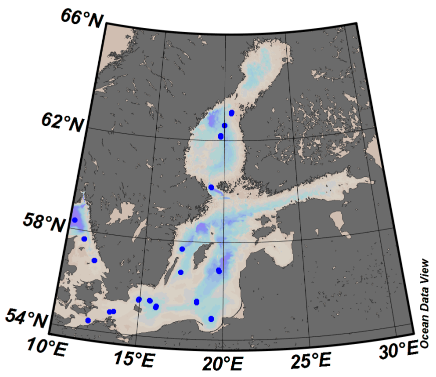

(C) temperature summer

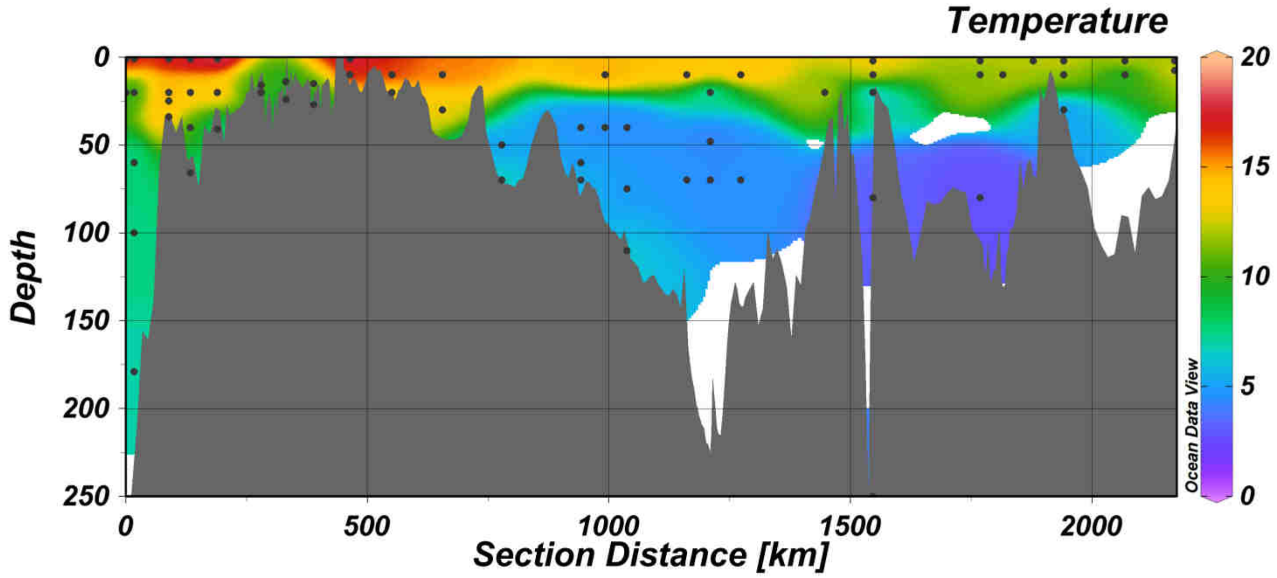

(D) temperature winter

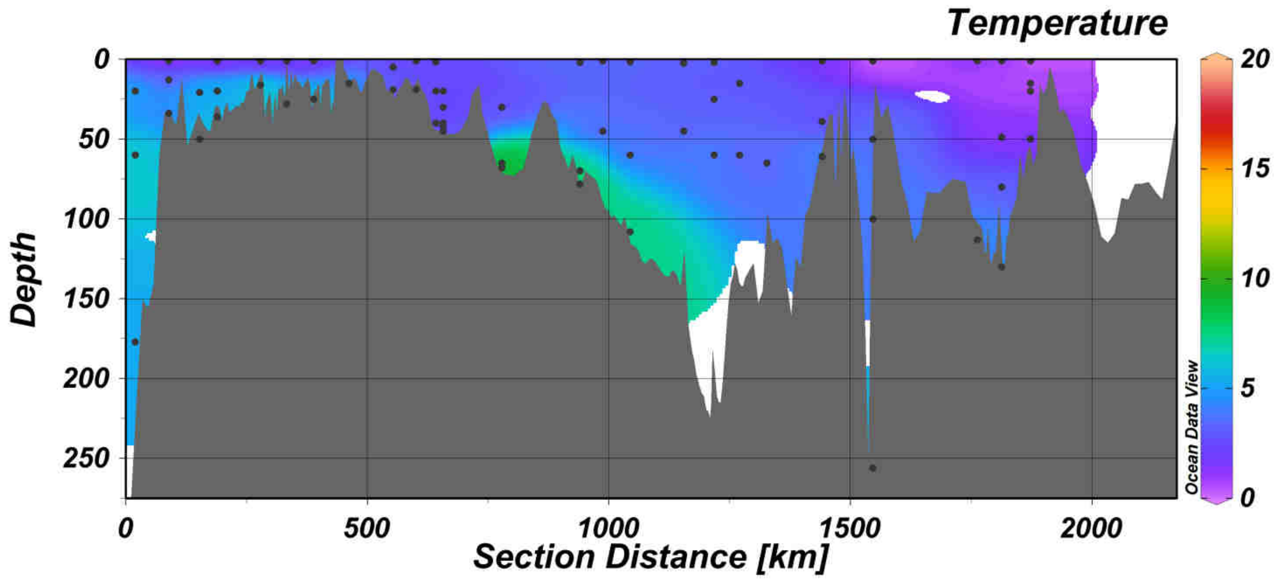

(E) salinity summer

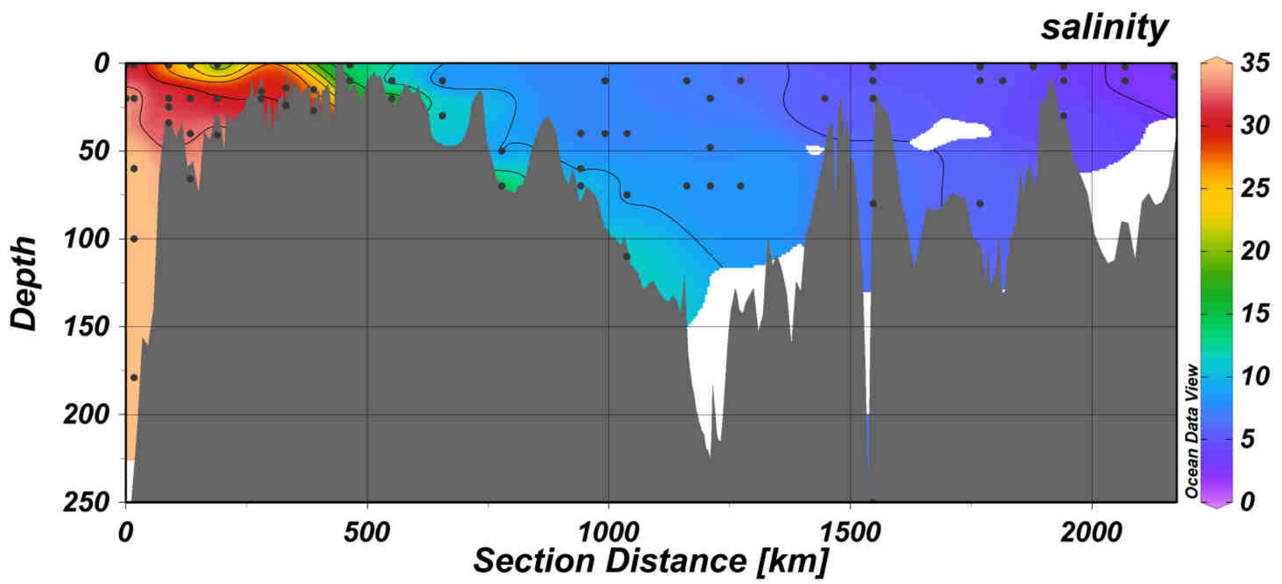

(F) salinity winter

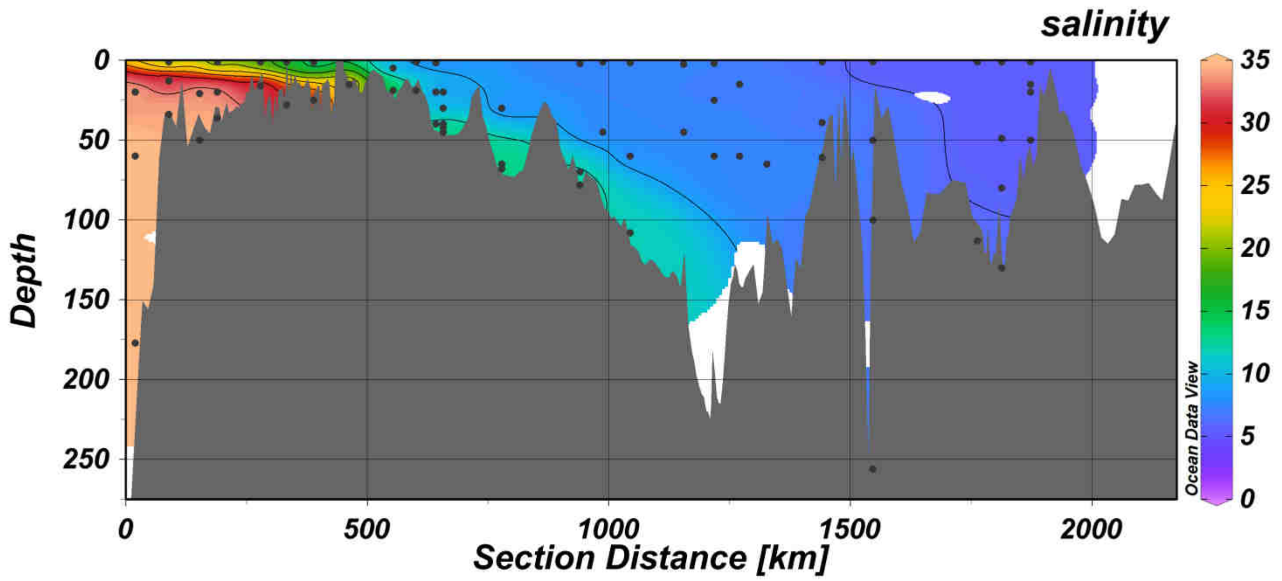

Figure S1.(A) Surface plot of the stations and the route used for section plots C-D. (B) Congruent stations used in the analysis. (C) extrapolated temperature in summer; (D) extrapolated temperature in winter; (E) extrapolated salinity in summer, (F) extrapolated salinity in winter. White areas mark missing data.

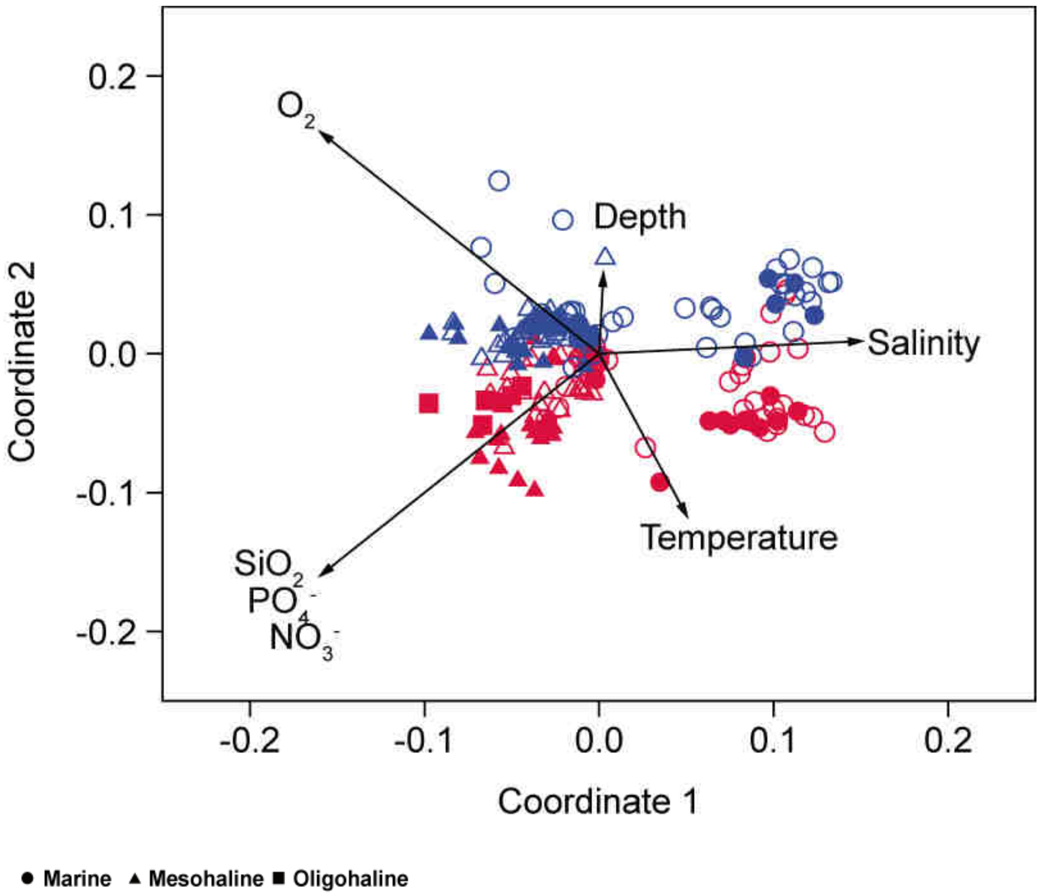

Figure S2. Non metric multidimensional scaling (NMDS) of the bacterial community composition covering all samples in the salinity gradient of the Baltic Sea in winter (blue) and summer (red) (stress 0.24). The environmental variables were added as vectors posthoc to the NMDS graph representing the correlation coefficients between the environmental variables and the NMDS scores. (full symbol = surface, open symbol = hypolimnion)

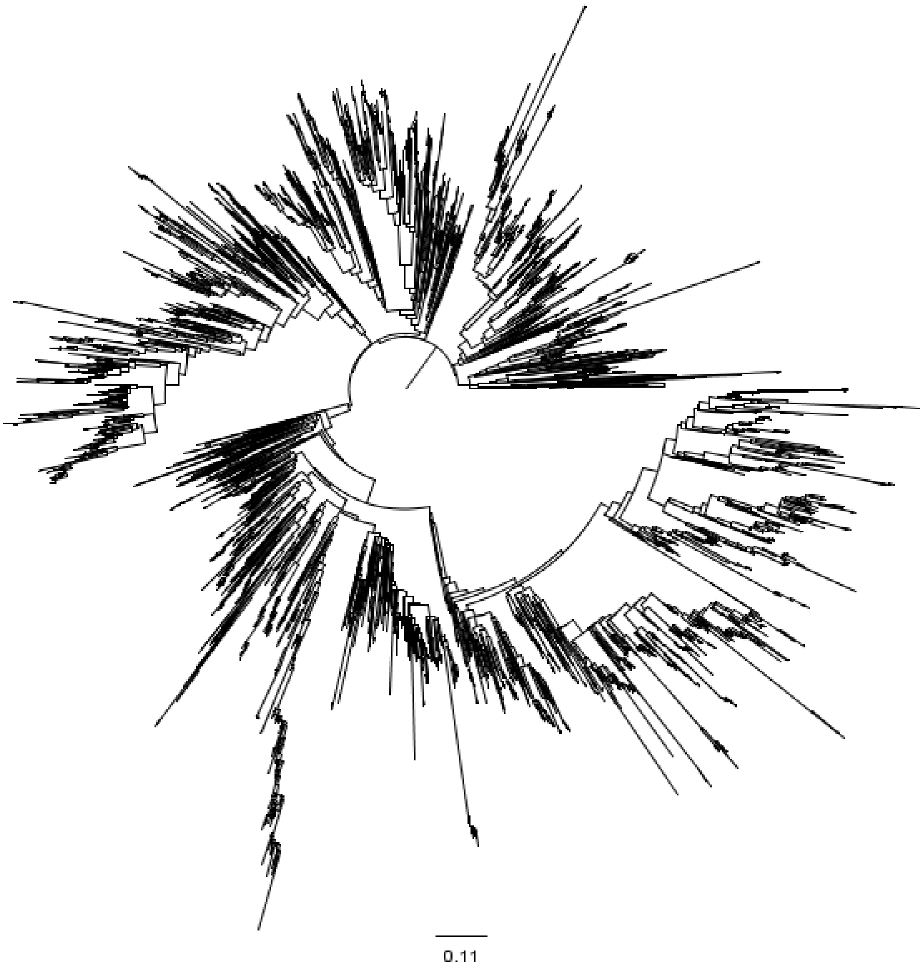

Figure S3. Phylogenetic tree of the OTUs included in the analysis for the phylogenetic signal. Long branches were excluded from the analysis.

Tabe S1. Coordinates, depth, salinity, temperature, nutrient concentration and cell counts (BC) in the samples. Given is also the saline and seasonal category. Bold samples are samples used in the complementary dataset. ("-" not determined)

Sample	Latitude	Longitude	Season	Salinity category	salinity	Depth (m)	Temperature (°C)	O_2 (ml/l)	PO_4^- (µM)	SiO_2 (µM)	NO_2^- (µM)	NO_3^- (µM)	NH_4^+ (µM)	BC (cells/ml)
**S1**	**54.4**	**12.1**	**Summer**	**Marine**	**10.5**	**10**	**16.6**	**6.6**	**0.1**	**9.0**	**0.0**	**0.1**	**-**	**1.08E+06**
S10	55.3	16.0	Summer	Marine	9.4	60	5.1	6.6	-	-	-	-	-	5.27E+06
S101	58.2	17.7	Summer	Mesohaline	6.5	20	7.2	7.1	0.2	7.6	0.0	0.0	-	2.81E+06
S104	57.7	17.4	Summer	Mesohaline	6.2	17	6.8	7.3	-	-	-	-	-	2.72E+06
S106	57.7	17.4	Summer	Mesohaline	7.5	55	3.8	7.0	1.0	15.6	0.3	4.2	-	9.19E+05
**S107**	**57.7**	**17.4**	**Summer**	**Mesohaline**	**5.9**	**10**	**14.6**	**7.8**	**0.1**	**6.3**	**0.0**	**0.0**	**0.3**	**5.03E+06**
S109	57.7	17.4	Summer	Mesohaline	7.2	40	3.9	7.7	0.6	11.4	0.3	1.8	0.9	6.51E+05
**S111**	**56.8**	**17.4**	**Summer**	**Mesohaline**	**5.6**	**10**	**15.0**	**7.8**	**0.0**	**4.9**	**0.0**	**0.0**	**-**	**5.11E+06**
S112	56.8	17.4	Summer	Mesohaline	6.0	20	12.4	6.4	0.1	6.2	0.0	0.0	-	3.73E+06
S114	56.8	17.4	Summer	Mesohaline	7.7	60	3.8	5.9	1.2	22.3	0.2	6.8	-	1.31E+06
S117	56.3	17.0	Summer	Mesohaline	6.5	40	3.3	7.2	0.9	15.5	0.2	6.6	-	5.13E+05
S119	56.3	17.0	Summer	Mesohaline	5.0	10	15.6	8.0	0.0	3.9	0.0	0.0	-	8.04E+06
S12	55.2	14.4	Summer	Mesohaline	7.7	10	15.1	6.8	0.2	11.8	0.0	0.0	-	1.72E+06
S121	56.3	17.0	Summer	Mesohaline	7.7	60	3.9	5.1	1.5	25.7	0.1	8.6	-	2.33E+06
S125	55.9	16.5	Summer	Mesohaline	7.4	60	3.7	5.5	1.4	23.8	0.1	9.0	0.3	1.73E+06
S13	55.2	14.4	Summer	Marine	8.0	30	8.0	6.5	0.5	14.3	0.0	0.0	-	8.56E+05
S132	59.3	22.4	Summer	Mesohaline	7.5	56	3.9	5.8	1.2	21.3	0.2	5.4	-	1.08E+06
S14	55.2	14.4	Summer	Marine	12.1	40	7.0	3.6	1.0	28.3	0.3	2.1	-	9.04E+05
S140	59.3	19.5	Summer	Mesohaline	5.7	20	12.2	7.1	0.0	8.6	0.0	0.0	-	6.63E+06
**S15**	**55.5**	**14.9**	**Summer**	**Mesohaline**	**7.6**	**10**	**15.0**	**6.9**	**0.2**	**13.1**	**0.0**	**0.0**	**-**	**1.71E+06**
S16	55.5	14.9	Summer	Marine	8.4	40	6.7	6.6	0.5	13.7	0.1	0.0	-	7.94E+05
S17	55.5	14.9	Summer	Marine	10.5	50	6.1	5.6	-	-	-	-	-	8.87E+05
S173	56.8	17.4	Summer	Mesohaline	7.6	60	4.7	6.6	0.9	19.1	0.4	2.0	-	1.76E+06
S182	55.9	16.5	Summer	Marine	8.7	49	5.2	6.2	0.8	16.0	0.2	1.6	-	2.27E+06
S184	55.1	13.3	Summer	Marine	12.1	30	13.7	5.2	0.5	12.0	0.3	0.2	-	2.66E+06
S186	54.8	10.9	Summer	Marine	13.3	1	17.9	6.1	0.1	8.4	0.0	0.1	-	1.00E+07
S187	54.8	10.9	Summer	Marine	13.9	10	17.7	6.1	0.1	5.8	0.0	0.1	-	6.65E+06
**S19**	**55.5**	**15.6**	**Summer**	**Mesohaline**	**7.5**	**10**	**15.3**	**7.0**	**0.3**	**15.4**	**0.0**	**0.0**	**-**	**2.46E+06**
S190	55.3	11.1	Summer	Marine	27.2	15	10.7	5.3	0.4	8.9	0.1	0.7	-	4.07E+06
S191	55.3	11.1	Summer	Marine	30.7	27	8.2	6.1	-	-	-	-	-	4.88E+06
S193	55.8	10.8	Summer	Marine	29.9	14	9.5	6.2	0.0	2.0	0.0	0.0	-	3.63E+06
S194	55.8	10.8	Summer	Marine	30.6	24	8.8	5.2	0.4	12.6	0.1	3.3	-	3.81E+06
S196	56.2	11.1	Summer	Marine	29.0	16	11.2	5.8	-	-	-	-	-	3.47E+06
S197	56.2	11.1	Summer	Marine	30.8	20	9.5	5.0	0.3	16.2	0.2	3.1	-	5.54E+06
S199	56.4	12.3	Summer	Marine	17.4	1	19.4	5.9	0.0	0.5	0.0	0.0	0.0	3.39E+06
S2	54.4	12.1	Summer	Marine	17.0	20	13.3	5.6	0.4	11.2	0.0	0.3	-	1.07E+06
S20	55.5	15.6	Summer	Mesohaline	7.7	40	5.2	4.7	0.6	16.0	0.0	0.1	-	5.39E+05
S200	56.4	12.3	Summer	Marine	23.3	10	15.9	6.1	0.0	1.6	0.0	0.0	-	2.92E+06
S201	56.4	12.3	Summer	Marine	28.6	20	14.6	5.6	0.0	2.0	0.0	0.1	-	3.01E+06
S203	56.7	11.1	Summer	Marine	20.4	1	17.2	6.2	0.0	1.4	0.0	0.0	-	3.63E+06
S204	56.7	11.1	Summer	Marine	21.5	5.5	16.7	6.2	-	-	-	-	-	3.91E+06
S205	56.7	11.1	Summer	Marine	26.9	10	14.5	5.5	-	-	-	-	-	3.85E+06
S206	56.7	11.1	Summer	Marine	26.6	13	14.6	5.5	0.1	4.2	0.0	0.1	-	4.18E+06
**S207**	**56.9**	**11.9**	**Summer**	**Marine**	**19.2**	**1**	**18.2**	**5.9**	**0.0**	**0.3**	**0.0**	**0.0**	**-**	**3.52E+06**
S209	56.9	11.9	Summer	Marine	30.1	20	14.2	5.5	0.1	2.3	0.1	0.5	-	2.55E+06

Sample	Latitude	Longitude	Season	Salinity category	salinity	Depth (m)	Temperature (°C)	O_2 (ml/l)	PO_4 (µM)	SiO_2 (µM)	NO_2 (µM)	NO_3 (µM)	NH_4 (µM)	BC (cells/ml)
S21	55.5	15.6	Summer	Marine	11.2	60	5.7	4.8	1.2	24.6	0.1	5.2	-	5.09E+05
S210	56.9	11.9	Summer	Marine	33.8	41	7.4	4.7	0.8	16.6	0.3	9.1	-	3.04E+06
S211	57.2	11.2	Summer	Marine	23.8	1	17.4	5.7	0.0	1.0	0.0	0.1	0.2	3.10E+06
S213	57.2	11.2	Summer	Marine	30.9	20	13.7	5.4	0.1	2.0	0.2	0.5	0.4	2.39E+06
S214	57.2	11.2	Summer	Marine	33.0	40	9.8	5.1	0.5	9.2	0.4	6.8	0.2	2.64E+06
S215	57.2	11.2	Summer	Marine	34.1	66	7.6	4.7	0.9	17.2	0.2	10.0	0.2	2.64E+06
**S216**	**57.7**	**11.1**	**Summer**	**Marine**	**26.9**	**1**	**17.0**	**5.9**	**0.0**	**1.2**	**0.0**	**0.1**	**-**	**3.37E+06**
S218	57.7	11.1	Summer	Marine	31.0	20	14.8	5.7	0.0	1.8	0.0	0.2	-	1.93E+06
S219	57.7	11.1	Summer	Marine	31.9	25	14.4	5.6	-	-	-	-	-	1.92E+06
S220	57.7	11.1	Summer	Marine	32.5	34	14.3	5.3	0.1	4.0	0.1	0.8	-	1.96E+06
S221	58.2	10.3	Summer	Marine	30.6	1	16.6	5.7	0.0	0.9	0.0	0.1	0.4	3.41E+06
S222	58.2	10.3	Summer	Marine	33.5	20	10.2	7.3	0.0	0.3	0.0	0.1	0.2	6.61E+06
S223	58.2	10.3	Summer	Marine	35.2	60	7.6	5.9	0.7	4.1	0.0	10.0	0.2	1.45E+06
S224	58.2	10.3	Summer	Marine	35.2	100	7.6	5.9	0.7	4.1	0.0	10.0	0.2	1.45E+06
S225	58.2	10.3	Summer	Marine	35.2	179	7.1	5.8	0.9	7.1	0.1	11.4	0.2	1.11E+06
**S226**	**58.4**	**10.3**	**Summer**	**Marine**	**30.9**	**1**	**16.9**	**5.9**	**0.0**	**1.0**	**0.0**	**0.1**	**0.2**	**-**
S227	58.4	10.3	Summer	Marine	33.8	20	9.8	6.8	0.0	0.6	0.0	0.1	0.2	-
S24	55.3	17.8	Summer	Mesohaline	7.5	40	4.8	7.6	0.5	14.7	0.0	0.0	-	5.96E+05
S25	55.3	17.8	Summer	Marine	8.3	60	4.8	6.2	1.1	21.5	0.3	2.5	-	4.92E+05
S26	55.3	17.8	Summer	Marine	11.3	70	5.9	3.7	1.5	32.8	0.0	7.0	-	5.98E+05
**S27**	**54.9**	**19.3**	**Summer**	**Mesohaline**	**7.2**	**10**	**15.3**	**7.3**	**0.1**	**15.0**	**0.0**	**0.0**	**0.2**	**2.88E+06**
S28	54.9	19.3	Summer	Mesohaline	7.5	40	4.8	7.6	0.5	14.8	0.0	0.0	0.2	4.95E+05
S29	54.9	19.3	Summer	Marine	8.4	70	4.7	5.1	1.5	26.2	0.1	5.7	0.7	6.20E+05
**S3**	**54.9**	**13.5**	**Summer**	**Mesohaline**	**7.7**	**10**	**15.5**	**7.0**	**0.2**	**12.0**	**0.0**	**0.1**	**?**	**1.74E+06**
**S31**	**55.6**	**18.4**	**Summer**	**Mesohaline**	**7.4**	**10**	**14.7**	**6.9**	**0.3**	**15.3**	**0.0**	**0.0**	**0.2**	**3.32E+06**
S32	55.6	18.4	Summer	Mesohaline	7.4	40	4.6	7.8	0.5	15.7	0.0	0.1	0.4	4.03E+05
S36	55.9	18.9	Summer	Mesohaline	7.4	40	4.4	8.0	0.6	14.6	0.0	0.0	-	4.38E+05
S37	55.9	18.9	Summer	Marine	8.6	75	4.9	7.8	0.7	15.3	0.3	1.2	-	6.39E+05
S38	55.9	18.9	Summer	Marine	11.5	110	6.1	3.4	1.7	37.6	0.1	6.4	-	8.27E+05
**S39**	**56.9**	**19.7**	**Summer**	**Mesohaline**	**7.3**	**10**	**14.8**	**7.0**	**0.1**	**9.8**	**0.0**	**0.0**	**-**	**4.47E+06**
S4	54.9	13.5	Summer	Marine	11.1	30	13.1	6.3	-	-	-	-	-	9.94E+05
S40	56.9	19.7	Summer	Marine	8.8	70	4.9	7.3	0.8	15.4	0.4	1.0	-	4.07E+05
S45	57.3	20.1	Summer	Mesohaline	7.1	20	6.2	7.4	0.3	8.5	0.0	0.0	0.1	1.41E+06
S50	57.3	20.1	Summer	Mesohaline	7.4	48	4.3	7.9	0.5	13.0	0.0	0.1	0.5	1.48E+06
S51	57.3	20.1	Summer	Marine	8.1	70	4.4	5.5	1.4	22.7	0.3	3.5	0.9	4.83E+05
S58	57.8	20.1	Summer	Mesohaline	6.2	10	14.5	6.9	0.1	8.3	0.0	0.0	-	3.87E+06
S59	57.8	20.1	Summer	Marine	8.5	70	4.7	5.0	-	-	-	-	-	4.93E+05
S66	60.2	19.1	Summer	Mesohaline	5.0	10	13.0	7.3	-	-	-	-	-	2.30E+06
**S67**	**60.2**	**19.1**	**Summer**	**Mesohaline**	**4.9**	**2**	**13.5**	**7.3**	**-**	**-**	**-**	**-**	**-**	**4.34E+06**
S68	60.2	19.1	Summer	Mesohaline	5.8	20	5.3	8.3	-	-	-	-	-	1.65E+06
S69	60.2	19.1	Summer	Mesohaline	6.6	80	3.3	8.2	-	-	-	-	-	7.61E+05
S7	54.9	15.4	Summer	Marine	8.5	50	5.0	6.9	-	-	-	-	-	4.18E+05
S70	60.2	19.1	Summer	Mesohaline	7.0	250	4.2	7.3	-	-	-	-	-	9.08E+05
**S71**	**62.2**	**19.7**	**Summer**	**Mesohaline**	**5.3**	**2**	**12.2**	**7.8**	**0.0**	**8.9**	**0.0**	**0.0**	**-**	**3.38E+06**
S72	62.2	19.7	Summer	Mesohaline	5.3	10	11.3	7.8	0.0	9.0	0.0	0.0	-	3.18E+06
S73	62.2	19.7	Summer	Mesohaline	5.9	80	2.8	7.0	0.5	27.9	0.2	5.1	-	1.18E+06
**S75**	**63.1**	**20.5**	**Summer**	**Mesohaline**	**5.3**	**2**	**12.2**	**7.3**	**0.0**	**13.6**	**0.0**	**0.0**	**-**	**3.83E+06**
S79	65.4	23.5	Summer	Oligohaline	2.6	2	12.2	7.9	0.0	42.3	0.2	1.8	0.2	2.62E+06

Sample	Latitude	Longitude	Season	Salinity category	salinity	Depth (m)	Temperature (°C)	O_2 (ml/l)	PO_4 (µM)	SiO_2 (µM)	NO_2 (µM)	NO_3 (µM)	NH_4 (µM)	BC (cells/ml)
S8	54.9	15.4	Summer	Marine	14.7	70	6.7	2.0	-	-	-	-	-	6.81E+05
S80	65.4	23.5	Summer	Oligohaline	2.7	7.5	12.7	5.4	-	-	-	-	-	2.67E+06
S83	64.7	22.1	Summer	Oligohaline	2.8	2	12.3	7.9	0.0	34.9	0.2	2.7	-	2.64E+06
S84	64.7	22.1	Summer	Oligohaline	2.8	10	9.1	8.3	-	-	-	-	-	3.24E+06
S87	63.6	21.1	Summer	Oligohaline	3.2	2	12.6	7.9	-	-	-	-	-	4.31E+06
S88	63.6	21.1	Summer	Oligohaline	3.5	10	11.6	7.8	-	-	-	-	-	3.78E+06
**S9**	**55.3**	**16.0**	**Summer**	**Mesohaline**	**7.5**	**10**	**15.0**	**7.1**	**0.3**	**15.5**	**0.0**	**0.1**	**-**	**2.04E+08**
S90	63.6	21.1	Summer	Mesohaline	4.7	30	5.1	7.4	-	-	-	-	-	2.79E+06
S91	62.6	19.3	Summer	Mesohaline	4.9	2	13.3	7.5	0.0	9.3	0.0	0.0	-	6.47E+06
S92	62.6	19.3	Summer	Mesohaline	5.1	20	8.7	7.5	0.0	9.1	0.0	0.0	-	4.85E+06
S93	62.6	19.3	Summer	Mesohaline	5.4	40	2.6	8.5	0.1	16.1	0.2	0.8	-	2.28E+06
S94	62.6	19.3	Summer	Mesohaline	6.1	105	3.6	5.6	0.8	36.3	0.1	7.1	-	1.61E+06
**S96**	**62.6**	**20.0**	**Summer**	**Mesohaline**	**4.8**	**10**	**12.2**	**7.5**	**0.0**	**12.7**	**0.0**	**0.0**	**0.2**	**4.35E+06**
W003	54.8	10.9	Winter	Marine	24.0	15	4.9	8.1	0.8	11.1	0.2	6.8	0.2	7.12E+05
W004	55.3	11.1	Winter	Marine	12.8	1	1.7	?	0.3	5.2	0.1	1.6	0.8	1.09E+06
W006	55.3	11.1	Winter	Marine	29.2	25	6.2	7.4	0.9	13.8	0.2	9.1	0.3	7.46E+05
W007	55.7	10.8	Winter	Marine	15.8	1	1.8	11.8	0.3	1.0	0.0	0.0	0.2	1.06E+06
W009	55.7	10.8	Winter	Marine	31.0	28	6.4	7.4	1.1	8.6	0.3	7.4	0.1	8.50E+05
W010	56.2	11.1	Winter	Marine	18.7	1	1.5	11.8	0.1	0.0	0.1	0.0	0.0	9.99E+05
W012	56.2	11.1	Winter	Marine	32.4	16	6.5	7.4	0.2	21.7	0.1	1.7	15.9	7.60E+05
W016	56.7	11.1	Winter	Marine	29.5	13	5.6	7.9	0.6	10.1	0.2	5.8	0.0	7.35E+05
W018	57.2	11.7	Winter	Marine	34.3	21	5.6	8.3	0.6	8.4	0.0	6.7	0.0	6.02E+05
W019	57.2	11.7	Winter	Marine	34.7	50	6.2	8.1	0.5	6.4	0.0	15.9	0.0	4.67E+05
**W021**	**57.7**	**11.1**	**Winter**	**Marine**	**20.2**	**1**	**1.0**	**11.1**	**0.2**	**2.4**	**0.1**	**0.4**	**0.0**	**1.01E+06**
W023	57.7	11.1	Winter	Marine	32.2	13	3.2	9.5	1.0	7.0	0.3	8.3	0.3	6.39E+05
W025	57.7	11.1	Winter	Marine	34.1	34	4.3	9.0	0.6	7.0	0.3	7.9	0.4	6.53E+05
W027	58.1	10.3	Winter	Marine	34.1	20	5.1	8.8	0.5	3.3	0.1	7.0	0.8	8.20E+05
W028	58.1	10.3	Winter	Marine	35.0	60	6.3	8.2	0.7	4.4	0.0	8.5	0.0	4.81E+05
W030	58.1	10.3	Winter	Marine	35.0	177	5.5	8.6	0.7	3.5	0.1	7.5	1.0	5.38E+05
**W031**	**58.4**	**10.3**	**Winter**	**Marine**	**27.0**	**1**	**1.5**	**11.6**	**0.1**	**0.0**	**0.1**	**0.2**	**33.8**	**9.72E+05**
W032	58.4	10.3	Winter	Marine	34.7	26	6.4	8.3	0.6	1.9	0.1	7.5	32.5	5.14E+05
W033	58.4	10.3	Winter	Marine	35.0	100	6.9	7.9	0.7	2.0	0.0	8.3	22.6	4.68E+05
W034	58.4	10.3	Winter	Marine	35.2	300	7.2	7.6	0.8	2.7	0.0	10.6	25.3	3.53E+05
**W035**	**56.9**	**11.9**	**Winter**	**Marine**	**19.4**	**1**	**1.3**	**11.7**	**0.2**	**1.6**	**0.1**	**0.1**	**6.3**	**1.10E+06**
W036	56.9	11.9	Winter	Marine	33.4	20	5.4	8.3	0.6	6.8	0.1	7.3	4.7	6.47E+05
W037	56.9	11.9	Winter	Marine	34.4	36	5.8	8.1	0.5	7.5	0.1	5.5	4.7	5.76E+05
W039	56.4	12.3	Winter	Marine	31.8	15	5.7	8.2	0.7	7.4	0.0	7.2	0.3	7.03E+05
W042	55.1	13.3	Winter	Marine	8.4	25	2.3	11.2	0.7	5.8	0.4	2.8	0.0	5.79E+05
W043	55.1	13.3	Winter	Marine	10.1	38	2.8	ND	0.7	7.4	0.5	3.0	1.0	6.33E+05
W045	55.2	14.4	Winter	Marine	8.4	25	2.6	10.9	0.7	10.0	0.4	3.3	0.6	6.04E+05
W046	55.2	14.4	Winter	Marine	14.1	42	4.5	8.9	0.8	17.8	0.2	5.8	0.3	8.83E+05
**W047**	**55.5**	**14.9**	**Winter**	**Mesohaline**	**7.7**	**2**	**3.0**	**10.9**	**0.7**	**15.9**	**0.1**	**2.7**	**0.8**	**6.87E+05**
W048	55.5	14.9	Winter	Mesohaline	7.7	30	3.0	10.9	0.7	11.2	0.1	2.9	1.3	6.86E+05
W049	55.5	14.9	Winter	Mesohaline	7.9	50	3.3	10.7	0.7	16.3	0.1	3.2	0.6	7.05E+05
**W052**	**55.5**	**15.6**	**Winter**	**Mesohaline**	**7.8**	**2**	**3.1**	**10.9**	**4.1**	**10.6**	**0.1**	**2.7**	**1.3**	**6.85E+05**
W053	55.5	15.6	Winter	Mesohaline	7.8	45	3.1	8.5	4.0	9.5	0.1	2.8	1.3	7.11E+05

Sample	Latitude	Longitude	Season	Salinity category	salinity	Depth (m)	Temperature (°C)	O_2 (ml/l)	PO_4 (µM)	SiO_2 (µM)	NO_2 (µM)	NO_3 (µM)	NH_4 (µM)	BC (cells/ml)
W055	55.9	16.5	Winter	Mesohaline	7.4	2	2.6	11.1	0.7	8.1	0.1	2.0	0.1	6.24E+05
W056	55.9	16.5	Winter	Mesohaline	7.4	25	2.6	11.0	0.7	10.8	0.1	2.6	0.0	6.61E+05
W058	55.9	16.5	Winter	Mesohaline	7.2	1	2.5	11.0	0.6	29.4	0.1	2.8	0.0	4.75E+05
W059	56.3	17.0	Winter	Mesohaline	7.2	30	2.5	11.0	0.7	29.4	0.2	2.7	0.2	5.13E+05
W060	56.3	17.0	Winter	Mesohaline	7.2	51	2.5	10.9	0.6	29.9	0.2	1.6	1.8	4.61E+05
**W061**	**56.8**	**17.4**	**Winter**	**Mesohaline**	**7.1**	**1**	**2.7**	**10.9**	**0.6**	**7.7**	**0.1**	**3.4**	**0.0**	**4.08E+05**
W062	56.8	17.4	Winter	Mesohaline	7.2	53	2.8	10.8	0.7	8.5	0.1	3.4	0.0	4.26E+05
W063	56.8	17.4	Winter	Marine	9.6	86	5.2	nd	3.4	40.9	0.1	0.0	4.2	2.09E+06
W064	57.2	17.6	Winter	Mesohaline	7.1	1	2.7	10.9	0.7	14.1	0.1	3.4	0.0	3.92E+05
W065	57.2	17.6	Winter	Mesohaline	7.1	50	2.5	10.9	0.7	14.6	0.1	3.5	0.8	4.92E+05
W066	57.2	17.6	Winter	Marine	8.4	74	5.0	4.1	1.9	32.5	0.0	4.3	0.1	5.22E+05
**W067**	**57.7**	**17.4**	**Winter**	**Mesohaline**	**7.0**	**1**	**2.6**	**11.0**	**0.7**	**13.7**	**0.1**	**3.6**	**nd**	**3.91E+05**
W068	57.7	17.4	Winter	Mesohaline	7.1	58	2.4	10.3	0.8	15.5	0.1	3.6	0.3	3.19E+05
W072	58.2	17.7	Winter	Mesohaline	6.9	1	2.2	11.1	0.6	15.2	0.2	3.4	0.7	2.92E+05
W073	58.2	17.7	Winter	Mesohaline	7.0	50	2.6	10.9	0.6	14.5	0.2	3.1	0.0	3.04E+05
W076	58.6	18.2	Winter	Mesohaline	6.5	1	1.7	11.3	0.6	11.7	0.1	3.7	1.2	4.74E+05
W077	58.6	18.2	Winter	Mesohaline	7.0	32	2.5	10.9	0.6	35.4	0.1	3.5	0.2	3.16E+05
W078	58.6	18.2	Winter	Mesohaline	7.1	50	2.9	8.8	3.2	9.2	0.1	4.0	0.0	2.95E+05
W084	58.9	19.1	Winter	Mesohaline	7.0	52	2.8	10.8	0.6	13.9	0.1	3.9	0.0	3.03E+05
W086	59.3	19.5	Winter	Mesohaline	6.4	1	1.9	11.3	0.6	14.9	0.1	3.2	0.1	4.31E+05
W087	59.3	19.5	Winter	Mesohaline	6.8	39	2.7	10.9	0.7	8.4	0.1	3.7	0.2	2.46E+05
W088	59.3	19.5	Winter	Mesohaline	7.6	61	4.3	8.4	1.1	15.1	0.0	3.7	0.0	3.62E+05
**W089**	**60.2**	**19.1**	**Winter**	**Mesohaline**	**5.4**	**1**	**0.2**	**12.1**	**0.4**	**20.1**	**0.1**	**3.0**	**0.1**	**8.16E+05**
W090	60.2	19.1	Winter	Mesohaline	6.4	50	3.6	10.2	0.6	16.2	0.1	4.1	0.2	2.94E+05
W091	60.2	19.1	Winter	Mesohaline	6.6	100	4.0	10.0	0.7	14.6	0.0	3.9	0.0	3.08E+05
W092	60.2	19.1	Winter	Mesohaline	7.1	256	5.8	6.9	1.3	24.0	0.1	4.3	0.1	7.46E+05
**W093**	**62.1**	**19.7**	**Winter**	**Mesohaline**	**5.4**	**1**	**1.0**	**11.6**	**0.4**	**18.9**	**0.0**	**3.5**	**0.1**	**8.87E+05**
W095	62.1	19.7	Winter	Mesohaline	6.3	113	4.3	6.7	0.3	35.8	0.4	5.2	0.2	7.39E+05
**W096**	**62.6**	**20.0**	**Winter**	**Mesohaline**	**5.5**	**1**	**1.0**	**11.6**	**0.3**	**18.3**	**0.0**	**3.6**	**0.0**	**8.64E+05**
W097	62.6	20.0	Winter	Mesohaline	5.5	49	1.3	11.5	0.3	17.4	0.0	3.6	0.0	8.52E+05
W098	62.6	20.0	Winter	Mesohaline	5.7	80	3.2	10.2	0.4	19.6	0.0	3.4	0.0	8.24E+05
W099	62.6	20.0	Winter	Mesohaline	6.3	130	4.5	7.2	1.1	32.1	0.1	4.6	0.6	7.02E+05
**W102**	**63.0**	**20.5**	**Winter**	**Mesohaline**	**5.5**	**1**	**0.2**	**11.8**	**0.0**	**17.0**	**0.1**	**4.3**	**0.4**	**8.89E+05**
W103	63.0	20.5	Winter	Mesohaline	5.5	15	0.6	11.7	0.0	16.9	0.1	4.0	0.4	9.47E+05
W104	63.0	20.5	Winter	Mesohaline	5.6	20	0.8	11.6	0.0	13.4	0.1	3.7	0.1	8.72E+05
W105	63.0	20.5	Winter	Mesohaline	5.6	50	1.5	11.4	0.0	12.9	0.0	2.9	0.4	8.94E+05
W107	59.9	25.6	Winter	Mesohaline	5.1	1	-0.1	11.7	0.9	22.2	0.0	8.1	0.1	-
W109	59.9	25.6	Winter	Marine	9.1	70	5.6	2.2	2.5	46.4	0.0	5.4	0.1	1.10E+06
W111	59.8	24.8	Winter	Mesohaline	5.2	1	0.1	12.0	0.9	18.1	0.0	6.8	0.1	1.12E+06
W112	59.8	24.8	Winter	Mesohaline	5.3	15	0.3	11.8	0.9	18.0	0.0	8.2	0.7	1.11E+06
W113	59.8	24.8	Winter	Mesohaline	6.2	35	1.4	11.3	0.8	15.6	0.1	5.4	0.0	4.31E+05
W114	59.8	24.8	Winter	Mesohaline	6.9	50	3.5	9.9	0.9	17.0	0.0	4.7	0.1	3.91E+05
W115	59.8	24.8	Winter	Marine	9.3	70	5.4	2.1	2.7	44.5	0.1	4.2	0.1	9.54E+05
W117	59.7	24.0	Winter	Mesohaline	5.9	15	0.9	11.5	0.8	13.3	0.1	7.7	0.1	7.70E+05
W118	59.7	24.0	Winter	Mesohaline	7.2	62	4.1	9.4	0.9	12.6	0.0	4.6	0.1	3.53E+05
W121	59.7	24.0	Winter	Mesohaline	6.4	15	1.6	11.3	0.8	15.2	0.1	6.4	0.0	4.28E+05
W122	59.7	24.0	Winter	Marine	9.2	65	5.7	2.5	2.5	39.9	0.0	4.8	0.0	2.87E+05
W124	59.5	21.0	Winter	Mesohaline	6.3	2	1.4	11.5	0.9	11.2	0.1	6.5	0.3	5.27E+05

Sample	Latitude	Longitude	Season	Salinity category	salinity	Depth (m)	Temperature (°C)	O_2 (ml/l)	PO_4 (µM)	SiO_2 (µM)	NO_2 (µM)	NO_3 (µM)	NH_4 (µM)	BC (cells/ml)
W125	59.5	21.0	Winter	Mesohaline	6.4	20	1.5	11.4	0.9	14.1	0.1	5.3	0.1	3.84E+05
W126	59.5	21.0	Winter	Mesohaline	6.6	45	2.0	11.1	0.8	11.3	0.1	6.3	0.0	3.62E+05
W127	59.5	21.0	Winter	Mesohaline	6.7	54	2.3	10.7	0.8	10.6	0.1	5.3	0.3	4.29E+05
W128	59.2	21.4	Winter	Mesohaline	7.0	2	2.5	11.1	0.4	13.1	0.1	4.3	0.1	3.41E+05
W129	59.2	21.4	Winter	Mesohaline	7.0	40	2.4	11.0	0.4	13.2	0.1	4.7	0.1	3.17E+05
W134	58.3	20.4	Winter	Mesohaline	7.5	65	3.5	10.3	0.7	7.0	0.0	3.6	4.2	5.05E+05
W136	57.8	20.1	Winter	Mesohaline	7.2	15	3.0	10.9	0.6	11.6	0.1	3.7	6.7	2.72E+05
W137	57.8	20.1	Winter	Mesohaline	7.3	60	3.1	10.8	0.6	9.0	0.1	3.4	1.3	3.50E+05
W141	57.3	20.1	Winter	Mesohaline	7.3	2	3.1	11.0	0.6	10.9	0.1	1.9	0.0	4.04E+05
W142	57.3	20.1	Winter	Mesohaline	7.3	25	3.1	11.0	0.6	10.1	0.1	2.2	0.0	4.69E+05
W143	57.3	20.1	Winter	Mesohaline	8.0	60	4.5	9.3	0.9	14.2	0.1	2.8	0.0	5.35E+05
**W145**	**56.8**	**19.7**	**Winter**	**Mesohaline**	**7.4**	**2.5**	**3.2**	**11.1**	**0.6**	**10.8**	**0.1**	**4.0**	**0.1**	**5.94E+05**
W146	56.8	19.7	Winter	Mesohaline	7.6	45	3.3	10.9	0.6	9.6	0.1	3.0	0.4	6.59E+05
W149	55.9	19.0	Winter	Mesohaline	7.5	1.5	3.4	11.0	0.6	10.0	0.1	2.9	0.0	5.30E+05
W151	55.9	19.0	Winter	Mesohaline	7.6	60	3.5	10.8	0.7	9.1	0.1	3.2	0.0	5.58E+05
W153	55.9	19.0	Winter	Marine	11.8	108	7.5	2.4	2.3	39.0	0.1	6.4	0.0	6.66E+05
**W155**	**55.5**	**18.4**	**Winter**	**Mesohaline**	**7.4**	**1**	**3.4**	**11.0**	**0.6**	**11.4**	**0.1**	**2.7**	**0.0**	**5.99E+05**
W156	55.5	18.4	Winter	Mesohaline	7.5	45	3.4	11.0	0.6	11.3	0.1	2.8	0.2	5.42E+05
**W159**	**54.9**	**19.3**	**Winter**	**Mesohaline**	**7.5**	**1**	**2.9**	**11.5**	**0.6**	**12.2**	**0.2**	**5.3**	**1.0**	**9.99E+05**
W160	54.9	19.3	Winter	Mesohaline	7.8	60	3.3	10.7	0.7	9.6	0.0	3.5	0.0	6.62E+05
W163	55.3	17.8	Winter	Mesohaline	7.6	2	3.3	10.9	0.6	7.3	0.1	3.3	0.3	6.68E+05
W165	55.3	17.8	Winter	Marine	12.2	70	7.7	5.1	1.4	19.3	0.0	6.1	1.2	6.72E+05
W166	55.3	17.8	Winter	Marine	13.3	78	8.6	4.3	1.5	22.4	0.0	7.2	0.2	6.11E+05
**W167**	**55.2**	**16.0**	**Winter**	**Mesohaline**	**7.9**	**1.5**	**3.2**	**11.0**	**0.6**	**7.8**	**0.1**	**3.0**	**0.1**	**9.30E+05**
W172	54.9	15.4	Winter	Mesohaline	7.8	30	2.8	11.1	0.6	10.9	0.2	2.0	0.7	9.01E+05
W173	54.9	15.4	Winter	Marine	12.5	65	8.8	3.9	1.5	30.7	0.0	5.9	0.8	5.64E+05
W174	54.9	15.4	Winter	Marine	13.6	68	9.3	3.3	1.7	30.5	0.1	6.0	0.0	7.12E+05
W176	54.9	13.5	Winter	Marine	8.2	20	2.5	11.2	0.6	8.2	0.3	1.7	1.2	7.23E+05
W177	54.9	13.5	Winter	Marine	12.8	42	2.7	10.2	0.6	8.5	0.3	2.0	0.5	6.96E+05
W178	54.9	13.5	Winter	Marine	15.6	45	2.9	10.2	0.5	4.0	0.2	0.3	0.9	5.26E+05
W180	55.0	13.4	Winter	Marine	8.8	30	2.3	11.8	0.3	1.0	0.0	0.0	0.1	6.78E+05
W181	55.0	13.4	Winter	Marine	12.7	40	2.6	10.5	0.6	6.7	0.3	2.2	31.0	7.20E+05
**W182**	**54.9**	**13.3**	**Winter**	**Mesohaline**	**7.9**	**1.5**	**2.7**	**-**	**-**	**-**	**-**	**-**	**-**	**1.01E+06**
W183	54.9	13.3	Winter	Marine	8.2	20	2.5	-	-	-	-	-	-	7.75E+05
W184	54.9	13.3	Winter	Marine	11.1	40	2.7	-	-	-	-	-	-	6.48E+05
W185	54.7	12.7	Winter	Marine	8.5	1	2.8	11.5	0.5	8.0	0.1	1.0	0.0	9.03E+05
W187	54.7	12.7	Winter	Marine	12.2	19	2.8	10.0	0.9	10.7	0.3	3.6	0.7	1.04E+06
**W188**	**54.4**	**12.1**	**Winter**	**Marine**	**10.1**	**5**	**2.6**	**11.9**	**0.2**	**0.8**	**0.0**	**0.0**	**0.0**	**7.41E+05**
W190	54.4	12.1	Winter	Marine	16.2	19	3.0	9.6	0.7	8.6	0.1	1.9	2.6	1.28E+06

Table S2: Results of the LEfSe analysis

Taxonomy	LDA	Envrion-	Wilcoxon	Kruskal-
Actinobacteria.Acidimicrobiia.Acidimicrobiales.Acidimicrobiaceae.uncultured.OTU99_000282	2.85	Marine	2.69	0.01
Actinobacteria.Acidimicrobiia.Acidimicrobiales.OM1_clade	4.02	Marine	3.73	0.00
Actinobacteria.Acidimicrobiia.Acidimicrobiales.OM1_clade.Candidatus_Actinomarina	4.01	Marine	3.73	0.00
Actinobacteria.Acidimicrobiia.Acidimicrobiales.OM1_clade.Candidatus_Actinomarina.OTU99_000050	4.00	Marine	3.72	0.00
Actinobacteria.Acidimicrobiia.Acidimicrobiales.Sva0996_marine_group.unclassified.OTU99_000512	3.07	Marine	2.86	0.01
Actinobacteria.Acidimicrobiia.Acidimicrobiales.Sva0996_marine_group.unclassified.OTU99_001286	2.32	Marine	2.75	0.01
Actinobacteria.Acidimicrobiia.Acidimicrobiales.Sva0996_marine_group.unclassified.OTU99_001893	3.02	Marine	2.83	0.01
Actinobacteria.Acidimicrobiia.Acidimicrobiales.Sva0996_marine_group.unclassified.OTU99_002054	2.24	Marine	2.49	0.01
Actinobacteria.Acidimicrobiia.Acidimicrobiales.Sva0996_marine_group.unclassified.OTU99_002237	2.24	Marine	2.48	0.01
Actinobacteria.Acidimicrobiia.Acidimicrobiales.Sva0996_marine_group.unclassified.OTU99_002916	2.66	Marine	2.65	0.01
Actinobacteria.Acidimicrobiia.Acidimicrobiales.Sva0996_marine_group.unclassified.OTU99_004196	2.70	Marine	2.72	0.00
Actinobacteria.Acidimicrobiia.Acidimicrobiales.Sva0996_marine_group.unclassified.OTU99_024022	2.16	Marine	3.48	0.01
Actinobacteria.Actinobacteria.Corynebacteriales.Nocardiaceae	2.53	Marine	2.76	0.02
Actinobacteria.Actinobacteria.Corynebacteriales.Nocardiaceae.Rhodococcus	2.53	Marine	2.77	0.02
Actinobacteria.Actinobacteria.PeM15.unclassified.unclassified.OTU99_000142	3.86	Marine	3.40	0.02
Actinobacteria.unclassified.unclassified.unclassified.unclassified.OTU99_000510	3.52	Marine	3.23	0.00
Bacteroidetes.Cytophagia.Cytophagales.Flammeovirgaceae	3.52	Marine	3.14	0.01
Bacteroidetes.Cytophagia.Cytophagales.Flammeovirgaceae.Marinoscillum	3.13	Marine	2.85	0.00
Bacteroidetes.Cytophagia.Cytophagales.Flammeovirgaceae.Marinoscillum.OTU99_000381	2.83	Marine	2.58	0.01
Bacteroidetes.Cytophagia.Cytophagales.Flammeovirgaceae.Marinoscillum.OTU99_000867	2.53	Marine	2.44	0.01
Bacteroidetes.Cytophagia.Cytophagales.Flammeovirgaceae.Marinoscillum.OTU99_005596	2.24	Marine	2.49	0.01
Bacteroidetes.Flavobacteriia.Flavobacteriales.Cryomorphaceae.Fluviicola.OTU99_000245	3.64	Marine	3.35	0.00
Bacteroidetes.Flavobacteriia.Flavobacteriales.Cryomorphaceae.Fluviicola.OTU99_003026	2.46	Marine	2.64	0.01
Bacteroidetes.Flavobacteriia.Flavobacteriales.Cryomorphaceae.NS10_marine_group	3.06	Marine	2.88	0.00
Bacteroidetes.Flavobacteriia.Flavobacteriales.Cryomorphaceae.NS10_marine_group.OTU99_000951	2.84	Marine	2.88	0.00
Bacteroidetes.Flavobacteriia.Flavobacteriales.Cryomorphaceae.Owenweeksia.OTU99_000290	3.14	Marine	2.93	0.00
Bacteroidetes.Flavobacteriia.Flavobacteriales.Cryomorphaceae.Owenweeksia.OTU99_001389	2.85	Marine	2.60	0.01
Bacteroidetes.Flavobacteriia.Flavobacteriales.Cryomorphaceae.Owenweeksia.OTU99_007473	2.42	Marine	2.42	0.01
Bacteroidetes.Flavobacteriia.Flavobacteriales.Cryomorphaceae.unclassified.OTU99_000505	3.05	Marine	2.84	0.01
Bacteroidetes.Flavobacteriia.Flavobacteriales.Flavobacteriaceae.Formosa	3.61	Marine	3.30	0.00
Bacteroidetes.Flavobacteriia.Flavobacteriales.Flavobacteriaceae.Formosa.OTU99_000114	3.23	Marine	2.97	0.00
Bacteroidetes.Flavobacteriia.Flavobacteriales.Flavobacteriaceae.Formosa.OTU99_000330	2.89	Marine	2.71	0.01
Bacteroidetes.Flavobacteriia.Flavobacteriales.Flavobacteriaceae.Formosa.OTU99_001139	3.12	Marine	2.85	0.00
Bacteroidetes.Flavobacteriia.Flavobacteriales.Flavobacteriaceae.NS2b_marine_group	2.76	Marine	2.65	0.01
Bacteroidetes.Flavobacteriia.Flavobacteriales.Flavobacteriaceae.NS2b_marine_group.OTU99_001835	2.46	Marine	2.61	0.01
Bacteroidetes.Flavobacteriia.Flavobacteriales.Flavobacteriaceae.NS2b_marine_group.OTU99_008491	2.46	Marine	2.62	0.01
Bacteroidetes.Flavobacteriia.Flavobacteriales.Flavobacteriaceae.NS3a_marine_group	4.41	Marine	3.72	0.03
Bacteroidetes.Flavobacteriia.Flavobacteriales.Flavobacteriaceae.NS4_marine_group	4.07	Marine	3.73	0.00
Bacteroidetes.Flavobacteriia.Flavobacteriales.Flavobacteriaceae.NS4_marine_group.OTU99_000127	3.76	Marine	3.47	0.00
Bacteroidetes.Flavobacteriia.Flavobacteriales.Flavobacteriaceae.NS4_marine_group.OTU99_000174	2.95	Marine	2.77	0.00
Bacteroidetes.Flavobacteriia.Flavobacteriales.Flavobacteriaceae.NS4_marine_group.OTU99_000522	3.17	Marine	2.95	0.00
Bacteroidetes.Flavobacteriia.Flavobacteriales.Flavobacteriaceae.NS4_marine_group.OTU99_001276	2.33	Marine	2.73	0.01
Bacteroidetes.Flavobacteriia.Flavobacteriales.Flavobacteriaceae.NS4_marine_group.OTU99_002742	2.92	Marine	2.71	0.01
Bacteroidetes.Flavobacteriia.Flavobacteriales.Flavobacteriaceae.NS5_marine_group	4.48	Marine	4.03	0.01
Bacteroidetes.Flavobacteriia.Flavobacteriales.Flavobacteriaceae.NS5_marine_group.OTU99_000119	3.88	Marine	3.48	0.01
Bacteroidetes.Flavobacteriia.Flavobacteriales.Flavobacteriaceae.NS5_marine_group.OTU99_000135	3.71	Marine	3.40	0.00
Bacteroidetes.Flavobacteriia.Flavobacteriales.Flavobacteriaceae.NS5_marine_group.OTU99_000272	3.65	Marine	3.34	0.00
Bacteroidetes.Flavobacteriia.Flavobacteriales.Flavobacteriaceae.NS5_marine_group.OTU99_000447	2.84	Marine	2.68	0.01
Bacteroidetes.Flavobacteriia.Flavobacteriales.Flavobacteriaceae.NS5_marine_group.OTU99_000615	2.97	Marine	2.95	0.01
Bacteroidetes.Flavobacteriia.Flavobacteriales.Flavobacteriaceae.NS5_marine_group.OTU99_000643	3.11	Marine	2.84	0.01
Bacteroidetes.Flavobacteriia.Flavobacteriales.Flavobacteriaceae.NS5_marine_group.OTU99_000887	2.84	Marine	2.74	0.00
Bacteroidetes.Flavobacteriia.Flavobacteriales.Flavobacteriaceae.NS5_marine_group.OTU99_000902	2.78	Marine	2.56	0.01
Bacteroidetes.Flavobacteriia.Flavobacteriales.Flavobacteriaceae.NS5_marine_group.OTU99_000904	3.22	Marine	2.93	0.01
Bacteroidetes.Flavobacteriia.Flavobacteriales.Flavobacteriaceae.NS5_marine_group.OTU99_001144	2.88	Marine	2.68	0.01
Bacteroidetes.Flavobacteriia.Flavobacteriales.Flavobacteriaceae.NS5_marine_group.OTU99_002698	2.32	Marine	2.75	0.01
Bacteroidetes.Flavobacteriia.Flavobacteriales.Flavobacteriaceae.NS5_marine_group.OTU99_003029	2.53	Marine	2.86	0.01
Bacteroidetes.Flavobacteriia.Flavobacteriales.Flavobacteriaceae.NS5_marine_group.OTU99_004257	2.54	Marine	2.78	0.01
Bacteroidetes.Flavobacteriia.Flavobacteriales.Flavobacteriaceae.NS5_marine_group.OTU99_004578	2.29	Marine	2.95	0.01
Bacteroidetes.Flavobacteriia.Flavobacteriales.Flavobacteriaceae.NS5_marine_group.OTU99_010327	2.37	Marine	2.77	0.01
Bacteroidetes.Flavobacteriia.Flavobacteriales.Flavobacteriaceae.Ulvibacter.OTU99_000157	3.95	Marine	3.60	0.00
Bacteroidetes.Flavobacteriia.Flavobacteriales.Flavobacteriaceae.unclassified.OTU99_000022	3.92	Marine	3.62	0.00
Bacteroidetes.Flavobacteriia.Flavobacteriales.Flavobacteriaceae.unclassified.OTU99_000182	3.61	Marine	3.33	0.00
Bacteroidetes.Flavobacteriia.Flavobacteriales.Flavobacteriaceae.unclassified.OTU99_000352	3.67	Marine	3.34	0.00
Bacteroidetes.Flavobacteriia.Flavobacteriales.Flavobacteriaceae.unclassified.OTU99_000398	3.20	Marine	3.07	0.01

Taxonomy	LDA	Envrion-ment	Wilcoxon rank-sum test	Kruskal-Wallis
Bacteroidetes.Flavobacteriia.Flavobacteriales.Flavobacteriaceae.unclassified.OTU99_000577	2.97	Marine	2.79	0.01
Bacteroidetes.Flavobacteriia.Flavobacteriales.Flavobacteriaceae.unclassified.OTU99_000706	2.95	Marine	2.80	0.00
Bacteroidetes.Flavobacteriia.Flavobacteriales.Flavobacteriaceae.unclassified.OTU99_000727	3.18	Marine	2.97	0.00
Bacteroidetes.Flavobacteriia.Flavobacteriales.Flavobacteriaceae.unclassified.OTU99_000874	2.55	Marine	2.71	0.01
Bacteroidetes.Flavobacteriia.Flavobacteriales.Flavobacteriaceae.unclassified.OTU99_003127	2.55	Marine	2.41	0.01
Bacteroidetes.Flavobacteriia.Flavobacteriales.Flavobacteriaceae.unclassified.OTU99_009894	2.24	Marine	2.49	0.01
Bacteroidetes.Flavobacteriia.Flavobacteriales.Flavobacteriaceae.uncultured.OTU99_000095	3.31	Marine	3.01	0.00
Bacteroidetes.Flavobacteriia.Flavobacteriales.NS7_marine_group	3.55	Marine	3.17	0.01
Bacteroidetes.Flavobacteriia.Flavobacteriales.NS7_marine_group.unclassified	3.55	Marine	3.18	0.01
Bacteroidetes.Flavobacteriia.Flavobacteriales.NS7_marine_group.unclassified.OTU99_000427	3.19	Marine	2.94	0.01
Bacteroidetes.Flavobacteriia.Flavobacteriales.NS7_marine_group.unclassified.OTU99_000970	2.96	Marine	2.88	0.00
Bacteroidetes.Flavobacteriia.Flavobacteriales.unclassified	4.26	Marine	3.90	0.00
Bacteroidetes.Flavobacteriia.Flavobacteriales.unclassified.unclassified	4.26	Marine	3.90	0.00
Bacteroidetes.Flavobacteriia.Flavobacteriales.unclassified.unclassified.OTU99_000144	3.68	Marine	3.40	0.00
Bacteroidetes.Flavobacteriia.Flavobacteriales.unclassified.unclassified.OTU99_000247	3.61	Marine	3.31	0.00
Bacteroidetes.Flavobacteriia.Flavobacteriales.unclassified.unclassified.OTU99_000390	3.21	Marine	2.89	0.01
Bacteroidetes.Flavobacteriia.Flavobacteriales.unclassified.unclassified.OTU99_000849	2.94	Marine	2.76	0.01
Bacteroidetes.Flavobacteriia.Flavobacteriales.unclassified.unclassified.OTU99_000862	2.47	Marine	2.90	0.01
Bacteroidetes.Flavobacteriia.Flavobacteriales.unclassified.unclassified.OTU99_001301	2.60	Marine	2.94	0.00
Bacteroidetes.Flavobacteriia.Flavobacteriales.unclassified.unclassified.OTU99_002925	2.79	Marine	2.58	0.01
Bacteroidetes.Flavobacteriia.Flavobacteriales.unclassified.unclassified.OTU99_003039	2.61	Marine	2.74	0.01
Bacteroidetes.Flavobacteriia.Flavobacteriales.unclassified.unclassified.OTU99_005949	2.51	Marine	2.62	0.01
Bacteroidetes.Flavobacteriia.Flavobacteriales.unclassified.unclassified.OTU99_007056	2.42	Marine	2.42	0.01
Bacteroidetes.Sphingobacteriia.Sphingobacteriales.NS11_12_marine_group.unclassified.OTU99_000790	2.78	Marine	2.63	0.01
Bacteroidetes.Sphingobacteriia.Sphingobacteriales.Saprospiraceae.Aureispira	2.93	Marine	2.93	0.00
Bacteroidetes.Sphingobacteriia.Sphingobacteriales.Saprospiraceae.unclassified.OTU99_000669	3.20	Marine	2.91	0.01
Bacteroidetes.Sphingobacteriia.Sphingobacteriales.Saprospiraceae.unclassified.OTU99_001494	2.71	Marine	2.85	0.00
Bacteroidetes.Sphingobacteriia.Sphingobacteriales.Saprospiraceae.unclassified.OTU99_002295	2.97	Marine	2.78	0.01
bacteroidetes.unclassified.unclassified.unclassified.unclassified.OTU99_000102	3.82	Marine	3.51	0.00
bacteroidetes.unclassified.unclassified.unclassified.unclassified.OTU99_001793	2.88	Marine	2.78	0.00
bacteroidetes.unclassified.unclassified.unclassified.unclassified.OTU99_001870	2.37	Marine	2.85	0.01
bacteroidetes.unclassified.unclassified.unclassified.unclassified.OTU99_003361	2.55	Marine	2.42	0.01
Chloroflexi.JG30_KF_CM66.unclassified.unclassified.unclassified.OTU99_013106	2.24	Marine	2.48	0.01
Chloroflexi.SAR202_clade.unclassified.unclassified.unclassified.OTU99_000344	3.40	Marine	3.14	0.01
Chloroflexi.SAR202_clade.unclassified.unclassified.unclassified.OTU99_000585	3.03	Marine	2.74	0.01
Chloroflexi.SAR202_clade.unclassified.unclassified.unclassified.OTU99_000737	3.35	Marine	3.10	0.01
Cyanobacteria.Cyanobacteria.SubsectionI.FamilyI.Synechococcus.OTU99_000010	4.39	Marine	4.07	0.00
Cyanobacteria.Cyanobacteria.SubsectionI.FamilyI.Synechococcus.OTU99_000041	4.05	Marine	3.75	0.00
Cyanobacteria.Cyanobacteria.SubsectionI.FamilyI.Synechococcus.OTU99_005496	2.58	Marine	2.58	0.01
Cyanobacteria.Cyanobacteria.SubsectionI.FamilyI.unclassified.OTU99_001020	3.18	Marine	2.93	0.00
Cyanobacteria.Cyanobacteria.SubsectionI.FamilyI.unclassified.OTU99_001528	2.87	Marine	2.80	0.01
Deferribacteres	3.98	Marine	3.70	0.00
Deferribacteres.Deferribacteres	3.98	Marine	3.70	0.00
Deferribacteres.Deferribacteres.Deferribacterales	3.98	Marine	3.70	0.00
Deferribacteres.Deferribacteres.Deferribacterales.SAR406_clade_Marine_group_A_	3.98	Marine	3.70	0.00
Deferribacteres.Deferribacteres.Deferribacterales.SAR406_clade_Marine_group_A_.unclassified	3.98	Marine	3.70	0.00
Deferribacteres.Deferribacteres.Deferribacterales.SAR406_clade_Marine_group_A_.unclassified.OTU99_0003[illegible]	3.63	Marine	3.32	0.00
Deferribacteres.Deferribacteres.Deferribacterales.SAR406_clade_Marine_group_A_.unclassified.OTU99_0004[illegible]	3.16	Marine	2.89	0.01
Deferribacteres.Deferribacteres.Deferribacterales.SAR406_clade_Marine_group_A_.unclassified.OTU99_0012[illegible]	2.24	Marine	2.48	0.01
Deferribacteres.Deferribacteres.Deferribacterales.SAR406_clade_Marine_group_A_.unclassified.OTU99_0034[illegible]	2.63	Marine	2.49	0.01
Gemmatimonadetes.Gemmatimonadetes.BD2_11_terrestrial_group.unclassified.unclassified.OTU99_000981	2.92	Marine	2.77	0.00
Gemmatimonadetes.Gemmatimonadetes.BD2_11_terrestrial_group.unclassified.unclassified.OTU99_000987	3.06	Marine	2.79	0.01
Gemmatimonadetes.Gemmatimonadetes.BD2_11_terrestrial_group.unclassified.unclassified.OTU99_007545	2.53	Marine	2.44	0.01
Planctomycetes.OM190.unclassified.unclassified.unclassified.OTU99_008413	2.41	Marine	2.42	0.01
Planctomycetes.Phycisphaerae.Phycisphaerales.Phycisphaeraceae.CL500_3.OTU99_001084	2.92	Marine	2.71	0.00
Planctomycetes.Phycisphaerae.Phycisphaerales.Phycisphaeraceae.CL500_3.OTU99_007454	2.42	Marine	2.42	0.01
Planctomycetes.Pla3_lineage	3.25	Marine	2.97	0.00
Planctomycetes.Pla3_lineage.unclassified	3.25	Marine	2.98	0.00
Planctomycetes.Pla3_lineage.unclassified.unclassified	3.25	Marine	2.98	0.00
Planctomycetes.Pla3_lineage.unclassified.unclassified.unclassified	3.25	Marine	2.98	0.00
Planctomycetes.Pla3_lineage.unclassified.unclassified.unclassified.OTU99_000341	2.83	Marine	2.63	0.01
Planctomycetes.Pla3_lineage.unclassified.unclassified.unclassified.OTU99_000658	3.05	Marine	2.77	0.01
Planctomycetes.Planctomycetacia.Planctomycetales.Planctomycetaceae.Blastopirellula	3.60	Marine	3.33	0.03
Planctomycetes.Planctomycetacia.Planctomycetales.Planctomycetaceae.Blastopirellula.OTU99_000438	3.19	Marine	2.93	0.01

Taxonomy	LDA	Envrion-ment	Wilcoxon rank-sum test	Kruskal-Wallis
Planctomycetes.Planctomycetacia.Planctomycetales.Planctomycetaceae.Blastopirellula.OTU99_002524	2.84	Marine	2.64	0.01
Planctomycetes.Planctomycetacia.Planctomycetales.Planctomycetaceae.Blastopirellula.OTU99_003636	2.24	Marine	2.48	0.01
Planctomycetes.Planctomycetacia.Planctomycetales.Planctomycetaceae.Blastopirellula.OTU99_007793	2.62	Marine	2.66	0.01
Planctomycetes.Planctomycetacia.Planctomycetales.Planctomycetaceae.Blastopirellula.OTU99_013319	2.32	Marine	2.77	0.01
Planctomycetes.Planctomycetacia.Planctomycetales.Planctomycetaceae.Planctomyces.OTU99_000627	2.37	Marine	2.76	0.01
Planctomycetes.Planctomycetacia.Planctomycetales.Planctomycetaceae.Planctomyces.OTU99_001136	3.04	Marine	2.83	0.00
Planctomycetes.Planctomycetacia.Planctomycetales.Planctomycetaceae.Planctomyces.OTU99_007960	2.57	Marine	2.58	0.01
Planctomycetes.Planctomycetacia.Planctomycetales.Planctomycetaceae.Planctomyces.OTU99_013028	2.53	Marine	2.44	0.01
Planctomycetes.Planctomycetacia.Planctomycetales.Planctomycetaceae.Rhodopirellula	2.99	Marine	2.80	0.05
Planctomycetes.Planctomycetacia.Planctomycetales.Planctomycetaceae.Rhodopirellula.OTU99_001292	2.88	Marine	2.65	0.01
Planctomycetes.Planctomycetacia.Planctomycetales.Planctomycetaceae.unclassified	3.75	Marine	3.45	0.03
Planctomycetes.Planctomycetacia.Planctomycetales.Planctomycetaceae.unclassified.OTU99_000118	3.64	Marine	3.36	0.00
Planctomycetes.Planctomycetacia.Planctomycetales.Planctomycetaceae.unclassified.OTU99_003028	2.32	Marine	2.74	0.01
Planctomycetes.Planctomycetacia.Planctomycetales.Planctomycetaceae.unclassified.OTU99_008349	2.63	Marine	2.48	0.01
Proteobacteria	5.77	Marine	5.16	0.00
AEGEAN_245.unclassified.unclassified.unclassified.OTU99_001057	3.09	Marine	2.81	0.01
Alphaproteobacteria	5.49	Marine	4.84	0.00
Alphaproteobacteria.Caulobacterales.Hyphomonadaceae.Hyphomonas.OTU99_001348	2.61	Marine	2.77	0.01
Alphaproteobacteria.OCS116_clade	3.69	Marine	3.40	0.00
Alphaproteobacteria.OCS116_clade.unclassified	3.69	Marine	3.40	0.00
Alphaproteobacteria.OCS116_clade.unclassified.unclassified	3.69	Marine	3.40	0.00
Alphaproteobacteria.OCS116_clade.unclassified.unclassified.OTU99_000365	3.44	Marine	3.15	0.00
Alphaproteobacteria.OCS116_clade.unclassified.unclassified.OTU99_000539	3.29	Marine	3.03	0.00
Alphaproteobacteria.Rhizobiales.Rhodobiaceae	3.12	Marine	2.97	0.00
Alphaproteobacteria.Rhizobiales.Rhodobiaceae.Rhodobium	2.98	Marine	2.83	0.00
Alphaproteobacteria.Rhodobacterales	5.08	Marine	4.50	0.00
Alphaproteobacteria.Rhodobacterales.Rhodobacteraceae	5.08	Marine	4.50	0.00
Alphaproteobacteria.Rhodobacterales.Rhodobacteraceae.Candidatus_Planktomarina__DC5_80_3_lineage_	4.50	Marine	4.19	0.00
Alphaproteobacteria.Rhodobacterales.Rhodobacteraceae.Candidatus_Planktomarina__DC5_80_3_lineage_.OT	4.47	Marine	4.17	0.00
Alphaproteobacteria.Rhodobacterales.Rhodobacteraceae.Candidatus_Planktomarina__DC5_80_3_lineage_.OT	2.72	Marine	2.53	0.01
Alphaproteobacteria.Rhodobacterales.Rhodobacteraceae.Candidatus_Planktomarina__DC5_80_3_lineage_.OT	2.53	Marine	2.63	0.01
Alphaproteobacteria.Rhodobacterales.Rhodobacteraceae.Lentibacter	3.44	Marine	3.12	0.03
Alphaproteobacteria.Rhodobacterales.Rhodobacteraceae.Lentibacter.OTU99_000326	3.44	Marine	3.10	0.00
Alphaproteobacteria.Rhodobacterales.Rhodobacteraceae.Octadecabacter	2.60	Marine	2.59	0.01
Alphaproteobacteria.Rhodobacterales.Rhodobacteraceae.Octadecabacter.OTU99_007808	2.60	Marine	2.58	0.01
Alphaproteobacteria.Rhodobacterales.Rhodobacteraceae.Roseobacter_clade_OCT_lineage	4.54	Marine	4.25	0.00
Alphaproteobacteria.Rhodobacterales.Rhodobacteraceae.Roseobacter_clade_OCT_lineage.OTU99_000058	4.43	Marine	4.11	0.00
Alphaproteobacteria.Rhodobacterales.Rhodobacteraceae.Roseobacter_clade_OCT_lineage.OTU99_000091	3.73	Marine	3.45	0.00
Alphaproteobacteria.Rhodobacterales.Rhodobacteraceae.Roseobacter_clade_OCT_lineage.OTU99_000621	3.05	Marine	2.84	0.01
Alphaproteobacteria.Rhodobacterales.Rhodobacteraceae.Roseobacter_clade_OCT_lineage.OTU99_002798	2.81	Marine	2.66	0.01
Alphaproteobacteria.Rhodobacterales.Rhodobacteraceae.Roseobacter_clade_OCT_lineage.OTU99_007475	2.64	Marine	2.50	0.01
Alphaproteobacteria.Rhodobacterales.Rhodobacteraceae.Sulfitobacter	3.54	Marine	3.23	0.00
Alphaproteobacteria.Rhodobacterales.Rhodobacteraceae.Sulfitobacter.OTU99_000335	3.42	Marine	3.11	0.01
Alphaproteobacteria.Rhodobacterales.Rhodobacteraceae.unclassified.OTU99_000033	3.89	Marine	3.62	0.00
Alphaproteobacteria.Rhodobacterales.Rhodobacteraceae.unclassified.OTU99_000304	3.38	Marine	3.10	0.00
Alphaproteobacteria.Rhodobacterales.Rhodobacteraceae.unclassified.OTU99_000324	3.02	Marine	2.80	0.01
Alphaproteobacteria.Rhodobacterales.Rhodobacteraceae.unclassified.OTU99_000411	3.38	Marine	3.08	0.01
Alphaproteobacteria.Rhodobacterales.Rhodobacteraceae.unclassified.OTU99_000601	3.15	Marine	2.88	0.01
Alphaproteobacteria.Rhodobacterales.Rhodobacteraceae.unclassified.OTU99_001661	3.03	Marine	2.87	0.00
Alphaproteobacteria.Rhodobacterales.Rhodobacteraceae.unclassified.OTU99_002324	2.89	Marine	2.75	0.00
Alphaproteobacteria.Rhodobacterales.Rhodobacteraceae.unclassified.OTU99_002819	2.73	Marine	2.68	0.01
Alphaproteobacteria.Rhodobacterales.Rhodobacteraceae.unclassified.OTU99_007665	2.70	Marine	2.72	0.00
Alphaproteobacteria.Rhodobacterales.Rhodobacteraceae.unclassified.OTU99_013224	2.24	Marine	2.49	0.01
Alphaproteobacteria.Rhodobacterales.Rhodobacteraceae.uncultured	4.05	Marine	3.70	0.00
Alphaproteobacteria.Rhodobacterales.Rhodobacteraceae.uncultured.OTU99_002717	2.90	Marine	2.77	0.00
Alphaproteobacteria.Rhodobacterales.Rhodobacteraceae.uncultured.OTU99_013326	2.24	Marine	2.49	0.01
Alphaproteobacteria.Rhodospirillales.Rhodospirillaceae.AEGEAN_169_marine_group	4.20	Marine	3.88	0.00
Alphaproteobacteria.Rhodospirillales.Rhodospirillaceae.AEGEAN_169_marine_group.OTU99_000393	3.02	Marine	2.78	0.01
Alphaproteobacteria.Rhodospirillales.Rhodospirillaceae.AEGEAN_169_marine_group.OTU99_000407	2.78	Marine	2.61	0.01
Alphaproteobacteria.Rhodospirillales.Rhodospirillaceae.AEGEAN_169_marine_group.OTU99_000429	2.24	Marine	2.48	0.01
Alphaproteobacteria.Rhodospirillales.Rhodospirillaceae.AEGEAN_169_marine_group.OTU99_000642	2.96	Marine	2.73	0.01
Alphaproteobacteria.Rhodospirillales.Rhodospirillaceae.AEGEAN_169_marine_group.OTU99_001028	2.73	Marine	2.63	0.00
Alphaproteobacteria.Rhodospirillales.Rhodospirillaceae.AEGEAN_169_marine_group.OTU99_001189	3.07	Marine	2.84	0.01
Alphaproteobacteria.Rhodospirillales.Rhodospirillaceae.AEGEAN_169_marine_group.OTU99_001622	2.84	Marine	2.73	0.00

Taxonomy	LDA	Envrion-ment	Wilcoxon rank-sum test	Kruskal-Wallis
Alphaproteobacteria.Rhodospirillales.Rhodospirillaceae.AEGEAN_169_marine_group.OTU99_001742	2.41	Marine	2.43	0.01
Alphaproteobacteria.Rhodospirillales.Rhodospirillaceae.AEGEAN_169_marine_group.OTU99_001862	2.78	Marine	2.60	0.01
Alphaproteobacteria.Rhodospirillales.Rhodospirillaceae.AEGEAN_169_marine_group.OTU99_004251	2.33	Marine	2.71	0.01
Alphaproteobacteria.Rhodospirillales.Rhodospirillaceae.AEGEAN_169_marine_group.OTU99_006493	2.24	Marine	2.49	0.01
Alphaproteobacteria.Rhodospirillales.Rhodospirillaceae.AEGEAN_169_marine_group.OTU99_007577	2.24	Marine	2.49	0.01
Alphaproteobacteria.Rhodospirillales.Rhodospirillaceae.AEGEAN_169_marine_group.OTU99_008405	2.41	Marine	2.43	0.01
Alphaproteobacteria.Rhodospirillales.Rhodospirillaceae.AEGEAN_169_marine_group.OTU99_013143	2.46	Marine	2.64	0.01
Alphaproteobacteria.Rhodospirillales.Rhodospirillaceae.Defluviicoccus.OTU99_000986	2.92	Marine	2.70	0.01
Alphaproteobacteria.Rhodospirillales.Rhodospirillaceae.Defluviicoccus.OTU99_004623	2.54	Marine	2.46	0.01
Alphaproteobacteria.Rhodospirillales.Rhodospirillaceae.Magnetospira	2.78	Marine	2.57	0.01
Alphaproteobacteria.Rhodospirillales.Rhodospirillaceae.Magnetospira.OTU99_001872	2.54	Marine	2.46	0.01
Alphaproteobacteria.Rhodospirillales.Rhodospirillaceae.OM75_clade	3.38	Marine	3.12	0.00
Alphaproteobacteria.Rhodospirillales.Rhodospirillaceae.OM75_clade.OTU99_000883	3.13	Marine	2.84	0.01
Alphaproteobacteria.Rhodospirillales.Rhodospirillaceae.uncultured.OTU99_001443	2.81	Marine	2.68	0.00
Alphaproteobacteria.Rhodospirillales.unclassified.unclassified.OTU99_002780	2.42	Marine	2.42	0.01
Alphaproteobacteria.Rickettsiales	4.63	Marine	4.20	0.01
Alphaproteobacteria.Rickettsiales.EF100_94H03.unclassified.OTU99_003730	2.70	Marine	2.65	0.01
Alphaproteobacteria.Rickettsiales.S25_593	3.11	Marine	2.85	0.00
Alphaproteobacteria.Rickettsiales.S25_593.unclassified	3.11	Marine	2.84	0.00
Alphaproteobacteria.Rickettsiales.S25_593.unclassified.OTU99_001839	2.83	Marine	2.59	0.01
Alphaproteobacteria.Rickettsiales.S25_593.unclassified.OTU99_002098	2.42	Marine	2.42	0.01
Alphaproteobacteria.Rickettsiales.SAR116_clade	4.58	Marine	4.23	0.00
Alphaproteobacteria.Rickettsiales.SAR116_clade.unclassified	4.58	Marine	4.23	0.00
Alphaproteobacteria.Rickettsiales.SAR116_clade.unclassified.OTU99_000150	4.14	Marine	3.86	0.00
Alphaproteobacteria.Rickettsiales.SAR116_clade.unclassified.OTU99_000168	4.28	Marine	3.99	0.00
Alphaproteobacteria.Rickettsiales.SAR116_clade.unclassified.OTU99_000803	3.14	Marine	2.95	0.00
Alphaproteobacteria.Rickettsiales.SAR116_clade.unclassified.OTU99_001391	2.50	Marine	2.73	0.01
Alphaproteobacteria.Rickettsiales.T9d.unclassified.OTU99_000523	3.10	Marine	2.87	0.01
Alphaproteobacteria.SAR11_clade.Deep_1	3.09	Marine	2.82	0.01
Alphaproteobacteria.SAR11_clade.Deep_1.unclassified	3.09	Marine	2.85	0.01
Alphaproteobacteria.SAR11_clade.Deep_1.unclassified.OTU99_008059	2.41	Marine	2.42	0.01
Alphaproteobacteria.SAR11_clade.Surface_1	4.78	Marine	4.33	0.00
Alphaproteobacteria.SAR11_clade.Surface_1.unclassified	4.78	Marine	4.33	0.00
Alphaproteobacteria.SAR11_clade.Surface_1.unclassified.OTU99_000018	4.13	Marine	3.88	0.00
Alphaproteobacteria.SAR11_clade.Surface_1.unclassified.OTU99_000200	4.20	Marine	3.72	0.04
Alphaproteobacteria.SAR11_clade.Surface_4	3.71	Marine	3.43	0.01
Alphaproteobacteria.SAR11_clade.Surface_4.unclassified	3.71	Marine	3.43	0.01
Alphaproteobacteria.SAR11_clade.Surface_4.unclassified.OTU99_000442	3.17	Marine	2.92	0.00
Alphaproteobacteria.SAR11_clade.unclassified.unclassified.OTU99_000096	3.91	Marine	3.63	0.00
Alphaproteobacteria.SAR11_clade.unclassified.unclassified.OTU99_000578	3.29	Marine	3.01	0.01
Alphaproteobacteria.SAR11_clade.unclassified.unclassified.OTU99_001031	2.94	Marine	2.70	0.01
Alphaproteobacteria.SAR11_clade.unclassified.unclassified.OTU99_001319	3.10	Marine	2.85	0.01
Alphaproteobacteria.SAR11_clade.unclassified.unclassified.OTU99_002056	2.65	Marine	2.45	0.01
Alphaproteobacteria.SAR11_clade.unclassified.unclassified.OTU99_002725	2.64	Marine	2.63	0.01
Alphaproteobacteria.SAR11_clade.unclassified.unclassified.OTU99_003335	2.55	Marine	2.42	0.01
Alphaproteobacteria.SAR11_clade.unclassified.unclassified.OTU99_008490	2.41	Marine	2.43	0.01
Alphaproteobacteria.Sphingomonadales.Erythrobacteraceae.Erythrobacter	2.64	Marine	2.83	0.04
Alphaproteobacteria.Sphingomonadales.Erythrobacteraceae.Erythrobacter.OTU99_024040	2.21	Marine	2.68	0.01
Alphaproteobacteria.unclassified	3.87	Marine	3.35	0.03
Alphaproteobacteria.unclassified.unclassified	3.87	Marine	3.35	0.03
Alphaproteobacteria.unclassified.unclassified.unclassified	3.87	Marine	3.35	0.03
Alphaproteobacteria.unclassified.unclassified.unclassified.OTU99_000465	2.48	Marine	2.92	0.01
Alphaproteobacteria.unclassified.unclassified.unclassified.OTU99_000467	2.82	Marine	2.71	0.00
Alphaproteobacteria.unclassified.unclassified.unclassified.OTU99_001338	3.11	Marine	2.88	0.00
Alphaproteobacteria.unclassified.unclassified.unclassified.OTU99_002473	2.95	Marine	2.68	0.01
Betaproteobacteria.Methylophilales.Methylophilaceae.OM43_clade.OTU99_000092	4.11	Marine	3.82	0.00
Betaproteobacteria.Methylophilales.Methylophilaceae.OM43_clade.OTU99_002663	2.72	Marine	2.55	0.01
Betaproteobacteria.unclassified.unclassified.unclassified.OTU99_007517	2.42	Marine	2.43	0.01
Betaproteobacteria.unclassified.unclassified.unclassified.OTU99_008614	2.67	Marine	2.66	0.00
Deltaproteobacteria.Bdellovibrionales.Bdellovibrionaceae.OM27_clade.OTU99_000769	2.64	Marine	2.79	0.00
Deltaproteobacteria.Desulfobacterales.Nitrospinaceae.Nitrospina.OTU99_000692	2.85	Marine	2.63	0.01
Deltaproteobacteria.Desulfobacterales.Nitrospinaceae.Nitrospina.OTU99_003069	2.63	Marine	2.51	0.01
Deltaproteobacteria.Desulfobacterales.Nitrospinaceae.Nitrospina.OTU99_013276	2.24	Marine	2.49	0.01
Deltaproteobacteria.SAR324_clade_Marine_group_B_.unclassified.unclassified.OTU99_000104	3.65	Marine	3.37	0.01

Taxonomy	LDA	Envrion-ment	Wilcoxon rank-sum test	Kruskal-Wallis
Deltaproteobacteria.SAR324_clade_Marine_group_B_.unclassified.unclassified.OTU99_002101	2.65	Marine	2.45	0.01
Deltaproteobacteria.SAR324_clade_Marine_group_B_.unclassified.unclassified.OTU99_003340	2.55	Marine	2.41	0.01
Deltaproteobacteria.Sh765B_TzT_29.unclassified.unclassified.OTU99_000461	3.24	Marine	2.95	0.01
Deltaproteobacteria.Sh765B_TzT_29.unclassified.unclassified.OTU99_001633	2.71	Marine	2.54	0.01
Gammaproteobacteria	5.27	Marine	4.87	0.00
Gammaproteobacteria.Alteromonadales	4.78	Marine	4.33	0.00
Gammaproteobacteria.Alteromonadales.Alteromonadaceae	4.75	Marine	4.30	0.00
Gammaproteobacteria.Alteromonadales.Alteromonadaceae.BD1_7_clade	2.72	Marine	2.79	0.00
Gammaproteobacteria.Alteromonadales.Alteromonadaceae.Luminiphilus	2.72	Marine	2.60	0.00
Gammaproteobacteria.Alteromonadales.Alteromonadaceae.Luminiphilus.OTU99_008473	2.41	Marine	2.42	0.01
Gammaproteobacteria.Alteromonadales.Alteromonadaceae.Marinobacter	2.21	Marine	2.68	0.01
Gammaproteobacteria.Alteromonadales.Alteromonadaceae.OM60_NOR5__clade	4.30	Marine	3.87	0.04
Gammaproteobacteria.Alteromonadales.Alteromonadaceae.OM60_NOR5__clade.OTU99_000105	4.05	Marine	3.74	0.00
Gammaproteobacteria.Alteromonadales.Alteromonadaceae.OM60_NOR5__clade.OTU99_000374	3.86	Marine	3.58	0.00
Gammaproteobacteria.Alteromonadales.Alteromonadaceae.OM60_NOR5__clade.OTU99_001219	2.49	Marine	2.59	0.01
Gammaproteobacteria.Alteromonadales.Alteromonadaceae.OM60_NOR5__clade.OTU99_001303	2.67	Marine	2.66	0.01
Gammaproteobacteria.Alteromonadales.Alteromonadaceae.SAR92_clade	4.28	Marine	3.86	0.01
Gammaproteobacteria.Alteromonadales.Alteromonadaceae.SAR92_clade.OTU99_000338	3.64	Marine	3.36	0.00
Gammaproteobacteria.Alteromonadales.Alteromonadaceae.SAR92_clade.OTU99_000353	3.57	Marine	3.31	0.00
Gammaproteobacteria.Alteromonadales.Alteromonadaceae.SAR92_clade.OTU99_000457	3.07	Marine	2.82	0.01
Gammaproteobacteria.Alteromonadales.Alteromonadaceae.SAR92_clade.OTU99_000844	3.05	Marine	2.79	0.00
Gammaproteobacteria.Alteromonadales.Alteromonadaceae.SAR92_clade.OTU99_001281	2.93	Marine	2.76	0.01
Gammaproteobacteria.Alteromonadales.Alteromonadaceae.unclassified	4.12	Marine	3.80	0.00
Gammaproteobacteria.Alteromonadales.Alteromonadaceae.unclassified.OTU99_000131	4.05	Marine	3.73	0.00
Gammaproteobacteria.Alteromonadales.Alteromonadaceae.unclassified.OTU99_000347	2.67	Marine	2.65	0.00
Gammaproteobacteria.Alteromonadales.Alteromonadaceae.unclassified.OTU99_013155	2.32	Marine	2.76	0.01
Gammaproteobacteria.Alteromonadales.Psychromonadaceae	2.64	Marine	2.49	0.01
Gammaproteobacteria.Alteromonadales.Psychromonadaceae.Psychromonas	2.64	Marine	2.49	0.01
Gammaproteobacteria.Chromatiales.unclassified.unclassified.OTU99_002020	2.32	Marine	2.75	0.01
Gammaproteobacteria.Chromatiales.unclassified.unclassified.OTU99_002833	2.52	Marine	2.67	0.01
Gammaproteobacteria.E01_9C_26_marine_group	2.71	Marine	2.52	0.01
Gammaproteobacteria.E01_9C_26_marine_group.unclassified	2.71	Marine	2.53	0.01
Gammaproteobacteria.E01_9C_26_marine_group.unclassified.unclassified	2.71	Marine	2.53	0.01
Gammaproteobacteria.E01_9C_26_marine_group.unclassified.unclassified.OTU99_002772	2.53	Marine	2.44	0.01
Gammaproteobacteria.Enterobacteriales.Enterobacteriaceae.Escherichia_Shigella	2.41	Marine	2.42	0.01
Gammaproteobacteria.Enterobacteriales.Enterobacteriaceae.Escherichia_Shigella.OTU99_001104	2.41	Marine	2.43	0.01
Gammaproteobacteria.Gammaproteobacteria_Incertae_Sedis	2.46	Marine	2.64	0.01
Gammaproteobacteria.Gammaproteobacteria_Incertae_Sedis.Unknown_Family	2.46	Marine	2.62	0.01
Gammaproteobacteria.Gammaproteobacteria_Incertae_Sedis.Unknown_Family.Arenicella	2.46	Marine	2.63	0.01
Gammaproteobacteria.KI89A_clade	3.52	Marine	3.24	0.00
Gammaproteobacteria.KI89A_clade.unclassified	3.52	Marine	3.24	0.00
Gammaproteobacteria.KI89A_clade.unclassified.unclassified	3.52	Marine	3.24	0.00
Gammaproteobacteria.KI89A_clade.unclassified.unclassified.OTU99_000889	3.29	Marine	3.02	0.00
Gammaproteobacteria.KI89A_clade.unclassified.unclassified.OTU99_001763	2.77	Marine	2.88	0.01
Gammaproteobacteria.Oceanospirillales	4.96	Marine	4.63	0.00
Gammaproteobacteria.Oceanospirillales.Alcanivoracaceae.Alcanivorax.OTU99_005000	2.19	Marine	3.09	0.01
Gammaproteobacteria.Oceanospirillales.Litoricolaceae	3.45	Marine	3.19	0.00
Gammaproteobacteria.Oceanospirillales.Litoricolaceae.Litoricola	3.45	Marine	3.19	0.00
Gammaproteobacteria.Oceanospirillales.Litoricolaceae.Litoricola.OTU99_000846	3.45	Marine	3.19	0.00
Gammaproteobacteria.Oceanospirillales.Oceanospirillaceae.Pseudospirillum.OTU99_000348	3.32	Marine	3.04	0.01
Gammaproteobacteria.Oceanospirillales.Oceanospirillaceae.Pseudospirillum.OTU99_001067	3.13	Marine	2.92	0.01
Gammaproteobacteria.Oceanospirillales.Oceanospirillaceae.Pseudospirillum.OTU99_004575	2.54	Marine	2.46	0.01
Gammaproteobacteria.Oceanospirillales.OM182_clade.unclassified.OTU99_000960	2.63	Marine	2.47	0.01
Gammaproteobacteria.Oceanospirillales.OM182_clade.unclassified.OTU99_002811	2.55	Marine	2.42	0.01
Gammaproteobacteria.Oceanospirillales.SAR86_clade	4.72	Marine	4.44	0.00
Gammaproteobacteria.Oceanospirillales.SAR86_clade.unclassified	4.72	Marine	4.44	0.00
Gammaproteobacteria.Oceanospirillales.SAR86_clade.unclassified.OTU99_000042	4.49	Marine	4.21	0.00
Gammaproteobacteria.Oceanospirillales.SAR86_clade.unclassified.OTU99_000189	4.02	Marine	3.73	0.00
Gammaproteobacteria.Oceanospirillales.SAR86_clade.unclassified.OTU99_000371	3.61	Marine	3.34	0.01
Gammaproteobacteria.Oceanospirillales.SAR86_clade.unclassified.OTU99_000581	3.31	Marine	3.04	0.00
Gammaproteobacteria.Oceanospirillales.SAR86_clade.unclassified.OTU99_000958	2.26	Marine	2.70	0.01
Gammaproteobacteria.Oceanospirillales.SAR86_clade.unclassified.OTU99_001103	3.15	Marine	2.94	0.00
Gammaproteobacteria.Oceanospirillales.SAR86_clade.unclassified.OTU99_001234	2.79	Marine	2.56	0.01
Gammaproteobacteria.Oceanospirillales.SAR86_clade.unclassified.OTU99_002038	3.12	Marine	2.93	0.00

Taxonomy	LDA	Envrion-ment	Wilcoxon rank-sum test	Kruskal-Wallis
Gammaproteobacteria.Oceanospirillales.SAR86_clade.unclassified.OTU99_005715	2.51	Marine	2.63	0.01
Gammaproteobacteria.Oceanospirillales.unclassified.unclassified.OTU99_000023	4.32	Marine	4.04	0.01
Gammaproteobacteria.Oceanospirillales.unclassified.unclassified.OTU99_002743	2.90	Marine	2.64	0.01
Gammaproteobacteria.Order_Incertae_Sedis	3.30	Marine	3.02	0.03
Gammaproteobacteria.Order_Incertae_Sedis.Family_Incertae_Sedis	3.30	Marine	3.02	0.03
Gammaproteobacteria.Order_Incertae_Sedis.Family_Incertae_Sedis.Marinicella	3.30	Marine	3.02	0.03
Gammaproteobacteria.Order_Incertae_Sedis.Family_Incertae_Sedis.Marinicella.OTU99_001840	2.96	Marine	2.78	0.00
Gammaproteobacteria.Order_Incertae_Sedis.Family_Incertae_Sedis.Marinicella.OTU99_003597	2.67	Marine	2.68	0.00
Gammaproteobacteria.Pseudomonadales.Moraxellaceae.Psychrobacter	3.23	Marine	2.77	0.03
Gammaproteobacteria.Pseudomonadales.Moraxellaceae.Psychrobacter.OTU99_000963	2.48	Marine	3.14	0.02
Gammaproteobacteria.Pseudomonadales.Moraxellaceae.Psychrobacter.OTU99_004056	2.65	Marine	2.68	0.01
Gammaproteobacteria.Salinisphaerales	2.32	Marine	2.76	0.01
Gammaproteobacteria.Salinisphaerales.Salinisphaeraceae	2.32	Marine	2.75	0.01
Gammaproteobacteria.Salinisphaerales.Salinisphaeraceae.ZD0417_marine_group	2.32	Marine	2.76	0.01
Gammaproteobacteria.Salinisphaerales.Salinisphaeraceae.ZD0417_marine_group.OTU99_007876	2.32	Marine	2.75	0.01
Gammaproteobacteria.Sva0071	2.53	Marine	2.65	0.01
Gammaproteobacteria.Sva0071.unclassified	2.53	Marine	2.63	0.01
Gammaproteobacteria.Sva0071.unclassified.unclassified	2.53	Marine	2.63	0.01
Gammaproteobacteria.Thiotrichales.Piscirickettsiaceae.unclassified.OTU99_002651	2.85	Marine	2.77	0.01
Gammaproteobacteria.Thiotrichales.Thiotrichaceae	2.99	Marine	2.83	0.00
Gammaproteobacteria.Thiotrichales.Thiotrichaceae.Thiothrix	2.99	Marine	2.88	0.00
Gammaproteobacteria.Thiotrichales.Thiotrichaceae.Thiothrix.OTU99_000471	2.59	Marine	2.85	0.00
Gammaproteobacteria.Thiotrichales.Thiotrichaceae.Thiothrix.OTU99_010606	2.32	Marine	2.76	0.01
Gammaproteobacteria.Thiotrichales.Thiotrichaceae.Thiothrix.OTU99_013351	2.32	Marine	2.75	0.01
Gammaproteobacteria.unclassified	4.25	Marine	3.71	0.00
Gammaproteobacteria.unclassified.unclassified	4.25	Marine	3.71	0.00
Gammaproteobacteria.unclassified.unclassified.unclassified	4.25	Marine	3.71	0.00
Gammaproteobacteria.unclassified.unclassified.unclassified.OTU99_000240	3.01	Marine	2.78	0.01
Gammaproteobacteria.unclassified.unclassified.unclassified.OTU99_000905	2.97	Marine	2.82	0.01
Gammaproteobacteria.unclassified.unclassified.unclassified.OTU99_001525	2.50	Marine	2.72	0.01
Gammaproteobacteria.unclassified.unclassified.unclassified.OTU99_001740	2.52	Marine	2.51	0.01
Gammaproteobacteria.unclassified.unclassified.unclassified.OTU99_002661	3.02	Marine	2.84	0.00
Gammaproteobacteria.unclassified.unclassified.unclassified.OTU99_003458	2.32	Marine	2.76	0.01
Gammaproteobacteria.unclassified.unclassified.unclassified.OTU99_007063	2.42	Marine	2.43	0.01
Gammaproteobacteria.unclassified.unclassified.unclassified.OTU99_013133	2.24	Marine	2.50	0.01
Gammaproteobacteria.Vibrionales	2.83	Marine	2.84	0.00
Gammaproteobacteria.Vibrionales.Vibrionaceae	2.83	Marine	2.84	0.00
Gammaproteobacteria.Vibrionales.Vibrionaceae.Vibrio	2.47	Marine	2.88	0.01
Gammaproteobacteria.Vibrionales.Vibrionaceae.Vibrio.OTU99_005792	2.31	Marine	2.98	0.01
Gammaproteobacteria.Xanthomonadales.JTB255_marine_benthic_group.unclassified.OTU99_004586	2.32	Marine	2.74	0.01
SC3_20.unclassified.unclassified.unclassified.OTU99_001722	2.78	Marine	2.95	0.03
SPOTSOCT00m83.unclassified.unclassified.unclassified.OTU99_001842	2.71	Marine	2.54	0.01
unclassified	4.60	Marine	4.02	0.04
unclassified.unclassified	4.60	Marine	4.02	0.04
unclassified.unclassified.unclassified	4.60	Marine	4.02	0.04
unclassified.unclassified.unclassified.unclassified	4.60	Marine	4.02	0.04
unclassified.unclassified.unclassified.unclassified.OTU99_000072	4.06	Marine	3.78	0.00
unclassified.unclassified.unclassified.unclassified.OTU99_000207	3.36	Marine	3.11	0.00
unclassified.unclassified.unclassified.unclassified.OTU99_000307	3.14	Marine	2.90	0.01
unclassified.unclassified.unclassified.unclassified.OTU99_000529	3.37	Marine	3.14	0.00
unclassified.unclassified.unclassified.unclassified.OTU99_000583	3.61	Marine	3.28	0.00
unclassified.unclassified.unclassified.unclassified.OTU99_001648	2.79	Marine	2.59	0.01
unclassified.unclassified.unclassified.unclassified.OTU99_002641	2.63	Marine	2.52	0.01
unclassified.unclassified.unclassified.unclassified.OTU99_002779	2.55	Marine	2.41	0.01
unclassified.unclassified.unclassified.unclassified.OTU99_003293	2.62	Marine	2.66	0.01
unclassified.unclassified.unclassified.unclassified.OTU99_003518	2.54	Marine	2.46	0.01
unclassified.unclassified.unclassified.unclassified.OTU99_003854	2.90	Marine	2.77	0.00
unclassified.unclassified.unclassified.unclassified.OTU99_007619	2.42	Marine	2.42	0.01
unclassified.unclassified.unclassified.unclassified.OTU99_013246	2.24	Marine	2.48	0.01
unclassified.unclassified.unclassified.unclassified.OTU99_013513	2.33	Marine	2.71	0.01
Verrucomicrobia.Arctic97B_4_marine_group	2.54	Marine	2.46	0.01
Verrucomicrobia.Arctic97B_4_marine_group.unclassified	2.54	Marine	2.46	0.01
Verrucomicrobia.Arctic97B_4_marine_group.unclassified.unclassified	2.54	Marine	2.47	0.01
Verrucomicrobia.Arctic97B_4_marine_group.unclassified.unclassified.unclassified	2.54	Marine	2.46	0.01

Taxonomy	LDA	Envrion-ment	Wilcoxon rank-sum test	Kruskal-Wallis
Verrucomicrobia.Arctic97B_4_marine_group.unclassified.unclassified.unclassified.OTU99_003611	2.24	Marine	2.49	0.01
Verrucomicrobia.Opitutae.MB11C04_marine_group	3.69	Marine	3.40	0.00
Verrucomicrobia.Opitutae.MB11C04_marine_group.unclassified	3.69	Marine	3.40	0.00
Verrucomicrobia.Opitutae.MB11C04_marine_group.unclassified.unclassified	3.69	Marine	3.39	0.00
Verrucomicrobia.Opitutae.MB11C04_marine_group.unclassified.unclassified.OTU99_000869	2.99	Marine	2.79	0.00
Verrucomicrobia.Opitutae.MB11C04_marine_group.unclassified.unclassified.OTU99_001109	2.99	Marine	2.78	0.00
Verrucomicrobia.Opitutae.MB11C04_marine_group.unclassified.unclassified.OTU99_001788	2.88	Marine	2.83	0.01
Verrucomicrobia.Opitutae.MB11C04_marine_group.unclassified.unclassified.OTU99_003575	2.69	Marine	2.84	0.01
Verrucomicrobia.Opitutae.Puniceicoccales.Puniceicoccaceae.Lentimonas	3.29	Marine	3.00	0.00
Verrucomicrobia.Opitutae.Puniceicoccales.Puniceicoccaceae.Lentimonas.OTU99_001863	3.17	Marine	2.93	0.00
Verrucomicrobia.Opitutae.Puniceicoccales.Puniceicoccaceae.unclassified.OTU99_000659	2.77	Marine	2.83	0.01
Verrucomicrobia.Verrucomicrobiae.Verrucomicrobiales.Verrucomicrobiaceae.Persicirhabdus	3.17	Marine	2.88	0.00
Verrucomicrobia.Verrucomicrobiae.Verrucomicrobiales.Verrucomicrobiaceae.Roseibacillus.OTU99_000146	3.18	Marine	2.94	0.00
Verrucomicrobia.Verrucomicrobiae.Verrucomicrobiales.Verrucomicrobiaceae.Roseibacillus.OTU99_000170	3.05	Marine	2.79	0.00
Verrucomicrobia.Verrucomicrobiae.Verrucomicrobiales.Verrucomicrobiaceae.Roseibacillus.OTU99_000942	3.24	Marine	2.93	0.00
Acidobacteria.Acidobacteria.Subgroup_3	3.06	Brack	2.89	0.04
Acidobacteria.Acidobacteria.Subgroup_3.PAUC26f	3.06	Brack	2.89	0.04
Acidobacteria.Acidobacteria.Subgroup_3.PAUC26f.unclassified	3.06	Brack	2.87	0.04
Actinobacteria	5.21	Brack	4.70	0.00
Actinobacteria.Acidimicrobiia.Acidimicrobiales.Acidimicrobiaceae	4.36	Brack	3.93	0.01
Actinobacteria.Acidimicrobiia.Acidimicrobiales.Acidimicrobiaceae.CL500_29_marine_group	4.21	Brack	3.88	0.00
Actinobacteria.Acidimicrobiia.Acidimicrobiales.Acidimicrobiaceae.CL500_29_marine_group.OTU99_000009	3.76	Brack	3.43	0.01
Actinobacteria.Acidimicrobiia.Acidimicrobiales.Acidimicrobiaceae.CL500_29_marine_group.OTU99_000450	3.42	Brack	3.17	0.00
Actinobacteria.Acidimicrobiia.Acidimicrobiales.Acidimicrobiaceae.unclassified	3.04	Brack	2.89	0.02
Actinobacteria.Acidimicrobiia.Acidimicrobiales.uncultured	3.96	Brack	3.62	0.00
Actinobacteria.Acidimicrobiia.Acidimicrobiales.uncultured.unclassified	3.96	Brack	3.61	0.00
Actinobacteria.Actinobacteria	5.07	Brack	4.63	0.00
Actinobacteria.Actinobacteria.Corynebacteriales	4.34	Brack	4.02	0.00
Actinobacteria.Actinobacteria.Corynebacteriales.Mycobacteriaceae	3.50	Brack	3.28	0.00
Actinobacteria.Actinobacteria.Corynebacteriales.Mycobacteriaceae.Mycobacterium	3.50	Brack	3.28	0.00
Actinobacteria.Actinobacteria.Corynebacteriales.Mycobacteriaceae.Mycobacterium.OTU99_000216	3.27	Brack	3.06	0.04
Actinobacteria.Actinobacteria.Corynebacteriales.unclassified	4.27	Brack	3.94	0.00
Actinobacteria.Actinobacteria.Corynebacteriales.unclassified.unclassified	4.27	Brack	3.95	0.00
Actinobacteria.Actinobacteria.Corynebacteriales.unclassified.unclassified.OTU99_000035	4.09	Brack	3.77	0.04
Actinobacteria.Actinobacteria.Frankiales	4.77	Brack	4.46	0.00
Actinobacteria.Actinobacteria.Frankiales.Sporichthyaceae	4.77	Brack	4.46	0.00
Actinobacteria.Actinobacteria.Frankiales.Sporichthyaceae.hgcI_clade	4.75	Brack	4.44	0.00
Actinobacteria.Actinobacteria.Frankiales.Sporichthyaceae.hgcI_clade.OTU99_000021	4.04	Brack	3.75	0.01
Actinobacteria.Actinobacteria.Frankiales.Sporichthyaceae.hgcI_clade.OTU99_000036	4.30	Brack	3.99	0.00
Actinobacteria.Actinobacteria.Frankiales.Sporichthyaceae.hgcI_clade.OTU99_000045	3.97	Brack	3.65	0.00
Actinobacteria.Actinobacteria.Frankiales.Sporichthyaceae.hgcI_clade.OTU99_000147	3.64	Brack	3.32	0.02
Actinobacteria.Actinobacteria.Frankiales.Sporichthyaceae.hgcI_clade.OTU99_000354	3.40	Brack	3.12	0.00
Actinobacteria.Actinobacteria.Frankiales.Sporichthyaceae.unclassified	3.36	Brack	3.10	0.01
Actinobacteria.Actinobacteria.Frankiales.Sporichthyaceae.unclassified.OTU99_000220	3.22	Brack	2.92	0.04
Actinobacteria.Thermoleophilia	3.33	Brack	3.05	0.02
Actinobacteria.Thermoleophilia.Solirubrobacterales	3.19	Brack	2.93	0.03
Bacteroidetes.Cytophagia.Cytophagales.Cyclobacteriaceae	3.83	Brack	3.47	0.01
Bacteroidetes.Cytophagia.Cytophagales.Cyclobacteriaceae.unclassified	3.69	Brack	3.32	0.01
Bacteroidetes.Cytophagia.Cytophagales.Cyclobacteriaceae.unclassified.OTU99_000039	3.66	Brack	3.29	0.02
Bacteroidetes.Cytophagia.Cytophagales.Cytophagaceae.Leadbetterella	3.06	Brack	3.01	0.04
Bacteroidetes.Cytophagia.Order_II	3.03	Brack	2.91	0.03
Bacteroidetes.Cytophagia.Order_II.Rhodothermaceae	3.03	Brack	2.93	0.03
Bacteroidetes.Cytophagia.Order_II.Rhodothermaceae.uncultured	3.03	Brack	2.92	0.03
Bacteroidetes.Flavobacteriia.Flavobacteriales.Cryomorphaceae.Fluviicola.OTU99_000202	3.40	Brack	3.12	0.02
Bacteroidetes.Flavobacteriia.Flavobacteriales.Cryomorphaceae.Owenweeksia	4.04	Brack	3.57	0.00
Bacteroidetes.Flavobacteriia.Flavobacteriales.Cryomorphaceae.Owenweeksia.OTU99_000145	3.68	Brack	3.40	0.01
Bacteroidetes.Flavobacteriia.Flavobacteriales.Cryomorphaceae.Owenweeksia.OTU99_000178	3.38	Brack	3.05	0.03
Bacteroidetes.Flavobacteriia.Flavobacteriales.Flavobacteriaceae.Flavobacterium.OTU99_000253	3.37	Brack	3.19	0.03
Bacteroidetes.Flavobacteriia.Flavobacteriales.Flavobacteriaceae.unclassified.OTU99_000031	4.07	Brack	3.58	0.02
Bacteroidetes.Flavobacteriia.Flavobacteriales.Flavobacteriaceae.unclassified.OTU99_000062	3.91	Brack	3.62	0.00
Bacteroidetes.Flavobacteriia.Flavobacteriales.Flavobacteriaceae.unclassified.OTU99_000069	3.85	Brack	3.51	0.05
Bacteroidetes.Flavobacteriia.Flavobacteriales.Flavobacteriaceae.unclassified.OTU99_000103	2.79	Brack	2.81	0.04
Bacteroidetes.Flavobacteriia.Flavobacteriales.NS9_marine_group	4.04	Brack	3.59	0.02
Bacteroidetes.Flavobacteriia.Flavobacteriales.NS9_marine_group.unclassified	4.04	Brack	3.59	0.02

Taxonomy	LDA	Envrion-ment	Wilcoxon rank-sum test	Kruskal-Wallis
Bacteroidetes.Flavobacteriia.Flavobacteriales.NS9_marine_group.unclassified.OTU99_000051	3.68	Brack	3.40	0.01
Bacteroidetes.Sphingobacteriia.Sphingobacteriales.NS11_12_marine_group	4.07	Brack	3.70	0.00
Bacteroidetes.Sphingobacteriia.Sphingobacteriales.NS11_12_marine_group.unclassified	4.07	Brack	3.70	0.00
Bacteroidetes.Sphingobacteriia.Sphingobacteriales.NS11_12_marine_group.unclassified.OTU99_000054	3.84	Brack	3.52	0.00
Bacteroidetes.Sphingobacteriia.Sphingobacteriales.Sphingobacteriaceae	3.10	Brack	2.92	0.00
Bacteroidetes.Sphingobacteriia.Sphingobacteriales.Sphingobacteriaceae.Pedobacter	3.06	Brack	2.91	0.00
Bacteroidetes.Sphingobacteriia.Sphingobacteriales.unclassified	3.17	Brack	2.95	0.03
Bacteroidetes.Sphingobacteriia.Sphingobacteriales.unclassified.unclassified	3.17	Brack	2.94	0.03
bacteroidetes.unclassified.unclassified.unclassified.unclassified.OTU99_000025	3.39	Brack	3.09	0.04
bacteroidetes.unclassified.unclassified.unclassified.unclassified.OTU99_000132	3.53	Brack	3.25	0.03
Chloroflexi.Caldilineae	3.64	Brack	3.40	0.01
Chloroflexi.Caldilineae.Caldilineales	3.64	Brack	3.40	0.01
Chloroflexi.Caldilineae.Caldilineales.Caldilineaceae	3.64	Brack	3.40	0.01
Chloroflexi.Caldilineae.Caldilineales.Caldilineaceae.uncultured	3.64	Brack	3.40	0.01
Chloroflexi.Caldilineae.Caldilineales.Caldilineaceae.uncultured.OTU99_000079	3.55	Brack	3.32	0.04
Chloroflexi.unclassified	2.82	Brack	2.95	0.02
Chloroflexi.unclassified.unclassified	2.82	Brack	2.96	0.02
Chloroflexi.unclassified.unclassified.unclassified	2.82	Brack	2.95	0.02
Chloroflexi.unclassified.unclassified.unclassified.unclassified	2.82	Brack	2.96	0.02
Chloroflexi.unclassified.unclassified.unclassified.unclassified.OTU99_001117	2.53	Brack	2.90	0.04
Cyanobacteria	5.08	Brack	4.47	0.03
Cyanobacteria.Cyanobacteria	5.07	Brack	4.47	0.04
Cyanobacteria.Cyanobacteria.SubsectionI.FamilyI.Synechococcus.OTU99_000013	4.66	Brack	4.29	0.00
Cyanobacteria.Cyanobacteria.SubsectionI.FamilyI.Synechococcus.OTU99_000057	4.13	Brack	3.67	0.03
Cyanobacteria.Cyanobacteria.SubsectionIV	4.53	Brack	4.12	0.00
Cyanobacteria.Cyanobacteria.SubsectionIV.FamilyI	4.53	Brack	4.12	0.00
Cyanobacteria.Cyanobacteria.SubsectionIV.FamilyI.unclassified	4.52	Brack	4.11	0.00
Cyanobacteria.Cyanobacteria.SubsectionIV.FamilyI.unclassified.OTU99_000064	4.12	Brack	3.84	0.00
Lentisphaerae.WCHB1_41	3.26	Brack	3.01	0.03
Lentisphaerae.WCHB1_41.unclassified	3.26	Brack	3.00	0.03
Lentisphaerae.WCHB1_41.unclassified.unclassified	3.26	Brack	2.99	0.03
Lentisphaerae.WCHB1_41.unclassified.unclassified.unclassified	3.26	Brack	3.00	0.03
Planctomycetes	4.72	Brack	4.19	0.03
Planctomycetes.Planctomycetacia	4.63	Brack	4.14	0.01
Planctomycetes.Planctomycetacia.Planctomycetales	4.63	Brack	4.14	0.01
Planctomycetes.Planctomycetacia.Planctomycetales.Planctomycetaceae	4.63	Brack	4.14	0.01
Planctomycetes.Planctomycetacia.Planctomycetales.Planctomycetaceae.uncultured	4.46	Brack	4.18	0.00
Planctomycetes.Planctomycetacia.Planctomycetales.Planctomycetaceae.uncultured.OTU99_000134	3.75	Brack	3.49	0.01
Planctomycetes.Planctomycetacia.Planctomycetales.Planctomycetaceae.uncultured.OTU99_000138	3.41	Brack	3.17	0.01
Planctomycetes.Planctomycetacia.Planctomycetales.Planctomycetaceae.uncultured.OTU99_000959	3.02	Brack	2.88	0.04
Alphaproteobacteria.Rhizobiales.unclassified	2.98	Brack	2.93	0.01
Alphaproteobacteria.Rhizobiales.unclassified.unclassified	2.98	Brack	2.93	0.01
Alphaproteobacteria.Rhodobacterales.Rhodobacteraceae.Loktanella	3.73	Brack	3.35	0.01
Alphaproteobacteria.Rhodobacterales.Rhodobacteraceae.Loktanella.OTU99_000116	3.69	Brack	3.31	0.04
Alphaproteobacteria.Rhodobacterales.Rhodobacteraceae.unclassified	4.69	Brack	3.88	0.05
Alphaproteobacteria.Rhodobacterales.Rhodobacteraceae.unclassified.OTU99_000016	4.32	Brack	3.93	0.04
Alphaproteobacteria.Rhodobacterales.Rhodobacteraceae.unclassified.OTU99_000136	3.86	Brack	3.50	0.03
Alphaproteobacteria.Rhodospirillales.Rhodospirillaceae.Thalassobaculum	3.13	Brack	2.99	0.00
Alphaproteobacteria.Rhodospirillales.Rhodospirillaceae.Thalassobaculum.OTU99_000879	2.93	Brack	2.91	0.01
Alphaproteobacteria.Rhodospirillales.Rhodospirillaceae.Thalassospira.OTU99_000625	2.87	Brack	2.85	0.04
Alphaproteobacteria.Rhodospirillales.Rhodospirillaceae.uncultured.OTU99_000417	2.69	Brack	3.19	0.02
Alphaproteobacteria.Rhodospirillales.Rhodospirillales_Incertae_Sedis	3.52	Brack	3.27	0.01
Alphaproteobacteria.Rhodospirillales.Rhodospirillales_Incertae_Sedis.Reyranella	3.52	Brack	3.27	0.01
Alphaproteobacteria.Rickettsiales.mitochondria	2.76	Brack	2.99	0.04
Alphaproteobacteria.Rickettsiales.mitochondria.unclassified	2.76	Brack	2.99	0.04
Alphaproteobacteria.Rickettsiales.Rickettsiaceae	3.11	Brack	2.99	0.00
Alphaproteobacteria.Rickettsiales.Rickettsiaceae.Rickettsia	3.07	Brack	3.01	0.00
Alphaproteobacteria.Rickettsiales.Rickettsiales_Incertae_Sedis	3.34	Brack	3.03	0.02
Alphaproteobacteria.Rickettsiales.Rickettsiales_Incertae_Sedis.Candidatus_Captivus	2.80	Brack	3.05	0.04
Alphaproteobacteria.SAR11_clade.Chesapeake_Delaware_Bay	4.62	Brack	4.23	0.00
Alphaproteobacteria.SAR11_clade.Chesapeake_Delaware_Bay.unclassified	4.62	Brack	4.23	0.00
Alphaproteobacteria.SAR11_clade.Chesapeake_Delaware_Bay.unclassified.OTU99_000014	4.61	Brack	4.24	0.00
Alphaproteobacteria.Sphingomonadales.Sphingomonadaceae	3.62	Brack	3.35	0.00
Alphaproteobacteria.Sphingomonadales.Sphingomonadaceae.unclassified	3.58	Brack	3.32	0.00

Taxonomy	LDA	Envrion-ment	Wilcoxon rank-sum test	Kruskal-Wallis
Alphaproteobacteria.Sphingomonadales.Sphingomonadaceae.unclassified.OTU99_000316	3.47	Brack	3.21	0.01
Alphaproteobacteria.unclassified.unclassified.unclassified.OTU99_001087	2.89	Brack	3.00	0.03
Betaproteobacteria	4.74	Brack	4.13	0.02
Betaproteobacteria.Burkholderiales	4.53	Brack	4.13	0.00
Betaproteobacteria.Burkholderiales.Alcaligenaceae	4.13	Brack	3.76	0.00
Betaproteobacteria.Burkholderiales.Alcaligenaceae.MWH_UniP1_aquatic_group	3.70	Brack	3.26	0.03
Betaproteobacteria.Burkholderiales.Alcaligenaceae.unclassified	3.89	Brack	3.57	0.00
Betaproteobacteria.Burkholderiales.Alcaligenaceae.unclassified.OTU99_000545	3.30	Brack	3.05	0.03
Betaproteobacteria.Burkholderiales.Comamonadaceae	4.21	Brack	3.76	0.00
Betaproteobacteria.Burkholderiales.Comamonadaceae.Limnohabitans	3.46	Brack	3.19	0.01
Betaproteobacteria.Burkholderiales.Comamonadaceae.unclassified	3.47	Brack	3.20	0.00
Betaproteobacteria.Burkholderiales.unclassified	3.54	Brack	3.29	0.04
Betaproteobacteria.Burkholderiales.unclassified.unclassified	3.54	Brack	3.29	0.04
Betaproteobacteria.TRA3_20	2.84	Brack	2.88	0.02
Betaproteobacteria.TRA3_20.unclassified	2.84	Brack	2.87	0.02
Betaproteobacteria.TRA3_20.unclassified.unclassified	2.84	Brack	2.89	0.02
Betaproteobacteria.unclassified	3.93	Brack	3.54	0.02
Betaproteobacteria.unclassified.unclassified	3.93	Brack	3.54	0.02
Betaproteobacteria.unclassified.unclassified.unclassified	3.93	Brack	3.54	0.02
unclassified.unclassified.unclassified.unclassified.OTU99_000019	4.15	Brack	3.50	0.04
unclassified	4.53	Brack	4.19	0.00
unclassified.unclassified	4.53	Brack	4.19	0.00
unclassified.unclassified.unclassified	4.53	Brack	4.19	0.00
unclassified.unclassified.unclassified.unclassified	4.53	Brack	4.19	0.00
unclassified.unclassified.unclassified.unclassified.unclassified	4.53	Brack	4.19	0.00
unclassified.unclassified.unclassified.unclassified.unclassified.OTU99_000029	4.02	Brack	3.72	0.01
unclassified.unclassified.unclassified.unclassified.unclassified.OTU99_000061	3.99	Brack	3.64	0.01
unclassified.unclassified.unclassified.unclassified.unclassified.OTU99_000223	3.41	Brack	3.12	0.03
unclassified.unclassified.unclassified.unclassified.unclassified.OTU99_000646	3.03	Brack	2.94	0.03
Verrucomicrobia	5.13	Brack	4.70	0.00
Verrucomicrobia.Opitutae.Opitutales	3.17	Brack	3.02	0.01
Verrucomicrobia.Opitutae.Opitutales.Opitutaceae	3.17	Brack	3.02	0.01
Verrucomicrobia.Opitutae.Opitutales.Opitutaceae.Opitutus	3.02	Brack	2.97	0.00
Verrucomicrobia.Spartobacteria	5.05	Brack	4.72	0.00
Verrucomicrobia.Spartobacteria.Chthoniobacterales	5.05	Brack	4.72	0.00
Verrucomicrobia.Spartobacteria.Chthoniobacterales.FukuN18_freshwater_group	3.75	Brack	3.44	0.00
Verrucomicrobia.Spartobacteria.Chthoniobacterales.FukuN18_freshwater_group.unclassified	3.75	Brack	3.44	0.00
Verrucomicrobia.Spartobacteria.Chthoniobacterales.FukuN18_freshwater_group.unclassified.OTU99_000117	3.52	Brack	3.23	0.01
Verrucomicrobia.Spartobacteria.Chthoniobacterales.FukuN18_freshwater_group.unclassified.OTU99_000406	3.15	Brack	3.18	0.01
Verrucomicrobia.Spartobacteria.Chthoniobacterales.unclassified	5.02	Brack	4.69	0.00
Verrucomicrobia.Spartobacteria.Chthoniobacterales.unclassified.unclassified	5.02	Brack	4.69	0.00
Verrucomicrobia.Spartobacteria.Chthoniobacterales.unclassified.unclassified.OTU99_000005	4.96	Brack	4.63	0.00
Verrucomicrobia.Verrucomicrobia_Incertae_Sedis	3.56	Brack	3.30	0.00
Verrucomicrobia.Verrucomicrobia_Incertae_Sedis.Unknown_Order	3.56	Brack	3.30	0.00
Verrucomicrobia.Verrucomicrobia_Incertae_Sedis.Unknown_Order.Unknown_Family	3.56	Brack	3.30	0.00
Verrucomicrobia.Verrucomicrobia_Incertae_Sedis.Unknown_Order.Unknown_Family.Candidatus_Methylacidiph	3.56	Brack	3.30	0.00
Verrucomicrobia.Verrucomicrobia_Incertae_Sedis.Unknown_Order.Unknown_Family.Candidatus_Methylacidiph	3.49	Brack	3.24	0.00
Verrucomicrobia.Verrucomicrobiae.Verrucomicrobiales.unclassified.unclassified.OTU99_000283	3.08	Brack	2.96	0.04

Taxonomy	LDA	Envrion-ment	Wilcoxon rank-sum test	Kruskal-Wallis
Acidobacteria	3.70	Winter	3.48	0.00
Acidobacteria.Acidobacteria	3.70	Winter	3.48	0.00
Acidobacteria.Acidobacteria.Subgroup_3	3.25	Winter	3.01	0.00
Acidobacteria.Acidobacteria.Subgroup_3.PAUC26f	3.25	Winter	3.01	0.00
Acidobacteria.Acidobacteria.Subgroup_3.PAUC26f.unclassified	3.25	Winter	3.01	0.00
Acidobacteria.Acidobacteria.Subgroup_3.PAUC26f.unclassified.OTU99_000837	3.02	Winter	2.82	0.04
Acidobacteria.Acidobacteria.Subgroup_3.PAUC26f.unclassified.OTU99_002133	2.87	Winter	2.57	0.01
Acidobacteria.Acidobacteria.Subgroup_5	2.41	Winter	2.30	0.04
Acidobacteria.Acidobacteria.Subgroup_5.unclassified	2.41	Winter	2.30	0.04
Acidobacteria.Acidobacteria.Subgroup_5.unclassified.unclassified	2.41	Winter	2.30	0.04
Acidobacteria.Acidobacteria.Subgroup_6	3.47	Winter	3.26	0.00
Acidobacteria.Acidobacteria.Subgroup_6.unclassified	3.47	Winter	3.26	0.00
Acidobacteria.Acidobacteria.Subgroup_6.unclassified.unclassified	3.47	Winter	3.26	0.00
Actinobacteria.Acidimicrobiia	4.65	Winter	3.96	0.01
Actinobacteria.Acidimicrobiia.Acidimicrobiales	4.65	Winter	3.96	0.01
Actinobacteria.Acidimicrobiia.Acidimicrobiales.Acidimicrobiaceae	4.42	Winter	3.91	0.01
Actinobacteria.Acidimicrobiia.Acidimicrobiales.Acidimicrobiaceae.CL500_29_marine_group.OTU99_000077	3.17	Winter	2.89	0.02
Actinobacteria.Acidimicrobiia.Acidimicrobiales.Acidimicrobiaceae.CL500_29_marine_group.OTU99_000733	2.77	Winter	2.53	0.01
Actinobacteria.Acidimicrobiia.Acidimicrobiales.Acidimicrobiaceae.CL500_29_marine_group.OTU99_000824	3.27	Winter	2.84	0.02
Actinobacteria.Acidimicrobiia.Acidimicrobiales.Acidimicrobiaceae.uncultured	3.99	Winter	3.60	0.00
Actinobacteria.Acidimicrobiia.Acidimicrobiales.Acidimicrobiaceae.uncultured.OTU99_000043	3.84	Winter	3.47	0.00
Actinobacteria.Acidimicrobiia.Acidimicrobiales.Acidimicrobiaceae.uncultured.OTU99_000141	3.19	Winter	2.87	0.02
Actinobacteria.Acidimicrobiia.Acidimicrobiales.OM1_clade	3.66	Winter	3.08	0.04
Actinobacteria.Acidimicrobiia.Acidimicrobiales.OM1_clade.unclassified	2.53	Winter	2.30	0.00
Actinobacteria.Acidimicrobiia.Acidimicrobiales.Sva0996_marine_group	4.01	Winter	3.63	0.00
Actinobacteria.Acidimicrobiia.Acidimicrobiales.Sva0996_marine_group.unclassified	4.01	Winter	3.63	0.00
Actinobacteria.Acidimicrobiia.Acidimicrobiales.Sva0996_marine_group.unclassified.OTU99_000286	3.43	Winter	3.04	0.02
Actinobacteria.Acidimicrobiia.Acidimicrobiales.Sva0996_marine_group.unclassified.OTU99_000788	2.76	Winter	2.58	0.01
Actinobacteria.Acidimicrobiia.Acidimicrobiales.Sva0996_marine_group.unclassified.OTU99_002028	2.87	Winter	2.56	0.02
Actinobacteria.Acidimicrobiia.Acidimicrobiales.uncultured.unclassified.OTU99_000399	3.02	Winter	2.73	0.02
Actinobacteria.Actinobacteria.Corynebacteriales	4.49	Winter	4.17	0.00
Actinobacteria.Actinobacteria.Corynebacteriales.Mycobacteriaceae	3.64	Winter	3.31	0.00
Actinobacteria.Actinobacteria.Corynebacteriales.Mycobacteriaceae.Mycobacterium	3.64	Winter	3.31	0.00
Actinobacteria.Actinobacteria.Corynebacteriales.Mycobacteriaceae.Mycobacterium.OTU99_000216	3.42	Winter	3.10	0.02
Actinobacteria.Actinobacteria.Corynebacteriales.unclassified	4.42	Winter	4.10	0.00
Actinobacteria.Actinobacteria.Corynebacteriales.unclassified.unclassified	4.42	Winter	4.10	0.00
Actinobacteria.Actinobacteria.Corynebacteriales.unclassified.unclassified.OTU99_000035	4.25	Winter	3.95	0.00
Actinobacteria.Actinobacteria.Corynebacteriales.unclassified.unclassified.OTU99_000922	3.13	Winter	2.82	0.02
Actinobacteria.Actinobacteria.Micrococcales.unclassified	3.83	Winter	3.42	0.01
Actinobacteria.Actinobacteria.Micrococcales.unclassified.unclassified	3.83	Winter	3.42	0.01
Actinobacteria.Actinobacteria.Micrococcales.unclassified.unclassified.OTU99_000038	3.19	Winter	2.90	0.02
Actinobacteria.Actinobacteria.Micrococcales.unclassified.unclassified.OTU99_000746	3.23	Winter	2.96	0.02
Actinobacteria.Actinobacteria.PeM15	4.29	Winter	3.63	0.02
Actinobacteria.Actinobacteria.PeM15.unclassified	4.29	Winter	3.63	0.02
Actinobacteria.Actinobacteria.PeM15.unclassified.unclassified	4.29	Winter	3.63	0.02
Actinobacteria.Actinobacteria.PeM15.unclassified.unclassified.OTU99_000317	3.15	Winter	3.00	0.04
Actinobacteria.Actinobacteria.PeM15.unclassified.unclassified.OTU99_000346	3.37	Winter	3.10	0.00
Actinobacteria.Actinobacteria.PeM15.unclassified.unclassified.OTU99_001248	2.74	Winter	2.55	0.04
Actinobacteria.Actinobacteria.PeM15.unclassified.unclassified.OTU99_001390	2.81	Winter	2.60	0.04
Actinobacteria.Actinobacteria.unclassified	3.39	Winter	2.99	0.00
Actinobacteria.Actinobacteria.unclassified.unclassified	3.39	Winter	2.99	0.00
Actinobacteria.Actinobacteria.unclassified.unclassified.unclassified	3.39	Winter	2.99	0.00
Actinobacteria.Actinobacteria.unclassified.unclassified.unclassified.OTU99_002070	2.62	Winter	2.43	0.04
Actinobacteria.Nitriliruptoria	3.00	Winter	2.69	0.00
Actinobacteria.Nitriliruptoria.Nitriliruptorales	3.00	Winter	2.69	0.00
Actinobacteria.Nitriliruptoria.Nitriliruptorales.Nitriliruptoraceae	3.00	Winter	2.69	0.00
Actinobacteria.Nitriliruptoria.Nitriliruptorales.Nitriliruptoraceae.Nitriliruptor	3.00	Winter	2.69	0.00
Actinobacteria.Nitriliruptoria.Nitriliruptorales.Nitriliruptoraceae.Nitriliruptor.OTU99_001068	2.53	Winter	2.54	0.02
Actinobacteria.Thermoleophilia	3.46	Winter	3.09	0.00
Actinobacteria.Thermoleophilia.Gaiellales.uncultured.unclassified.OTU99_001509	2.59	Winter	2.45	0.04
Actinobacteria.Thermoleophilia.Solirubrobacterales	3.34	Winter	2.97	0.00
Actinobacteria.Thermoleophilia.Solirubrobacterales.480_2	3.08	Winter	2.78	0.00
Actinobacteria.Thermoleophilia.Solirubrobacterales.480_2.unclassified	3.08	Winter	2.78	0.00
Actinobacteria.Thermoleophilia.Solirubrobacterales.480_2.unclassified.OTU99_000687	2.80	Winter	2.58	0.04

Taxonomy	LDA	Envrion-ment	Wilcoxon rank-sum test	Kruskal-Wallis
Actinobacteria.Thermoleophilia.Solirubrobacterales.unclassified	3.00	Winter	2.57	0.03
Actinobacteria.Thermoleophilia.Solirubrobacterales.unclassified.unclassified	3.00	Winter	2.58	0.03
Actinobacteria.Thermoleophilia.Solirubrobacterales.unclassified.unclassified.OTU99_001148	2.74	Winter	2.38	0.04
Actinobacteria.unclassified	3.82	Winter	3.45	0.04
Actinobacteria.unclassified.unclassified	3.82	Winter	3.45	0.04
Actinobacteria.unclassified.unclassified.unclassified	3.82	Winter	3.45	0.04
Actinobacteria.unclassified.unclassified.unclassified.unclassified	3.82	Winter	3.45	0.04
Actinobacteria.unclassified.unclassified.unclassified.unclassified.OTU99_000187	3.63	Winter	3.46	0.02
Actinobacteria.unclassified.unclassified.unclassified.unclassified.OTU99_000288	2.72	Winter	2.48	0.04
Actinobacteria.unclassified.unclassified.unclassified.unclassified.OTU99_001731	2.62	Winter	2.34	0.04
AEGEAN_245	3.30	Winter	2.99	0.00
AEGEAN_245.unclassified	3.30	Winter	2.99	0.00
AEGEAN_245.unclassified.unclassified	3.30	Winter	2.99	0.00
AEGEAN_245.unclassified.unclassified.unclassified	3.30	Winter	2.99	0.00
Alphaproteobacteria.Rhizobiales.uncultured	2.70	Winter	2.44	0.00
Alphaproteobacteria.Rhizobiales.uncultured.unclassified	2.70	Winter	2.46	0.00
Alphaproteobacteria.Rhodobacterales.Rhodobacteraceae.Lentibacter	3.16	Winter	2.78	0.02
Alphaproteobacteria.Rhodobacterales.Rhodobacteraceae.unclassified.OTU99_000016	4.47	Winter	4.08	0.00
Alphaproteobacteria.Rhodobacterales.Rhodobacteraceae.unclassified.OTU99_000180	3.70	Winter	3.35	0.03
Alphaproteobacteria.Rhodobacterales.Rhodobacteraceae.unclassified.OTU99_000764	3.18	Winter	3.00	0.02
Alphaproteobacteria.Rhodobacterales.Rhodobacteraceae.unclassified.OTU99_001082	3.07	Winter	2.75	0.04
Alphaproteobacteria.Rhodobacterales.Rhodobacteraceae.unclassified.OTU99_001591	2.78	Winter	2.42	0.04
Alphaproteobacteria.Rhodobacterales.Rhodobacteraceae.uncultured	3.73	Winter	3.23	0.04
Alphaproteobacteria.Rhodospirillales	4.46	Winter	3.98	0.01
Alphaproteobacteria.Rhodospirillales.Acetobacteraceae	3.10	Winter	2.89	0.03
Alphaproteobacteria.Rhodospirillales.Acetobacteraceae.unclassified	3.10	Winter	2.90	0.03
Alphaproteobacteria.Rhodospirillales.Acetobacteraceae.unclassified.OTU99_002040	2.82	Winter	2.65	0.02
Alphaproteobacteria.Rhodospirillales.Rhodospirillaceae.AEGEAN_169_marine_group.OTU99_000894	2.68	Winter	2.52	0.02
Alphaproteobacteria.Rhodospirillales.Rhodospirillaceae.AEGEAN_169_marine_group.OTU99_002782	2.59	Winter	2.54	0.04
Alphaproteobacteria.Rhodospirillales.Rhodospirillaceae.Defluviicoccus	3.44	Winter	3.11	0.00
Alphaproteobacteria.Rhodospirillales.Rhodospirillaceae.unclassified.OTU99_000947	2.70	Winter	2.56	0.04
Alphaproteobacteria.Rhodospirillales.Rhodospirillaceae.uncultured	3.70	Winter	3.29	0.00
Alphaproteobacteria.Rhodospirillales.Rhodospirillaceae.uncultured.OTU99_001523	2.88	Winter	2.63	0.03
Alphaproteobacteria.Rhodospirillales.Rhodospirillaceae.uncultured.OTU99_001586	2.93	Winter	2.71	0.00
Alphaproteobacteria.Rhodospirillales.Rhodospirillales_Incertae_Sedis	3.69	Winter	3.43	0.00
Alphaproteobacteria.Rhodospirillales.Rhodospirillales_Incertae_Sedis.Reyranella	3.69	Winter	3.43	0.00
Alphaproteobacteria.Rhodospirillales.Rhodospirillales_Incertae_Sedis.Reyranella.OTU99_000249	3.58	Winter	3.33	0.00
Alphaproteobacteria.Rhodospirillales.unclassified	3.39	Winter	3.18	0.00
Alphaproteobacteria.Rhodospirillales.unclassified.unclassified	3.39	Winter	3.18	0.00
Alphaproteobacteria.Rhodospirillales.unclassified.unclassified.OTU99_002083	2.63	Winter	2.57	0.04
Alphaproteobacteria.Rhodospirillales.unclassified.unclassified.OTU99_002362	2.62	Winter	2.46	0.04
Alphaproteobacteria.Rickettsiales.EF100_94H03	3.19	Winter	2.93	0.00
Alphaproteobacteria.Rickettsiales.EF100_94H03.unclassified	3.19	Winter	2.93	0.00
Alphaproteobacteria.Rickettsiales.EF100_94H03.unclassified.OTU99_000818	2.86	Winter	2.61	0.01
Alphaproteobacteria.Rickettsiales.SAR116_clade.unclassified.OTU99_000148	3.52	Winter	3.25	0.00
Alphaproteobacteria.Rickettsiales.SAR116_clade.unclassified.OTU99_000238	3.33	Winter	2.91	0.03
Alphaproteobacteria.Rickettsiales.T9d	3.58	Winter	3.18	0.00
Alphaproteobacteria.Rickettsiales.T9d.unclassified	3.58	Winter	3.18	0.00
Alphaproteobacteria.Rickettsiales.T9d.unclassified.OTU99_000271	3.11	Winter	2.88	0.00
Alphaproteobacteria.Rickettsiales.T9d.unclassified.OTU99_000650	2.87	Winter	2.65	0.01
Alphaproteobacteria.Rickettsiales.T9d.unclassified.OTU99_008319	2.36	Winter	2.43	0.04
Alphaproteobacteria.S26_47	2.51	Winter	2.31	0.01
Alphaproteobacteria.S26_47.unclassified	2.51	Winter	2.30	0.01
Alphaproteobacteria.S26_47.unclassified.unclassified	2.51	Winter	2.32	0.01
Alphaproteobacteria.SAR11_clade	4.99	Winter	4.34	0.01
Alphaproteobacteria.SAR11_clade.Surface_1	4.61	Winter	4.03	0.00
Alphaproteobacteria.SAR11_clade.Surface_1.unclassified	4.61	Winter	4.03	0.00
Alphaproteobacteria.SAR11_clade.Surface_1.unclassified.OTU99_000027	4.21	Winter	3.75	0.02
Alphaproteobacteria.SAR11_clade.unclassified	4.36	Winter	3.96	0.00
Alphaproteobacteria.SAR11_clade.unclassified.unclassified	4.36	Winter	3.96	0.00
Alphaproteobacteria.SAR11_clade.unclassified.unclassified.OTU99_001069	3.00	Winter	2.77	0.04
Alphaproteobacteria.SB1_18	3.24	Winter	2.99	0.00
Alphaproteobacteria.SB1_18.unclassified	3.24	Winter	2.99	0.00
Alphaproteobacteria.SB1_18.unclassified.unclassified	3.24	Winter	2.99	0.00

Taxonomy	LDA	Envrion-ment	Wilcoxon rank-sum test	Kruskal-Wallis
Alphaproteobacteria.SB1_18.unclassified.unclassified.OTU99_000634	3.01	Winter	2.76	0.00
Alphaproteobacteria.Sphingomonadales.Sphingomonadaceae.Sphingomonas	2.63	Winter	2.41	0.02
Alphaproteobacteria.Sphingomonadales.unclassified	2.74	Winter	2.29	0.01
Alphaproteobacteria.Sphingomonadales.unclassified.unclassified	2.74	Winter	2.30	0.01
Alphaproteobacteria.Sphingomonadales.unclassified.unclassified.OTU99_008371	2.23	Winter	2.12	0.04
Alphaproteobacteria.unclassified.unclassified.unclassified.OTU99_002097	2.41	Winter	2.56	0.04
Bacteroidetes.Cytophagia.Order_II	3.21	Winter	2.98	0.00
Bacteroidetes.Cytophagia.Order_II.Rhodothermaceae	3.21	Winter	2.98	0.00
Bacteroidetes.Cytophagia.Order_II.Rhodothermaceae.uncultured	3.21	Winter	2.98	0.00
Bacteroidetes.Cytophagia.Order_II.Rhodothermaceae.uncultured.OTU99_000637	2.93	Winter	2.68	0.03
Bacteroidetes.Flavobacteriia.Flavobacteriales.Cryomorphaceae.Crocinitomix	2.37	Winter	2.63	0.04
Bacteroidetes.Flavobacteriia.Flavobacteriales.Cryomorphaceae.Fluviicola.OTU99_004207	2.22	Winter	2.10	0.04
Bacteroidetes.Flavobacteriia.Flavobacteriales.Cryomorphaceae.Fluviicola.OTU99_005026	2.41	Winter	2.95	0.04
Bacteroidetes.Flavobacteriia.Flavobacteriales.Cryomorphaceae.Fluviicola.OTU99_007786	2.41	Winter	2.28	0.04
Bacteroidetes.Flavobacteriia.Flavobacteriales.Cryomorphaceae.Fluviicola.OTU99_013061	2.23	Winter	2.18	0.04
Bacteroidetes.Flavobacteriia.Flavobacteriales.Cryomorphaceae.Owenweeksia	4.08	Winter	3.52	0.01
Bacteroidetes.Flavobacteriia.Flavobacteriales.Cryomorphaceae.Owenweeksia.OTU99_000145	3.85	Winter	3.56	0.00
Bacteroidetes.Flavobacteriia.Flavobacteriales.Cryomorphaceae.unclassified.OTU99_000063	3.56	Winter	3.09	0.04
Bacteroidetes.Flavobacteriia.Flavobacteriales.Flavobacteriaceae.Flavobacterium.OTU99_001434	2.68	Winter	2.41	0.04
Bacteroidetes.Flavobacteriia.Flavobacteriales.Flavobacteriaceae.NS4_marine_group.OTU99_000206	3.21	Winter	2.83	0.00
Bacteroidetes.Flavobacteriia.Flavobacteriales.Flavobacteriaceae.Ulvibacter	3.93	Winter	3.42	0.01
Bacteroidetes.Flavobacteriia.Flavobacteriales.Flavobacteriaceae.Ulvibacter.OTU99_000154	3.65	Winter	3.29	0.00
Bacteroidetes.Flavobacteriia.Flavobacteriales.Flavobacteriaceae.unclassified.OTU99_000031	4.26	Winter	3.85	0.00
Bacteroidetes.Flavobacteriia.Flavobacteriales.Flavobacteriaceae.unclassified.OTU99_000059	4.09	Winter	3.76	0.00
Bacteroidetes.Flavobacteriia.Flavobacteriales.NS7_marine_group.unclassified.OTU99_000739	2.90	Winter	2.52	0.03
Bacteroidetes.Flavobacteriia.Flavobacteriales.NS9_marine_group	4.12	Winter	3.66	0.00
Bacteroidetes.Flavobacteriia.Flavobacteriales.NS9_marine_group.unclassified	4.12	Winter	3.66	0.00
Bacteroidetes.Flavobacteriia.Flavobacteriales.NS9_marine_group.unclassified.OTU99_000051	3.81	Winter	3.54	0.02
Bacteroidetes.Flavobacteriia.Flavobacteriales.NS9_marine_group.unclassified.OTU99_000203	2.76	Winter	2.44	0.00
Bacteroidetes.Flavobacteriia.Flavobacteriales.NS9_marine_group.unclassified.OTU99_000230	2.92	Winter	2.65	0.04
Bacteroidetes.Flavobacteriia.Flavobacteriales.NS9_marine_group.unclassified.OTU99_001146	2.66	Winter	2.43	0.01
Bacteroidetes.Flavobacteriia.Flavobacteriales.NS9_marine_group.unclassified.OTU99_001416	2.77	Winter	2.57	0.02
Bacteroidetes.Flavobacteriia.Flavobacteriales.NS9_marine_group.unclassified.OTU99_002353	2.57	Winter	2.45	0.04
Bacteroidetes.Flavobacteriia.Flavobacteriales.NS9_marine_group.unclassified.OTU99_003939	2.68	Winter	2.37	0.04
Bacteroidetes.Flavobacteriia.Flavobacteriales.unclassified.unclassified.OTU99_000518	3.20	Winter	2.90	0.01
Bacteroidetes.Sphingobacteriia.Sphingobacteriales.NS11_12_marine_group.unclassified.OTU99_000962	2.77	Winter	2.58	0.02
Bacteroidetes.Sphingobacteriia.Sphingobacteriales.Sphingobacteriaceae	3.21	Winter	2.93	0.02
Bacteroidetes.Sphingobacteriia.Sphingobacteriales.Sphingobacteriaceae.Pedobacter	3.18	Winter	2.89	0.02
Bacteroidetes.Sphingobacteriia.Sphingobacteriales.Sphingobacteriaceae.Pedobacter.OTU99_000519	2.96	Winter	2.77	0.04
Bacteroidetes.Sphingobacteriia.Sphingobacteriales.unclassified	3.36	Winter	3.14	0.00
Bacteroidetes.Sphingobacteriia.Sphingobacteriales.unclassified.unclassified	3.36	Winter	3.14	0.00
Bacteroidetes.Sphingobacteriia.Sphingobacteriales.unclassified.unclassified.OTU99_000472	3.17	Winter	3.00	0.04
Bacteroidetes.Sphingobacteriia.Sphingobacteriales.unclassified.unclassified.OTU99_001418	2.75	Winter	2.51	0.00
bacteroidetes.unclassified.unclassified.unclassified.unclassified.OTU99_000446	2.66	Winter	2.44	0.00
BD1_5	2.44	Winter	2.14	0.02
BD1_5.unclassified	2.44	Winter	2.13	0.02
BD1_5.unclassified.unclassified	2.44	Winter	2.13	0.02
BD1_5.unclassified.unclassified.unclassified	2.44	Winter	2.12	0.02
BD1_5.unclassified.unclassified.unclassified.unclassified	2.44	Winter	2.14	0.02
Betaproteobacteria	4.75	Winter	3.92	0.03
Betaproteobacteria.Burkholderiales.Alcaligenaceae.unclassified.OTU99_001173	3.01	Winter	3.35	0.04
Betaproteobacteria.Burkholderiales.Comamonadaceae.BAL58_marine_group.OTU99_000930	3.07	Winter	2.68	0.01
Betaproteobacteria.Burkholderiales.Comamonadaceae.BAL58_marine_group.OTU99_001290	2.84	Winter	2.64	0.04
Betaproteobacteria.Hydrogenophilales	3.73	Winter	3.44	0.00
Betaproteobacteria.Hydrogenophilales.Hydrogenophilaceae	3.73	Winter	3.44	0.00
Betaproteobacteria.Hydrogenophilales.Hydrogenophilaceae.Thiobacillus	3.73	Winter	3.44	0.00
Betaproteobacteria.Hydrogenophilales.Hydrogenophilaceae.Thiobacillus.OTU99_000421	3.53	Winter	3.30	0.03
Betaproteobacteria.Methylophilales	4.13	Winter	3.51	0.00
Betaproteobacteria.Methylophilales.Methylophilaceae	4.13	Winter	3.51	0.00
Betaproteobacteria.Methylophilales.Methylophilaceae.OM43_clade	4.12	Winter	3.47	0.00
Betaproteobacteria.Methylophilales.Methylophilaceae.OM43_clade.OTU99_000475	3.44	Winter	3.17	0.02
Betaproteobacteria.Methylophilales.Methylophilaceae.OM43_clade.OTU99_000548	3.20	Winter	2.88	0.02
Betaproteobacteria.Nitrosomonadales	3.35	Winter	3.08	0.00
Betaproteobacteria.Nitrosomonadales.Nitrosomonadaceae	3.23	Winter	2.95	0.00

Taxonomy	LDA	Envrion-ment	Wilcoxon rank-sum test	Kruskal-Wallis
Betaproteobacteria.Nitrosomonadales.Nitrosomonadaceae.Nitrosomonas	3.20	Winter	2.93	0.00
Betaproteobacteria.Nitrosomonadales.unclassified	2.74	Winter	2.52	0.00
Betaproteobacteria.Nitrosomonadales.unclassified.unclassified	2.74	Winter	2.52	0.00
Betaproteobacteria.Nitrosomonadales.unclassified.unclassified.OTU99_003545	2.63	Winter	2.43	0.01
Betaproteobacteria.TRA3_20.unclassified.unclassified.OTU99_002648	2.32	Winter	2.18	0.04
Betaproteobacteria.unclassified	4.01	Winter	3.61	0.01
Betaproteobacteria.unclassified.unclassified	4.01	Winter	3.61	0.01
Betaproteobacteria.unclassified.unclassified.unclassified	4.01	Winter	3.61	0.01
Betaproteobacteria.unclassified.unclassified.unclassified.OTU99_000603	3.30	Winter	3.01	0.01
Betaproteobacteria.unclassified.unclassified.unclassified.OTU99_000984	2.89	Winter	2.63	0.02
Betaproteobacteria.unclassified.unclassified.unclassified.OTU99_002860	2.37	Winter	2.24	0.04
Candidate_division_BRC1	2.88	Winter	2.64	0.01
Candidate_division_BRC1.unclassified	2.88	Winter	2.64	0.01
Candidate_division_BRC1.unclassified.unclassified	2.88	Winter	2.65	0.01
Candidate_division_BRC1.unclassified.unclassified.unclassified	2.88	Winter	2.65	0.01
Candidate_division_BRC1.unclassified.unclassified.unclassified.unclassified	2.88	Winter	2.64	0.01
Chlorobi	2.99	Winter	2.73	0.00
Chlorobi.Chlorobia	2.99	Winter	2.75	0.00
Chlorobi.Chlorobia.Chlorobiales	2.99	Winter	2.75	0.00
Chlorobi.Chlorobia.Chlorobiales.OPB56	2.99	Winter	2.75	0.00
Chlorobi.Chlorobia.Chlorobiales.OPB56.unclassified	2.99	Winter	2.75	0.00
Chloroflexi	4.18	Winter	3.92	0.00
Chloroflexi.Caldilineae	3.82	Winter	3.57	0.00
Chloroflexi.Caldilineae.Caldilineales	3.82	Winter	3.57	0.00
Chloroflexi.Caldilineae.Caldilineales.Caldilineaceae	3.82	Winter	3.57	0.00
Chloroflexi.Caldilineae.Caldilineales.Caldilineaceae.uncultured	3.82	Winter	3.57	0.00
Chloroflexi.Caldilineae.Caldilineales.Caldilineaceae.uncultured.OTU99_000079	3.74	Winter	3.51	0.00
Chloroflexi.JG30_KF_CM66	3.36	Winter	3.12	0.00
Chloroflexi.JG30_KF_CM66.unclassified	3.36	Winter	3.12	0.00
Chloroflexi.JG30_KF_CM66.unclassified.unclassified	3.36	Winter	3.12	0.00
Chloroflexi.JG30_KF_CM66.unclassified.unclassified.unclassified	3.36	Winter	3.12	0.00
Chloroflexi.JG30_KF_CM66.unclassified.unclassified.unclassified.OTU99_001178	2.68	Winter	2.43	0.04
Chloroflexi.JG30_KF_CM66.unclassified.unclassified.unclassified.OTU99_005507	2.37	Winter	2.32	0.02
Chloroflexi.SAR202_clade	3.73	Winter	3.46	0.00
Chloroflexi.SAR202_clade.unclassified	3.73	Winter	3.46	0.00
Chloroflexi.SAR202_clade.unclassified.unclassified	3.73	Winter	3.46	0.00
Chloroflexi.SAR202_clade.unclassified.unclassified.unclassified	3.73	Winter	3.46	0.00
Chloroflexi.SAR202_clade.unclassified.unclassified.unclassified.OTU99_000300	2.86	Winter	2.60	0.00
Cyanobacteria.Cyanobacteria.SubsectionI.FamilyI.Synechococcus.OTU99_000004	3.69	Winter	3.39	0.04
Cyanobacteria.Cyanobacteria.SubsectionI.FamilyI.Synechococcus.OTU99_000213	2.84	Winter	2.57	0.04
Cyanobacteria.Cyanobacteria.SubsectionI.FamilyI.Synechococcus.OTU99_000228	3.46	Winter	3.16	0.04
Cyanobacteria.Cyanobacteria.SubsectionI.FamilyI.Synechococcus.OTU99_000543	3.29	Winter	2.99	0.01
Cyanobacteria.Cyanobacteria.SubsectionI.FamilyI.unclassified	3.97	Winter	3.50	0.00
Cyanobacteria.Cyanobacteria.SubsectionI.FamilyI.unclassified.OTU99_000151	3.63	Winter	3.34	0.00
Deltaproteobacteria	4.11	Winter	3.66	0.03
Deltaproteobacteria.Desulfobacterales	3.70	Winter	3.45	0.00
Deltaproteobacteria.Desulfobacterales.Nitrospinaceae	3.69	Winter	3.44	0.00
Deltaproteobacteria.Desulfobacterales.Nitrospinaceae.Nitrospina	3.69	Winter	3.44	0.00
Deltaproteobacteria.Desulfobacterales.Nitrospinaceae.Nitrospina.OTU99_000285	3.34	Winter	3.12	0.01
Deltaproteobacteria.SAR324_clade_Marine_group_B_	3.43	Winter	3.10	0.04
Deltaproteobacteria.SAR324_clade_Marine_group_B_.unclassified	3.43	Winter	3.12	0.04
Deltaproteobacteria.SAR324_clade_Marine_group_B_.unclassified.unclassified	3.43	Winter	3.11	0.04
Deltaproteobacteria.Sh765B_TzT_29	3.25	Winter	2.94	0.00
Deltaproteobacteria.Sh765B_TzT_29.unclassified	3.25	Winter	2.94	0.00
Deltaproteobacteria.Sh765B_TzT_29.unclassified.unclassified	3.25	Winter	2.94	0.00
Firmicutes	3.41	Winter	3.14	0.00
Firmicutes.Bacilli	3.14	Winter	2.86	0.00
Firmicutes.Bacilli.Bacillales	3.08	Winter	2.80	0.00
Firmicutes.Bacilli.Bacillales.Paenibacillaceae	3.06	Winter	2.79	0.00
Firmicutes.Bacilli.Bacillales.Paenibacillaceae.Paenibacillus	2.42	Winter	2.31	0.04
Firmicutes.Bacilli.Bacillales.Paenibacillaceae.unclassified	2.95	Winter	2.69	0.01
Firmicutes.Bacilli.Bacillales.Paenibacillaceae.unclassified.OTU99_000806	2.95	Winter	2.69	0.01
Firmicutes.Clostridia	3.04	Winter	2.80	0.00
Firmicutes.Clostridia.Clostridiales	3.04	Winter	2.80	0.00

Taxonomy	LDA	Envrion-ment	Wilcoxon rank-sum test	Kruskal-Wallis
Firmicutes.Clostridia.Clostridiales.Family_XVIII	3.03	Winter	2.79	0.00
Firmicutes.Clostridia.Clostridiales.Family_XVIII.uncultured	3.01	Winter	2.78	0.00
Firmicutes.Clostridia.Clostridiales.Family_XVIII.uncultured.OTU99_001590	2.74	Winter	2.53	0.02
Firmicutes.Clostridia.Clostridiales.Family_XVIII.uncultured.OTU99_003686	2.39	Winter	2.20	0.04
Gammaproteobacteria.Alteromonadales.Alteromonadaceae.OM60_NOR5__clade.OTU99_000641	3.22	Winter	2.93	0.01
Gammaproteobacteria.Alteromonadales.Alteromonadaceae.SAR92_clade.OTU99_000805	3.21	Winter	2.93	0.00
Gammaproteobacteria.Alteromonadales.Pseudoalteromonadaceae	3.01	Winter	2.70	0.04
Gammaproteobacteria.Alteromonadales.Pseudoalteromonadaceae.Pseudoalteromonas	3.01	Winter	2.71	0.04
Gammaproteobacteria.Alteromonadales.Pseudoalteromonadaceae.Pseudoalteromonas.OTU99_000195	2.84	Winter	2.67	0.04
Gammaproteobacteria.Chromatiales	2.99	Winter	2.70	0.00
Gammaproteobacteria.Chromatiales.unclassified	2.92	Winter	2.65	0.00
Gammaproteobacteria.Chromatiales.unclassified.unclassified	2.92	Winter	2.65	0.00
Gammaproteobacteria.Legionellales	3.43	Winter	3.13	0.00
Gammaproteobacteria.Legionellales.Legionellaceae	3.39	Winter	3.10	0.00
Gammaproteobacteria.Legionellales.Legionellaceae.uncultured	3.20	Winter	2.91	0.00
Gammaproteobacteria.Legionellales.Legionellaceae.uncultured.OTU99_000989	2.49	Winter	2.33	0.01
Gammaproteobacteria.Oceanospirillales.Oceanospirillaceae	3.90	Winter	3.40	0.03
Gammaproteobacteria.Oceanospirillales.Oceanospirillaceae.Pseudospirillum	3.88	Winter	3.40	0.03
Gammaproteobacteria.Oceanospirillales.Oceanospirillaceae.Pseudospirillum.OTU99_000259	3.36	Winter	3.11	0.01
Gammaproteobacteria.Oceanospirillales.Oceanospirillaceae.Pseudospirillum.OTU99_001471	2.79	Winter	2.61	0.04
Gammaproteobacteria.Oceanospirillales.OM182_clade.unclassified.OTU99_001250	2.87	Winter	2.68	0.02
Gammaproteobacteria.Oceanospirillales.unclassified	4.12	Winter	3.80	0.00
Gammaproteobacteria.Oceanospirillales.unclassified.unclassified	4.12	Winter	3.80	0.00
Gammaproteobacteria.Pseudomonadales.Moraxellaceae.Psychrobacter.OTU99_000963	2.35	Winter	2.16	0.04
Gammaproteobacteria.Pseudomonadales.Pseudomonadaceae.Pseudomonas.OTU99_002154	2.68	Winter	2.32	0.04
Gammaproteobacteria.Thiotrichales.Piscirickettsiaceae.Cycloclasticus	2.55	Winter	2.40	0.04
Gammaproteobacteria.unclassified.unclassified.unclassified.OTU99_001035	2.48	Winter	2.40	0.02
Gammaproteobacteria.Xanthomonadales	3.23	Winter	2.92	0.00
Gammaproteobacteria.Xanthomonadales.JTB255_marine_benthic_group	2.72	Winter	2.46	0.00
Gammaproteobacteria.Xanthomonadales.JTB255_marine_benthic_group.unclassified	2.72	Winter	2.46	0.00
Gammaproteobacteria.Xanthomonadales.unclassified	2.99	Winter	2.74	0.00
Gammaproteobacteria.Xanthomonadales.unclassified.unclassified	2.99	Winter	2.74	0.00
Gammaproteobacteria.Xanthomonadales.unclassified.unclassified.OTU99_001367	2.84	Winter	2.63	0.03
Gemmatimonadetes	3.59	Winter	3.33	0.00
Gemmatimonadetes.Gemmatimonadetes	3.59	Winter	3.33	0.00
Gemmatimonadetes.Gemmatimonadetes.BD2_11_terrestrial_group	3.50	Winter	3.22	0.00
Gemmatimonadetes.Gemmatimonadetes.BD2_11_terrestrial_group.unclassified	3.50	Winter	3.22	0.00
Gemmatimonadetes.Gemmatimonadetes.BD2_11_terrestrial_group.unclassified.unclassified	3.50	Winter	3.22	0.00
Gemmatimonadetes.Gemmatimonadetes.BD2_11_terrestrial_group.unclassified.unclassified.OTU99_000780	2.96	Winter	2.73	0.00
JTB23	2.37	Winter	2.35	0.01
JTB23.unclassified	2.37	Winter	2.36	0.01
JTB23.unclassified.unclassified	2.37	Winter	2.35	0.01
JTB23.unclassified.unclassified.unclassified	2.37	Winter	2.38	0.01
JTB23.unclassified.unclassified.unclassified.OTU99_001703	2.15	Winter	2.39	0.04
Lentisphaerae	3.48	Winter	3.23	0.00
Lentisphaerae.WCHB1_41	3.45	Winter	3.20	0.00
Lentisphaerae.WCHB1_41.unclassified	3.45	Winter	3.20	0.00
Lentisphaerae.WCHB1_41.unclassified.unclassified	3.45	Winter	3.20	0.00
Lentisphaerae.WCHB1_41.unclassified.unclassified.unclassified	3.45	Winter	3.20	0.00
Lentisphaerae.WCHB1_41.unclassified.unclassified.unclassified.OTU99_001246	2.96	Winter	2.71	0.04
Planctomycetes	4.84	Winter	4.40	0.00
Planctomycetes.Phycisphaerae	4.07	Winter	3.74	0.00
Planctomycetes.Phycisphaerae.Phycisphaerales	4.07	Winter	3.74	0.00
Planctomycetes.Phycisphaerae.Phycisphaerales.Phycisphaeraceae	4.07	Winter	3.74	0.00
Planctomycetes.Phycisphaerae.Phycisphaerales.Phycisphaeraceae.CL500_3	3.78	Winter	3.48	0.00
Planctomycetes.Phycisphaerae.Phycisphaerales.Phycisphaeraceae.unclassified	3.74	Winter	3.47	0.00
Planctomycetes.Phycisphaerae.Phycisphaerales.Phycisphaeraceae.unclassified.OTU99_000233	3.05	Winter	2.79	0.04
Planctomycetes.Phycisphaerae.Phycisphaerales.Phycisphaeraceae.unclassified.OTU99_000384	3.36	Winter	3.13	0.00
Planctomycetes.Phycisphaerae.Phycisphaerales.Phycisphaeraceae.unclassified.OTU99_000555	3.25	Winter	2.96	0.00
Planctomycetes.Planctomycetacia	4.74	Winter	4.30	0.00
Planctomycetes.Planctomycetacia.Planctomycetales	4.74	Winter	4.30	0.00
Planctomycetes.Planctomycetacia.Planctomycetales.Planctomycetaceae	4.74	Winter	4.30	0.00
Planctomycetes.Planctomycetacia.Planctomycetales.Planctomycetaceae.Planctomyces	4.30	Winter	3.97	0.00
Planctomycetes.Planctomycetacia.Planctomycetales.Planctomycetaceae.Planctomyces.OTU99_000133	3.89	Winter	3.59	0.00

Taxonomy	LDA	Envrion-ment	Wilcoxon rank-sum test	Kruskal-Wallis
Planctomycetes.Planctomycetacia.Planctomycetales.Planctomycetaceae.Planctomyces.OTU99_000481	3.03	Winter	2.74	0.04
Planctomycetes.Planctomycetacia.Planctomycetales.Planctomycetaceae.Planctomyces.OTU99_000608	3.00	Winter	2.67	0.04
Planctomycetes.Planctomycetacia.Planctomycetales.Planctomycetaceae.Planctomyces.OTU99_000995	3.10	Winter	2.77	0.02
Planctomycetes.Planctomycetacia.Planctomycetales.Planctomycetaceae.Planctomyces.OTU99_001629	2.76	Winter	2.55	0.02
Planctomycetes.Planctomycetacia.Planctomycetales.Planctomycetaceae.Planctomyces.OTU99_020521	2.32	Winter	2.19	0.04
Planctomycetes.Planctomycetacia.Planctomycetales.Planctomycetaceae.unclassified	3.50	Winter	3.08	0.02
Planctomycetes.Planctomycetacia.Planctomycetales.Planctomycetaceae.uncultured.OTU99_000081	3.21	Winter	3.02	0.04
Planctomycetes.Planctomycetacia.Planctomycetales.Planctomycetaceae.uncultured.OTU99_000197	3.44	Winter	3.24	0.02
Planctomycetes.Planctomycetacia.Planctomycetales.Planctomycetaceae.uncultured.OTU99_000741	3.07	Winter	2.82	0.04
Planctomycetes.Planctomycetacia.Planctomycetales.Planctomycetaceae.uncultured.OTU99_000959	3.19	Winter	2.93	0.00
Planctomycetes.Planctomycetacia.Planctomycetales.Planctomycetaceae.uncultured.OTU99_001129	3.08	Winter	2.77	0.02
Planctomycetes.Planctomycetacia.Planctomycetales.Planctomycetaceae.uncultured.OTU99_001334	3.02	Winter	2.60	0.04
Planctomycetes.Planctomycetacia.Planctomycetales.Planctomycetaceae.uncultured.OTU99_001588	2.78	Winter	2.78	0.04
SPOTSOCT00m83.unclassified.unclassified.unclassified.OTU99_000562	2.92	Winter	2.64	0.04
Unclassified	3.93	Winter	3.58	0.00
unclassified	4.51	Winter	3.91	0.00
Unclassified.unclassified	3.93	Winter	3.58	0.00
unclassified.unclassified	4.51	Winter	3.91	0.00
Unclassified.unclassified.unclassified	3.93	Winter	3.58	0.00
unclassified.unclassified.unclassified	4.51	Winter	3.91	0.00
Unclassified.unclassified.unclassified.unclassified	3.93	Winter	3.58	0.00
unclassified.unclassified.unclassified.unclassified	4.51	Winter	3.91	0.00
unclassified.unclassified.unclassified.unclassified.OTU99_000537	3.00	Winter	2.76	0.01
unclassified.unclassified.unclassified.unclassified.OTU99_001369	2.37	Winter	2.24	0.02
unclassified.unclassified.unclassified.unclassified.OTU99_001716	2.59	Winter	2.53	0.04
unclassified.unclassified.unclassified.unclassified.OTU99_003343	2.34	Winter	2.20	0.04
unclassified.unclassified.unclassified.unclassified.OTU99_004192	2.49	Winter	2.28	0.04
Unclassified.unclassified.unclassified.unclassified.unclassified	3.93	Winter	3.58	0.00
unclassified.unclassified.unclassified.unclassified.unclassified.OTU99_000297	2.81	Winter	2.76	0.04
unclassified.unclassified.unclassified.unclassified.unclassified.OTU99_000392	2.69	Winter	2.55	0.04
unclassified.unclassified.unclassified.unclassified.unclassified.OTU99_000499	2.96	Winter	2.73	0.01
unclassified.unclassified.unclassified.unclassified.unclassified.OTU99_001000	3.06	Winter	2.88	0.01
unclassified.unclassified.unclassified.unclassified.unclassified.OTU99_002849	2.57	Winter	2.44	0.04
unclassified.unclassified.unclassified.unclassified.unclassified.OTU99_007213	2.55	Winter	2.66	0.04
Unclassified.unclassified.unclassified.unclassified.unclassified.unclassified	3.93	Winter	3.58	0.00
Unclassified.unclassified.unclassified.unclassified.unclassified.unclassified.OTU99_000184	3.50	Winter	3.17	0.00
Unclassified.unclassified.unclassified.unclassified.unclassified.unclassified.OTU99_004952	2.48	Winter	2.36	0.04
Verrucomicrobia.OPB35_soil_group.unclassified.unclassified.unclassified.OTU99_000409	2.92	Winter	2.73	0.04
Verrucomicrobia.Spartobacteria.Chthoniobacterales.LD29	3.42	Winter	3.17	0.01
Verrucomicrobia.Spartobacteria.Chthoniobacterales.LD29.unclassified	3.42	Winter	3.19	0.01
Verrucomicrobia.Spartobacteria.Chthoniobacterales.unclassified.unclassified.OTU99_000101	4.10	Winter	3.83	0.00
Verrucomicrobia.Spartobacteria.Chthoniobacterales.unclassified.unclassified.OTU99_000115	2.90	Winter	2.78	0.04
Verrucomicrobia.Verrucomicrobiae.Verrucomicrobiales.DEV007	3.53	Winter	3.14	0.00
Verrucomicrobia.Verrucomicrobiae.Verrucomicrobiales.DEV007.unclassified	3.53	Winter	3.14	0.00
Verrucomicrobia.Verrucomicrobiae.Verrucomicrobiales.DEV007.unclassified.OTU99_001508	2.95	Winter	2.62	0.04
Verrucomicrobia.Verrucomicrobiae.Verrucomicrobiales.DEV007.unclassified.OTU99_002653	2.51	Winter	2.29	0.04
Verrucomicrobia.Verrucomicrobiae.Verrucomicrobiales.Verrucomicrobiaceae.Roseibacillus.OTU99_000410	3.28	Winter	3.07	0.00
Actinobacteria.Acidimicrobiia.Acidimicrobiales.Acidimicrobiaceae.CL500_29_marine_group.OTU99_000378	3.04	Summer	2.77	0.00
Actinobacteria.Acidimicrobiia.Acidimicrobiales.Acidimicrobiaceae.unclassified.OTU99_001221	3.01	Summer	2.68	0.03
Actinobacteria.Acidimicrobiia.Acidimicrobiales.Acidimicrobiaceae.uncultured.OTU99_008235	2.42	Summer	2.49	0.02
Actinobacteria.Acidimicrobiia.Acidimicrobiales.uncultured.unclassified.OTU99_000162	4.06	Summer	3.76	0.00
Actinobacteria.Actinobacteria.Frankiales	4.82	Summer	4.24	0.03
Actinobacteria.Actinobacteria.Frankiales.Sporichthyaceae	4.82	Summer	4.24	0.03
Actinobacteria.Actinobacteria.Frankiales.Sporichthyaceae.hgcI_clade	4.80	Summer	4.22	0.04
Actinobacteria.Actinobacteria.Frankiales.Sporichthyaceae.hgcI_clade.OTU99_000045	4.13	Summer	3.79	0.00
Actinobacteria.Actinobacteria.Frankiales.Sporichthyaceae.hgcI_clade.OTU99_000147	3.79	Summer	3.44	0.00
Actinobacteria.Actinobacteria.Frankiales.Sporichthyaceae.hgcI_clade.OTU99_000354	3.54	Summer	3.19	0.00
Actinobacteria.Actinobacteria.Frankiales.Sporichthyaceae.hgcI_clade.OTU99_004084	2.40	Summer	2.36	0.04
Actinobacteria.Actinobacteria.Frankiales.Sporichthyaceae.unclassified	3.46	Summer	3.05	0.00
Actinobacteria.Actinobacteria.Frankiales.Sporichthyaceae.unclassified.OTU99_000220	3.41	Summer	3.12	0.00
Actinobacteria.Actinobacteria.Micrococcales.Microbacteriaceae	4.38	Summer	3.96	0.00
Actinobacteria.Actinobacteria.Micrococcales.Microbacteriaceae.Candidatus_Aquiluna	4.02	Summer	3.54	0.00
Actinobacteria.Actinobacteria.Micrococcales.Microbacteriaceae.Candidatus_Aquiluna.OTU99_000110	3.88	Summer	3.48	0.01
Actinobacteria.Actinobacteria.Micrococcales.Microbacteriaceae.unclassified	4.13	Summer	3.75	0.00

Taxonomy	LDA	Envrion-ment	Wilcoxon rank-sum test	Kruskal-Wallis
Actinobacteria.Actinobacteria.Micrococcales.Microbacteriaceae.unclassified.OTU99_000167	4.07	Summer	3.72	0.00
Actinobacteria.Actinobacteria.Micrococcales.Microbacteriaceae.unclassified.OTU99_001789	2.86	Summer	2.63	0.02
Actinobacteria.Actinobacteria.PeM15.unclassified.unclassified.OTU99_000142	3.74	Summer	3.39	0.02
Alphaproteobacteria.Caulobacterales	3.50	Summer	3.10	0.00
Alphaproteobacteria.Caulobacterales.Caulobacteraceae.Brevundimonas	2.79	Summer	2.48	0.04
Alphaproteobacteria.Caulobacterales.Hyphomonadaceae	3.35	Summer	3.04	0.00
Alphaproteobacteria.Caulobacterales.Hyphomonadaceae.Hyphomonas	3.34	Summer	3.03	0.00
Alphaproteobacteria.Caulobacterales.Hyphomonadaceae.Hyphomonas.OTU99_000508	3.15	Summer	2.86	0.00
Alphaproteobacteria.Caulobacterales.Hyphomonadaceae.Hyphomonas.OTU99_001488	2.18	Summer	2.54	0.04
Alphaproteobacteria.DB1_14	3.25	Summer	2.95	0.00
Alphaproteobacteria.DB1_14.unclassified	3.25	Summer	2.95	0.00
Alphaproteobacteria.DB1_14.unclassified.unclassified	3.25	Summer	2.95	0.00
Alphaproteobacteria.DB1_14.unclassified.unclassified.OTU99_000644	3.18	Summer	2.88	0.00
Alphaproteobacteria.Rhizobiales.unclassified.unclassified.OTU99_002344	2.48	Summer	2.97	0.04
Alphaproteobacteria.Rhodobacterales.Rhodobacteraceae.Loktanella	3.87	Summer	3.50	0.00
Alphaproteobacteria.Rhodobacterales.Rhodobacteraceae.Loktanella.OTU99_000116	3.86	Summer	3.52	0.00
Alphaproteobacteria.Rhodobacterales.Rhodobacteraceae.Marivita	2.97	Summer	2.70	0.04
Alphaproteobacteria.Rhodobacterales.Rhodobacteraceae.Marivita.OTU99_000779	2.97	Summer	2.70	0.04
Alphaproteobacteria.Rhodobacterales.Rhodobacteraceae.Sulfitobacter.OTU99_000335	3.16	Summer	2.87	0.04
Alphaproteobacteria.Rhodobacterales.Rhodobacteraceae.unclassified.OTU99_000136	4.04	Summer	3.74	0.00
Alphaproteobacteria.Rhodobacterales.Rhodobacteraceae.unclassified.OTU99_000211	3.94	Summer	3.66	0.00
Alphaproteobacteria.Rhodobacterales.Rhodobacteraceae.unclassified.OTU99_000713	2.73	Summer	2.52	0.02
Alphaproteobacteria.Rhodobacterales.Rhodobacteraceae.unclassified.OTU99_000914	3.18	Summer	2.88	0.01
Alphaproteobacteria.Rhodobacterales.Rhodobacteraceae.unclassified.OTU99_001432	2.90	Summer	2.65	0.00
Alphaproteobacteria.Rhodobacterales.Rhodobacteraceae.unclassified.OTU99_001661	2.71	Summer	2.42	0.04
Alphaproteobacteria.Rhodobacterales.Rhodobacteraceae.unclassified.OTU99_002093	2.40	Summer	2.29	0.04
Alphaproteobacteria.Rhodospirillales.Rhodospirillaceae.Thalassospira	2.99	Summer	2.66	0.04
Alphaproteobacteria.Rhodospirillales.Rhodospirillaceae.Thalassospira.OTU99_000625	2.99	Summer	2.70	0.02
Alphaproteobacteria.Rhodospirillales.Rhodospirillaceae.unclassified.OTU99_000424	3.53	Summer	3.23	0.00
Alphaproteobacteria.Rhodospirillales.Rhodospirillaceae.unclassified.OTU99_001305	2.68	Summer	2.46	0.04
Alphaproteobacteria.Rhodospirillales.Rhodospirillaceae.unclassified.OTU99_002371	2.70	Summer	2.34	0.04
Alphaproteobacteria.Rhodospirillales.Rhodospirillaceae.uncultured.OTU99_001417	2.57	Summer	2.72	0.04
Alphaproteobacteria.Rickettsiales	4.31	Summer	3.57	0.04
Alphaproteobacteria.Rickettsiales.SAR116_clade	4.23	Summer	3.74	0.04
Alphaproteobacteria.Rickettsiales.SAR116_clade.unclassified	4.23	Summer	3.74	0.04
Alphaproteobacteria.Rickettsiales.SAR116_clade.unclassified.OTU99_000803	2.81	Summer	2.57	0.04
Alphaproteobacteria.Rickettsiales.T9d.unclassified.OTU99_001006	2.90	Summer	2.62	0.00
Alphaproteobacteria.SAR11_clade.Surface_4.unclassified.OTU99_000208	3.24	Summer	3.11	0.04
Alphaproteobacteria.Sphingomonadales.Erythrobacteraceae	3.31	Summer	2.98	0.00
Alphaproteobacteria.Sphingomonadales.Erythrobacteraceae.unclassified	3.25	Summer	2.92	0.02
Alphaproteobacteria.Sphingomonadales.Erythrobacteraceae.unclassified.OTU99_000194	3.23	Summer	2.89	0.02
Alphaproteobacteria.unclassified.unclassified.unclassified.OTU99_000192	2.74	Summer	2.49	0.04
Alphaproteobacteria.unclassified.unclassified.unclassified.OTU99_000465	2.26	Summer	2.22	0.04
Alphaproteobacteria.unclassified.unclassified.unclassified.OTU99_000467	2.49	Summer	2.33	0.04
Alphaproteobacteria.unclassified.unclassified.unclassified.OTU99_001087	3.01	Summer	2.68	0.01
Bacteria	6.00	Summer	3.58	0.00
bacteroidetes	4.39	Summer	4.01	0.00
Bacteroidetes.Cytophagia	4.19	Summer	3.70	0.00
Bacteroidetes.Cytophagia.Cytophagales	4.12	Summer	3.70	0.00
Bacteroidetes.Cytophagia.Cytophagales.Cyclobacteriaceae	3.98	Summer	3.59	0.00
Bacteroidetes.Cytophagia.Cytophagales.Cyclobacteriaceae.Algoriphagus	3.47	Summer	3.20	0.00
Bacteroidetes.Cytophagia.Cytophagales.Cyclobacteriaceae.Algoriphagus.OTU99_000152	3.42	Summer	3.15	0.01
Bacteroidetes.Cytophagia.Cytophagales.Cyclobacteriaceae.unclassified	3.81	Summer	3.38	0.00
Bacteroidetes.Cytophagia.Cytophagales.Cyclobacteriaceae.unclassified.OTU99_000039	3.80	Summer	3.38	0.00
Bacteroidetes.Cytophagia.Cytophagales.Cytophagaceae	3.37	Summer	3.03	0.01
Bacteroidetes.Cytophagia.Cytophagales.Cytophagaceae.Dyadobacter	2.89	Summer	2.61	0.02
Bacteroidetes.Cytophagia.Cytophagales.Cytophagaceae.Dyadobacter.OTU99_004284	2.84	Summer	2.54	0.02
Bacteroidetes.Cytophagia.Cytophagales.Cytophagaceae.Leadbetterella	3.18	Summer	2.83	0.03
Bacteroidetes.Cytophagia.Cytophagales.Cytophagaceae.Leadbetterella.OTU99_000893	2.99	Summer	2.67	0.01
Bacteroidetes.Cytophagia.Order_III	3.30	Summer	2.99	0.00
Bacteroidetes.Cytophagia.Order_III.Unknown_Family	3.30	Summer	2.99	0.00
Bacteroidetes.Cytophagia.Order_III.Unknown_Family.unclassified	3.30	Summer	2.99	0.00
Bacteroidetes.Cytophagia.Order_III.Unknown_Family.unclassified.OTU99_000125	3.29	Summer	2.99	0.00
Bacteroidetes.Flavobacteriia.Flavobacteriales.Cryomorphaceae.Fluviicola	4.10	Summer	3.62	0.00

Taxonomy	LDA	Envrion-ment	Wilcoxon rank-sum test	Kruskal-Wallis
Bacteroidetes.Flavobacteriia.Flavobacteriales.Cryomorphaceae.Fluviicola.OTU99_000093	3.83	Summer	3.43	0.01
Bacteroidetes.Flavobacteriia.Flavobacteriales.Cryomorphaceae.Fluviicola.OTU99_000202	3.58	Summer	3.26	0.00
Bacteroidetes.Flavobacteriia.Flavobacteriales.Cryomorphaceae.Fluviicola.OTU99_001251	3.00	Summer	2.69	0.01
Bacteroidetes.Flavobacteriia.Flavobacteriales.Cryomorphaceae.Owenweeksia.OTU99_000178	3.54	Summer	3.21	0.00
Bacteroidetes.Flavobacteriia.Flavobacteriales.Cryomorphaceae.Owenweeksia.OTU99_000663	3.05	Summer	2.69	0.01
Bacteroidetes.Flavobacteriia.Flavobacteriales.Cryomorphaceae.unclassified.OTU99_000552	2.43	Summer	2.38	0.02
Bacteroidetes.Flavobacteriia.Flavobacteriales.Cryomorphaceae.unclassified.OTU99_000666	3.25	Summer	2.95	0.00
Bacteroidetes.Flavobacteriia.Flavobacteriales.Cryomorphaceae.unclassified.OTU99_000819	2.91	Summer	2.66	0.00
Bacteroidetes.Flavobacteriia.Flavobacteriales.Cryomorphaceae.unclassified.OTU99_001430	2.60	Summer	2.49	0.02
Bacteroidetes.Flavobacteriia.Flavobacteriales.Flavobacteriaceae.Flavobacterium	3.98	Summer	3.50	0.00
Bacteroidetes.Flavobacteriia.Flavobacteriales.Flavobacteriaceae.Flavobacterium.OTU99_000253	3.55	Summer	3.23	0.00
Bacteroidetes.Flavobacteriia.Flavobacteriales.Flavobacteriaceae.Flavobacterium.OTU99_000493	3.03	Summer	2.71	0.04
Bacteroidetes.Flavobacteriia.Flavobacteriales.Flavobacteriaceae.Flavobacterium.OTU99_001351	2.96	Summer	2.66	0.00
Bacteroidetes.Flavobacteriia.Flavobacteriales.Flavobacteriaceae.NS4_marine_group	3.61	Summer	3.02	0.01
Bacteroidetes.Flavobacteriia.Flavobacteriales.Flavobacteriaceae.NS4_marine_group.OTU99_000522	2.84	Summer	2.55	0.04
Bacteroidetes.Flavobacteriia.Flavobacteriales.Flavobacteriaceae.NS5_marine_group.OTU99_000082	3.90	Summer	3.35	0.01
Bacteroidetes.Flavobacteriia.Flavobacteriales.Flavobacteriaceae.unclassified.OTU99_000069	4.03	Summer	3.72	0.00
Bacteroidetes.Flavobacteriia.Flavobacteriales.Flavobacteriaceae.unclassified.OTU99_000175	3.69	Summer	3.40	0.00
Bacteroidetes.Flavobacteriia.Flavobacteriales.Flavobacteriaceae.unclassified.OTU99_000366	3.09	Summer	2.72	0.04
Bacteroidetes.Flavobacteriia.Flavobacteriales.Flavobacteriaceae.unclassified.OTU99_000373	3.42	Summer	3.14	0.02
Bacteroidetes.Flavobacteriia.Flavobacteriales.Flavobacteriaceae.unclassified.OTU99_000874	2.32	Summer	2.27	0.04
Bacteroidetes.Flavobacteriia.Flavobacteriales.Flavobacteriaceae.unclassified.OTU99_000918	2.62	Summer	2.50	0.04
Bacteroidetes.Flavobacteriia.Flavobacteriales.Flavobacteriaceae.unclassified.OTU99_001115	2.85	Summer	2.62	0.04
Bacteroidetes.Flavobacteriia.Flavobacteriales.NS9_marine_group.unclassified.OTU99_000218	3.46	Summer	3.20	0.03
Bacteroidetes.Sphingobacteriia	4.46	Summer	3.83	0.05
Bacteroidetes.Sphingobacteriia.Sphingobacteriales	4.46	Summer	3.83	0.05
Bacteroidetes.Sphingobacteriia.Sphingobacteriales.Chitinophagaceae.Sediminibacterium	2.83	Summer	2.69	0.04
Bacteroidetes.Sphingobacteriia.Sphingobacteriales.NS11_12_marine_group.unclassified.OTU99_000214	3.63	Summer	3.35	0.00
Bacteroidetes.Sphingobacteriia.Sphingobacteriales.NS11_12_marine_group.unclassified.OTU99_000349	3.18	Summer	2.91	0.02
Bacteroidetes.Sphingobacteriia.Sphingobacteriales.Saprospiraceae	4.21	Summer	3.89	0.00
Bacteroidetes.Sphingobacteriia.Sphingobacteriales.Saprospiraceae.Aureispira.OTU99_002051	2.20	Summer	2.24	0.04
Bacteroidetes.Sphingobacteriia.Sphingobacteriales.Saprospiraceae.Candidatus_Aquirestis	3.83	Summer	3.50	0.00
Bacteroidetes.Sphingobacteriia.Sphingobacteriales.Saprospiraceae.Candidatus_Aquirestis.OTU99_000053	3.78	Summer	3.46	0.00
Bacteroidetes.Sphingobacteriia.Sphingobacteriales.Saprospiraceae.Lewinella	3.52	Summer	3.22	0.00
Bacteroidetes.Sphingobacteriia.Sphingobacteriales.Saprospiraceae.Lewinella.OTU99_000507	3.04	Summer	2.80	0.02
Bacteroidetes.Sphingobacteriia.Sphingobacteriales.Saprospiraceae.Lewinella.OTU99_000774	3.14	Summer	2.83	0.04
Bacteroidetes.Sphingobacteriia.Sphingobacteriales.Saprospiraceae.Lewinella.OTU99_002493	2.56	Summer	2.35	0.04
Bacteroidetes.Sphingobacteriia.Sphingobacteriales.Saprospiraceae.unclassified	3.67	Summer	3.38	0.01
Bacteroidetes.Sphingobacteriia.Sphingobacteriales.Saprospiraceae.unclassified.OTU99_000073	3.50	Summer	3.23	0.04
Bacteroidetes.Sphingobacteriia.Sphingobacteriales.Saprospiraceae.uncultured	2.79	Summer	2.50	0.03
bacteroidetes.unclassified	4.39	Summer	4.01	0.00
bacteroidetes.unclassified.unclassified	4.39	Summer	4.01	0.00
bacteroidetes.unclassified.unclassified.unclassified	4.39	Summer	4.01	0.00
bacteroidetes.unclassified.unclassified.unclassified.unclassified	4.39	Summer	4.01	0.00
bacteroidetes.unclassified.unclassified.unclassified.unclassified.OTU99_000025	3.57	Summer	3.28	0.00
bacteroidetes.unclassified.unclassified.unclassified.unclassified.OTU99_000097	3.03	Summer	2.70	0.02
bacteroidetes.unclassified.unclassified.unclassified.unclassified.OTU99_000132	3.71	Summer	3.41	0.00
bacteroidetes.unclassified.unclassified.unclassified.unclassified.OTU99_000232	3.38	Summer	3.02	0.00
bacteroidetes.unclassified.unclassified.unclassified.unclassified.OTU99_000275	3.03	Summer	2.77	0.04
bacteroidetes.unclassified.unclassified.unclassified.unclassified.OTU99_000332	3.25	Summer	2.99	0.00
bacteroidetes.unclassified.unclassified.unclassified.unclassified.OTU99_000536	2.94	Summer	2.67	0.02
Betaproteobacteria.Burkholderiales.Alcaligenaceae.GKS98_freshwater_group.OTU99_000549	2.83	Summer	2.52	0.04
Betaproteobacteria.Burkholderiales.Alcaligenaceae.MWH_UniP1_aquatic_group.OTU99_000241	3.68	Summer	3.29	0.00
Betaproteobacteria.Burkholderiales.Alcaligenaceae.MWH_UniP1_aquatic_group.OTU99_000594	3.07	Summer	2.82	0.00
Betaproteobacteria.Burkholderiales.Alcaligenaceae.unclassified.OTU99_000489	3.19	Summer	2.92	0.00
Betaproteobacteria.Burkholderiales.Alcaligenaceae.unclassified.OTU99_000545	3.47	Summer	3.13	0.01
Betaproteobacteria.Burkholderiales.Comamonadaceae.BAL58_marine_group.OTU99_000303	3.88	Summer	3.46	0.00
Betaproteobacteria.Burkholderiales.Comamonadaceae.Limnohabitans	3.61	Summer	3.23	0.00
Betaproteobacteria.Burkholderiales.Comamonadaceae.Limnohabitans.OTU99_000198	3.31	Summer	3.03	0.00
Betaproteobacteria.Burkholderiales.Comamonadaceae.Limnohabitans.OTU99_013805	2.36	Summer	2.30	0.04
Betaproteobacteria.Burkholderiales.Comamonadaceae.unclassified.OTU99_001414	2.95	Summer	2.61	0.02
Betaproteobacteria.Burkholderiales.Comamonadaceae.uncultured	2.29	Summer	2.27	0.04
Betaproteobacteria.Burkholderiales.unclassified.unclassified.OTU99_008346	2.20	Summer	2.31	0.04
Betaproteobacteria.unclassified.unclassified.unclassified.OTU99_006627	2.31	Summer	2.18	0.04

Taxonomy	LDA	Envrion-ment	Wilcoxon rank-sum test	Kruskal-Wallis
Candidate_division_TM7.unclassified.unclassified.unclassified.unclassified.OTU99_000782	2.54	Summer	2.32	0.04
Cyanobacteria	5.15	Summer	4.57	0.00
Cyanobacteria.Cyanobacteria	5.15	Summer	4.57	0.00
Cyanobacteria.Cyanobacteria.SubsectionI	5.01	Summer	4.40	0.00
Cyanobacteria.Cyanobacteria.SubsectionI.FamilyI	5.01	Summer	4.40	0.00
Cyanobacteria.Cyanobacteria.SubsectionI.FamilyI.Microcystis.OTU99_000179	3.65	Summer	3.28	0.03
Cyanobacteria.Cyanobacteria.SubsectionI.FamilyI.Synechococcus	4.97	Summer	4.42	0.00
Cyanobacteria.Cyanobacteria.SubsectionI.FamilyI.Synechococcus.OTU99_000013	4.74	Summer	4.29	0.01
Cyanobacteria.Cyanobacteria.SubsectionI.FamilyI.Synechococcus.OTU99_000057	4.27	Summer	3.85	0.00
Cyanobacteria.Cyanobacteria.SubsectionI.FamilyI.Synechococcus.OTU99_000212	3.13	Summer	2.80	0.02
Cyanobacteria.Cyanobacteria.SubsectionI.FamilyI.Synechococcus.OTU99_000279	3.12	Summer	2.78	0.01
Cyanobacteria.Cyanobacteria.SubsectionIII	2.89	Summer	2.74	0.02
Cyanobacteria.Cyanobacteria.SubsectionIII.FamilyI	2.89	Summer	2.73	0.02
Cyanobacteria.Cyanobacteria.SubsectionIII.FamilyI.unclassified	2.89	Summer	2.65	0.02
Cyanobacteria.Cyanobacteria.SubsectionIV.FamilyI.Nodularia	3.11	Summer	2.85	0.00
Cyanobacteria.Cyanobacteria.SubsectionIV.FamilyI.Nodularia.OTU99_000321	3.06	Summer	2.81	0.01
Cyanobacteria.Cyanobacteria.SubsectionIV.FamilyI.unclassified.OTU99_000015	4.42	Summer	4.15	0.00
Deltaproteobacteria.Bdellovibrionales.Bacteriovoracaceae	3.28	Summer	2.95	0.01
Deltaproteobacteria.Bdellovibrionales.Bacteriovoracaceae.Peredibacter	2.72	Summer	2.54	0.00
Deltaproteobacteria.Bdellovibrionales.Bacteriovoracaceae.unclassified	3.12	Summer	2.79	0.01
Deltaproteobacteria.Bdellovibrionales.Bacteriovoracaceae.unclassified.OTU99_000891	2.99	Summer	2.77	0.02
Deltaproteobacteria.Myxococcales.0319_6G20.unclassified.OTU99_001506	2.42	Summer	2.34	0.04
Deltaproteobacteria.Myxococcales.0319_6G20.unclassified.OTU99_003923	2.41	Summer	2.70	0.04
Gammaproteobacteria.Alteromonadales.Alteromonadaceae.Alteromonas	2.58	Summer	2.48	0.04
Gammaproteobacteria.Alteromonadales.Alteromonadaceae.Halioglobus	3.91	Summer	3.58	0.00
Gammaproteobacteria.Alteromonadales.Alteromonadaceae.Halioglobus.OTU99_000060	3.90	Summer	3.58	0.00
Gammaproteobacteria.Alteromonadales.Alteromonadaceae.OM60_NOR5__clade.OTU99_000374	3.53	Summer	3.27	0.04
Gammaproteobacteria.Alteromonadales.Alteromonadaceae.OM60_NOR5__clade.OTU99_000662	3.05	Summer	2.65	0.03
Gammaproteobacteria.Alteromonadales.Alteromonadaceae.SAR92_clade.OTU99_000338	3.32	Summer	3.01	0.04
Gammaproteobacteria.Oceanospirillales.Halomonadaceae.Halomonas.OTU99_001123	2.92	Summer	2.84	0.04
Gammaproteobacteria.Oceanospirillales.Litoricolaceae	3.12	Summer	2.85	0.04
Gammaproteobacteria.Oceanospirillales.Litoricolaceae.Litoricola	3.12	Summer	2.83	0.04
Gammaproteobacteria.Oceanospirillales.Litoricolaceae.Litoricola.OTU99_000846	3.12	Summer	2.84	0.04
Gammaproteobacteria.Oceanospirillales.Oceanospirillaceae.Pseudospirillum.OTU99_000789	3.07	Summer	2.77	0.02
Gammaproteobacteria.Oceanospirillales.Oceanospirillaceae.Pseudospirillum.OTU99_000827	3.09	Summer	2.79	0.00
Gammaproteobacteria.Pseudomonadales.Pseudomonadaceae.Pseudomonas.OTU99_000558	2.49	Summer	2.38	0.04
Gammaproteobacteria.Thiotrichales	3.50	Summer	3.05	0.03
Gammaproteobacteria.Thiotrichales.Piscirickettsiaceae	3.47	Summer	3.11	0.01
Gammaproteobacteria.Thiotrichales.Piscirickettsiaceae.unclassified	3.45	Summer	3.15	0.00
Gammaproteobacteria.Thiotrichales.Piscirickettsiaceae.unclassified.OTU99_000485	3.39	Summer	3.09	0.00
Gammaproteobacteria.unclassified.unclassified.unclassified.OTU99_000243	3.89	Summer	3.56	0.00
Planctomycetes.OM190	3.27	Summer	2.82	0.03
Planctomycetes.OM190.unclassified	3.27	Summer	2.82	0.03
Planctomycetes.OM190.unclassified.unclassified	3.27	Summer	2.82	0.03
Planctomycetes.OM190.unclassified.unclassified.unclassified	3.27	Summer	2.82	0.03
Planctomycetes.OM190.unclassified.unclassified.unclassified.OTU99_000437	2.82	Summer	2.56	0.00
Planctomycetes.OM190.unclassified.unclassified.unclassified.OTU99_000919	2.90	Summer	2.59	0.00
Planctomycetes.Phycisphaerae.Phycisphaerales.Phycisphaeraceae.CL500_3.OTU99_000912	2.35	Summer	2.20	0.02
Planctomycetes.Phycisphaerae.Phycisphaerales.Phycisphaeraceae.SM1A02	2.96	Summer	2.66	0.01
Planctomycetes.Phycisphaerae.Phycisphaerales.Phycisphaeraceae.SM1A02.OTU99_000224	2.63	Summer	2.39	0.01
Planctomycetes.Planctomycetacia.Planctomycetales.Planctomycetaceae.Pirellula	2.80	Summer	2.53	0.04
Planctomycetes.Planctomycetacia.Planctomycetales.Planctomycetaceae.Pirellula.OTU99_000973	2.73	Summer	2.69	0.04
Planctomycetes.Planctomycetacia.Planctomycetales.Planctomycetaceae.Planctomyces.OTU99_000100	2.85	Summer	2.60	0.02
SC3_20.unclassified.unclassified.unclassified.OTU99_001722	2.55	Summer	2.34	0.02
unclassified.unclassified.unclassified.unclassified.OTU99_001208	2.97	Summer	2.72	0.00
unclassified.unclassified.unclassified.unclassified.OTU99_001758	3.01	Summer	2.69	0.00
unclassified.unclassified.unclassified.unclassified.OTU99_004020	2.70	Summer	2.47	0.01
unclassified.unclassified.unclassified.unclassified.unclassified.OTU99_000061	4.12	Summer	3.70	0.00
unclassified.unclassified.unclassified.unclassified.unclassified.OTU99_000454	3.00	Summer	2.73	0.02
unclassified.unclassified.unclassified.unclassified.unclassified.OTU99_001646	2.83	Summer	2.62	0.02
Verrucomicrobia	5.19	Summer	4.64	0.00
Verrucomicrobia.OPB35_soil_group.unclassified.unclassified.unclassified.OTU99_000749	2.97	Summer	2.70	0.00
Verrucomicrobia.Opitutae	4.02	Summer	3.57	0.01
Verrucomicrobia.Opitutae.Puniceicoccales	3.76	Summer	3.41	0.02

Taxonomy	LDA	Envrion-ment	Wilcoxon rank-sum test	Kruskal-Wallis
Verrucomicrobia.Opitutae.Puniceicoccales.Puniceicoccaceae	3.76	Summer	3.41	0.02
Verrucomicrobia.Opitutae.Puniceicoccales.Puniceicoccaceae.Lentimonas.OTU99_001863	2.84	Summer	2.56	0.04
Verrucomicrobia.Opitutae.Puniceicoccales.Puniceicoccaceae.unclassified	3.68	Summer	3.38	0.00
Verrucomicrobia.Opitutae.Puniceicoccales.Puniceicoccaceae.unclassified.OTU99_000129	3.56	Summer	3.26	0.00
Verrucomicrobia.Spartobacteria	5.10	Summer	4.58	0.01
Verrucomicrobia.Spartobacteria.Chthoniobacterales	5.10	Summer	4.58	0.01
Verrucomicrobia.Spartobacteria.Chthoniobacterales.unclassified	5.08	Summer	4.60	0.01
Verrucomicrobia.Spartobacteria.Chthoniobacterales.unclassified.unclassified	5.08	Summer	4.60	0.01
Verrucomicrobia.Spartobacteria.Chthoniobacterales.unclassified.unclassified.OTU99_000005	5.06	Summer	4.66	0.00
Verrucomicrobia.Verrucomicrobiae.Verrucomicrobiales.DEV007.unclassified.OTU99_000124	2.21	Summer	2.18	0.04
Verrucomicrobia.Verrucomicrobiae.Verrucomicrobiales.Verrucomicrobiaceae	4.13	Summer	3.66	0.00
Verrucomicrobia.Verrucomicrobiae.Verrucomicrobiales.Verrucomicrobiaceae.Roseibacillus	4.00	Summer	3.50	0.00
Verrucomicrobia.Verrucomicrobiae.Verrucomicrobiales.Verrucomicrobiaceae.Roseibacillus.OTU99_000193	3.93	Summer	3.61	0.00
Verrucomicrobia.Verrucomicrobiae.Verrucomicrobiales.Verrucomicrobiaceae.unclassified	3.45	Summer	3.08	0.01
Verrucomicrobia.Verrucomicrobiae.Verrucomicrobiales.Verrucomicrobiaceae.unclassified.OTU99_000049	2.84	Summer	2.58	0.02
Verrucomicrobia.Verrucomicrobiae.Verrucomicrobiales.Verrucomicrobiaceae.unclassified.OTU99_000432	2.99	Summer	2.71	0.04
Verrucomicrobia.Verrucomicrobiae.Verrucomicrobiales.Verrucomicrobiaceae.uncultured	2.32	Summer	2.38	0.04

Table S3. Abundant (>1% of total reads) operational taxonomic units (OTUs) and phyla/classes with significant (α = 0.05) higher relative abundance in one of the salinity realms (indicated in bold). OTUs that were not phylogenetically assigned are named as "unclassified" and the next formally described phylogenetic level is given. Shown is the average relative abundance in the marine and mesohaline realms (in %) and standard deviation in brackets based on the congruent dataset.

Marine	Mesohaline	Identified OTU/Class/Phylum
**3.1 (4.4)**	0 (0)	SAR86 OTU-42 (*Gammaproteobacteria*)
**3.0 (2.6)**	0 (0.1)	"*Candidatus* Planktomarina" OTU-52 (*Alphaproteobacteria*)
**2.7 (3.8)**	0 (0)	"*Roseobacter* OCT lineage" OTU-58 (*Alphaproteobacteria*)
**2.5 (3.5)**	0 (0)	*Synechococcus* OTU-10 (*Cyanobacteria*)
**2.1 (3.9)**	0 (0)	"Unclassified Oceanospirillales" OTU-23(*Gammaproteobacteria*)
**1.9 (2.3)**	0 (0.1)	SAR116 OTU-168 (*Alphaproteobacteria*)
**1.6 (1.4)**	0.6 (0.9)	SAR11-II OTU-200 (*Alphaproteobacteria*)
**1.4 (1.7)**	0 (0)	SAR116 OTU-150 (*Alphaproteobacteria*)
**1.3 (2.1)**	0 (0)	SAR11-II OTU-18 (*Alphaproteobacteria*)
**1.3 (1.4)**	0 (0)	"Unclassified OM43" OTU-92*(Betaproteobacteria)*
**1.1 (1.8)**	0 (0)	*Synechococcus* OTU-41 (*Cyanobacteria*)
**1.1 (1.2)**	0 (0)	"Unclassified NOR5/OM60" OTU-105 (*Gammaproteobacteria*)
**1.1 (1.8)**	0 (0)	"Unclassified Alteromonadales" OTU-131 (*Gammaproteobacteria*)
**1.1 (1.5)**	0 (0)	SAR86 OTU-189 (*Gammaproteobacteria*)
**1.0 (1.7)**	0 (0)	"*Candidatus* Actinomarina" OTU-50 (*Actinobacteria*)
0.3 (0.7)	**9.1 (8.4)**	"Unclassified Spartobacteria" OTU-5 (*Verrucomicrobia*)
0.7 (1.8)	**4.5 (3.6)**	*Synechococcus* OTU-13 (*Cyanobacteria*)
0.6 (0.8)	**4.1 (3.5)**	"SAR11-IIIa" OTU-14 (*Alphaproteobacteria*)
0.6 (1.6)	**2.1 (2.1)**	"Unclassified *Rhodobacter*" OTU- 16 (*Alphaproteobacteria*)
0 (0.1)	**2.0 (2.1)**	hgcI-clade OTU-36 (*Actinobacteria*)
0.4 (1.1)	**1.4 (1.8)**	*Synechococcus* OTU-57 (*Cyanobacteria*)
0 (0)	**1.3 (1.9)**	*Synechococcus* OTU-64 (*Cyanobacteria*)
0.1 (0.1)	**1.2 (2.2)**	"Unclassified Corynebacteriales" OTU-35 (*Actinobacteria*)
0.5 (1.0)	**1.2 (1.4)**	"Unclassified Flavobacteriaceae" OTU-31 (*Bacteroidetes*)
0 (0)	**1.1 (1.3)**	hgcI-clade OTU-21 (*Actinobacteria*)
**18.7**	4.1	*Gammaproteobacteria*
**31.0**	17.3	*Alphaproteobacteria*
6.5	**16.3**	*Actinobacteria*
3.2	**13.7**	*Verrucomicrobia*
6.0	**12.1**	*Cyanobacteria*
2.0	**5.2**	*Planctomycetes*
2.9	**5.4**	*Betaproteobacteria*

Table S3. Abundant (>1% of total reads) operational taxonomic units (OTUs) and phyla/classes with significant (α = 0.05) higher relative abundance in winter and summer (indicated in bold). OTUs that could not be taxonomically assigned are named as "unclassified" and the next formally described taxonomic level is given. Shown is the average relative abundance from the summer and winter samples (in %) and standard deviation in brackets based on the congruent dataset.

**Summer**	**Winter**	**Identified OTU/ Class/Phylum**
**11.5 (9.3)**	2.6 (3.4)	"Unclassified Spartobacteria" OTU-5 (*Verrucomicrobia*)
**5.5 (3.6)**	1.7 (2.6)	*Synechococcus* OTU-13 (*Cyanobacteria*)
**2.6 (5.1)**	0 (0)	Family I OTU-15 (*Cyanobacteria*)
**1.9 (2.1)**	0.4 (0.8)	*Synechococcus* OTU-57 (*Cyanobacteria*)
**1.4 (1.0)**	0.1 (0.1)	"hgcI-clade" OTU-45 (*Actinobacteria*)
**1.2 (1.4)**	0.1 (0.3)	"Unclassified Microbacteriaceae" OTU-167 (*Actinobacteria*)
**1.2 (1.3)**	0 (0)	"Unclassified Acidimicrobiales" OTU-162 (*Actinobacteria*)
**1.1 (1.1)**	0 (0.1)	"Unclassified *Rhodobacter*" OTU-136 (*Alphaproteobacteria*)
**1.1 (1.6)**	0 (0)	"Unclassified Flavobacteriaceae" OTU-69 (*Bacteroidetes*)
0.6 (1.3)	**2.9 (2.1)**	"Unclassified *Rhodobacter*" OTU-16 (*Alphaproteobacteria*)
0.2 (0.2)	**1.8 (1.5)**	"Unclassified Flavobacteriaceae" OTU-31 (*Bacteroidetes*)
0.1 (0.3)	**1.8 (2.6)**	"Unclassified Corynebacteriales" OTU-35 (*Actinobacteria*)
0.5 (0.8)	**1.1 (1.3)**	SAR11-II OTU-200 (*Alphaproteobacteria*)
0 (0)	**1.3 (1.6)**	"Unclassified Spartobacteria" OTU-101 (*Verrucomicrobia*)
0 (0)	**1.3 (1.3)**	"Unclassified Flavobacteriaceae" OTU-59 (*Bacteroidetes*)
**19.7**	16.9	*Bacteroidetes*
**15.6**	6.9	*Verrucomicrobia*
**14.2**	7.2	*Cyanobacteria*
13.3	**14.8**	*Actinobacteria*
2.1	**7.0**	*Planctomycetes*
4.2	**5.6**	*Betaproteobacteria*
0.4	**1.2**	*Deltaproteobacteria*
0	**1.5**	*Chloroflexi*

Table S4. Long branches excluded from the analysis of the phylogenetic signal

OTU	Rank taxonomy
OTU99_001764	Bacteria;
OTU99_013216	Bacteria;
OTU99_000223	Bacteria;
OTU99_025270	Bacteria;
OTU99_013860	Bacteria;
OTU99_002692	Bacteria;
OTU99_007532	Bacteria;
OTU99_028722	Bacteria;
OTU99_002983	Bacteria;
OTU99_016549	Bacteria;
OTU99_015101	Bacteria;
OTU99_005941	Bacteria;
OTU99_001724	Bacteria;
OTU99_005607	Bacteria;
OTU99_000852	Bacteria;
OTU99_027825	Bacteria;
OTU99_019400	Bacteria;
OTU99_031318	Bacteria;
OTU99_000454	Bacteria;
OTU99_020305	Bacteria;
OTU99_019877	Bacteria;
OTU99_007796	Bacteria;
OTU99_016058	Bacteria;
OTU99_000392	Bacteria;
OTU99_004955	Bacteria;
OTU99_005484	Bacteria;
OTU99_027370	Bacteria;
OTU99_005253	Bacteria;
OTU99_018827	Bacteria;
OTU99_013334	Bacteria;
OTU99_004480	Bacteria;Acidobacteria;Acidobacteria;Subgroup
OTU99_006125	Bacteria;Acidobacteria;Acidobacteria;Subgroup
OTU99_021666	Bacteria;Actinobacteria;
OTU99_007537	Bacteria;Actinobacteria;
OTU99_007729	Bacteria;Actinobacteria;Actinobacteria;Frankiales;Sporichthyaceae;hgcI
OTU99_014716	Bacteria;Actinobacteria;Actinobacteria;Frankiales;Sporichthyaceae;hgcI
OTU99_017570	Bacteria;Actinobacteria;Actinobacteria;Frankiales;Sporichthyaceae;hgcI
OTU99_000045	Bacteria;Actinobacteria;Actinobacteria;Frankiales;Sporichthyaceae;hgcI
OTU99_008206	Bacteria;Actinobacteria;Actinobacteria;Frankiales;Sporichthyaceae;hgcI
OTU99_019921	Bacteria;Actinobacteria;Actinobacteria;Frankiales;Sporichthyaceae;hgcI
OTU99_004258	Bacteria;Actinobacteria;Actinobacteria;Frankiales;Sporichthyaceae;hgcI
OTU99_000036	Bacteria;Actinobacteria;Actinobacteria;Frankiales;Sporichthyaceae;hgcI
OTU99_015530	Bacteria;Actinobacteria;Actinobacteria;Frankiales;Sporichthyaceae;hgcI
OTU99_007030	Bacteria;Actinobacteria;Actinobacteria;Frankiales;Sporichthyaceae;hgcI
OTU99_006623	Bacteria;Actinobacteria;Actinobacteria;Frankiales;Sporichthyaceae;hgcI
OTU99_007392	Bacteria;Actinobacteria;Actinobacteria;Frankiales;Sporichthyaceae;hgcI
OTU99_027819	Bacteria;Actinobacteria;Actinobacteria;Frankiales;Sporichthyaceae;hgcI
OTU99_000606	Bacteria;Actinobacteria;Actinobacteria;Frankiales;Sporichthyaceae;hgcI
OTU99_000021	Bacteria;Actinobacteria;Actinobacteria;Frankiales;Sporichthyaceae;hgcI
OTU99_007866	Bacteria;Actinobacteria;Actinobacteria;Frankiales;Sporichthyaceae;hgcI
OTU99_014933	Bacteria;Actinobacteria;Actinobacteria;Frankiales;Sporichthyaceae;hgcI
OTU99_005101	Bacteria;Actinobacteria;Actinobacteria;Frankiales;Sporichthyaceae;hgcI
OTU99_001510	Bacteria;Actinobacteria;Actinobacteria;Frankiales;Sporichthyaceae;hgcI
OTU99_004592	Bacteria;Actinobacteria;Actinobacteria;Frankiales;Sporichthyaceae;hgcI
OTU99_005161	Bacteria;Actinobacteria;Actinobacteria;Frankiales;Sporichthyaceae;hgcI
OTU99_017166	Bacteria;Actinobacteria;Actinobacteria;Frankiales;Sporichthyaceae;hgcI
OTU99_007915	Bacteria;Actinobacteria;Actinobacteria;Frankiales;Sporichthyaceae;hgcI
OTU99_032626	Bacteria;Actinobacteria;Actinobacteria;Frankiales;Sporichthyaceae;hgcI
OTU99_023849	Bacteria;Bacteroidetes;
OTU99_025350	Bacteria;Bacteroidetes;
OTU99_000048	Bacteria;Bacteroidetes;
OTU99_002216	Bacteria;Bacteroidetes;
OTU99_000132	Bacteria;Bacteroidetes;
OTU99_015898	Bacteria;Bacteroidetes;
OTU99_031221	Bacteria;Bacteroidetes;
OTU99_032685	Bacteria;Bacteroidetes;
OTU99_027456	Bacteria;Bacteroidetes;
OTU99_015701	Bacteria;Bacteroidetes;
OTU99_006459	Bacteria;Bacteroidetes;
OTU99_019708	Bacteria;Bacteroidetes;
OTU99_001711	Bacteria;Bacteroidetes;
OTU99_028259	Bacteria;Bacteroidetes;Flavobacteriia;Flavobacteriales;
OTU99_000518	Bacteria;Bacteroidetes;Flavobacteriia;Flavobacteriales;
OTU99_004774	Bacteria;Bacteroidetes;Flavobacteriia;Flavobacteriales;
OTU99_004970	Bacteria;Bacteroidetes;Flavobacteriia;Flavobacteriales;
OTU99_006239	Bacteria;Bacteroidetes;Flavobacteriia;Flavobacteriales;
OTU99_008239	Bacteria;Bacteroidetes;Flavobacteriia;Flavobacteriales;
OTU99_005956	Bacteria;Bacteroidetes;Flavobacteriia;Flavobacteriales;
OTU99_017229	Bacteria;Bacteroidetes;Flavobacteriia;Flavobacteriales;
OTU99_000204	Bacteria;Bacteroidetes;Flavobacteriia;Flavobacteriales;
OTU99_008828	Bacteria;Bacteroidetes;Flavobacteriia;Flavobacteriales;

OTU	Rank taxonomy
OTU99_032704	Bacteria;Bacteroidetes;Flavobacteriia;Flavobacteriales;
OTU99_021428	Bacteria;Bacteroidetes;Flavobacteriia;Flavobacteriales;
OTU99_001228	Bacteria;Bacteroidetes;Flavobacteriia;Flavobacteriales;
OTU99_004888	Bacteria;Bacteroidetes;Flavobacteriia;Flavobacteriales;
OTU99_000862	Bacteria;Bacteroidetes;Flavobacteriia;Flavobacteriales;
OTU99_020717	Bacteria;Bacteroidetes;Flavobacteriia;Flavobacteriales;
OTU99_000513	Bacteria;Bacteroidetes;Flavobacteriia;Flavobacteriales;
OTU99_002892	Bacteria;Bacteroidetes;Flavobacteriia;Flavobacteriales;
OTU99_006649	Bacteria;Bacteroidetes;Flavobacteriia;Flavobacteriales;Cryomorphaceae;Fluviicola;
OTU99_001070	Bacteria;Bacteroidetes;Flavobacteriia;Flavobacteriales;Cryomorphaceae;Fluviicola;
OTU99_000245	Bacteria;Bacteroidetes;Flavobacteriia;Flavobacteriales;Cryomorphaceae;Fluviicola;
OTU99_005743	Bacteria;Bacteroidetes;Flavobacteriia;Flavobacteriales;Cryomorphaceae;Owenweeksia;
OTU99_002658	Bacteria;Bacteroidetes;Flavobacteriia;Flavobacteriales;Cryomorphaceae;Owenweeksia;
OTU99_026788	Bacteria;Bacteroidetes;Flavobacteriia;Flavobacteriales;Cryomorphaceae;Owenweeksia;
OTU99_001916	Bacteria;Bacteroidetes;Flavobacteriia;Flavobacteriales;Cryomorphaceae;Owenweeksia;
OTU99_001253	Bacteria;Bacteroidetes;Flavobacteriia;Flavobacteriales;Cryomorphaceae;Owenweeksia;
OTU99_007593	Bacteria;Bacteroidetes;Flavobacteriia;Flavobacteriales;Cryomorphaceae;Owenweeksia;
OTU99_000145	Bacteria;Bacteroidetes;Flavobacteriia;Flavobacteriales;Cryomorphaceae;Owenweeksia;
OTU99_003428	Bacteria;Bacteroidetes;Flavobacteriia;Flavobacteriales;Flavobacteriaceae;
OTU99_000577	Bacteria;Bacteroidetes;Flavobacteriia;Flavobacteriales;Flavobacteriaceae;
OTU99_019675	Bacteria;Bacteroidetes;Flavobacteriia;Flavobacteriales;Flavobacteriaceae;
OTU99_019714	Bacteria;Bacteroidetes;Flavobacteriia;Flavobacteriales;Flavobacteriaceae;
OTU99_004567	Bacteria;Bacteroidetes;Flavobacteriia;Flavobacteriales;Flavobacteriaceae;
OTU99_000366	Bacteria;Bacteroidetes;Flavobacteriia;Flavobacteriales;Flavobacteriaceae;
OTU99_026282	Bacteria;Bacteroidetes;Flavobacteriia;Flavobacteriales;Flavobacteriaceae;
OTU99_016591	Bacteria;Bacteroidetes;Flavobacteriia;Flavobacteriales;Flavobacteriaceae;
OTU99_023146	Bacteria;Bacteroidetes;Flavobacteriia;Flavobacteriales;Flavobacteriaceae;
OTU99_006711	Bacteria;Bacteroidetes;Flavobacteriia;Flavobacteriales;Flavobacteriaceae;
OTU99_013468	Bacteria;Bacteroidetes;Flavobacteriia;Flavobacteriales;Flavobacteriaceae;
OTU99_000182	Bacteria;Bacteroidetes;Flavobacteriia;Flavobacteriales;Flavobacteriaceae;
OTU99_009058	Bacteria;Bacteroidetes;Flavobacteriia;Flavobacteriales;Flavobacteriaceae;
OTU99_008299	Bacteria;Bacteroidetes;Flavobacteriia;Flavobacteriales;Flavobacteriaceae;
OTU99_019449	Bacteria;Bacteroidetes;Flavobacteriia;Flavobacteriales;Flavobacteriaceae;
OTU99_032156	Bacteria;Bacteroidetes;Flavobacteriia;Flavobacteriales;Flavobacteriaceae;
OTU99_017244	Bacteria;Bacteroidetes;Flavobacteriia;Flavobacteriales;Flavobacteriaceae;
OTU99_001942	Bacteria;Bacteroidetes;Flavobacteriia;Flavobacteriales;Flavobacteriaceae;
OTU99_000939	Bacteria;Bacteroidetes;Flavobacteriia;Flavobacteriales;Flavobacteriaceae;
OTU99_001579	Bacteria;Bacteroidetes;Flavobacteriia;Flavobacteriales;Flavobacteriaceae;
OTU99_006567	Bacteria;Bacteroidetes;Flavobacteriia;Flavobacteriales;Flavobacteriaceae;
OTU99_021444	Bacteria;Bacteroidetes;Flavobacteriia;Flavobacteriales;Flavobacteriaceae;
OTU99_026592	Bacteria;Bacteroidetes;Flavobacteriia;Flavobacteriales;Flavobacteriaceae;
OTU99_006919	Bacteria;Bacteroidetes;Flavobacteriia;Flavobacteriales;Flavobacteriaceae;
OTU99_006269	Bacteria;Bacteroidetes;Flavobacteriia;Flavobacteriales;Flavobacteriaceae;
OTU99_019790	Bacteria;Bacteroidetes;Flavobacteriia;Flavobacteriales;Flavobacteriaceae;
OTU99_007082	Bacteria;Bacteroidetes;Flavobacteriia;Flavobacteriales;Flavobacteriaceae;
OTU99_005499	Bacteria;Bacteroidetes;Flavobacteriia;Flavobacteriales;Flavobacteriaceae;
OTU99_032171	Bacteria;Bacteroidetes;Flavobacteriia;Flavobacteriales;Flavobacteriaceae;
OTU99_003665	Bacteria;Bacteroidetes;Flavobacteriia;Flavobacteriales;Flavobacteriaceae;
OTU99_000062	Bacteria;Bacteroidetes;Flavobacteriia;Flavobacteriales;Flavobacteriaceae;
OTU99_021748	Bacteria;Bacteroidetes;Flavobacteriia;Flavobacteriales;Flavobacteriaceae;
OTU99_001832	Bacteria;Bacteroidetes;Flavobacteriia;Flavobacteriales;Flavobacteriaceae;
OTU99_000231	Bacteria;Bacteroidetes;Flavobacteriia;Flavobacteriales;Flavobacteriaceae;
OTU99_016578	Bacteria;Bacteroidetes;Flavobacteriia;Flavobacteriales;Flavobacteriaceae;
OTU99_001500	Bacteria;Bacteroidetes;Flavobacteriia;Flavobacteriales;Flavobacteriaceae;
OTU99_001149	Bacteria;Bacteroidetes;Flavobacteriia;Flavobacteriales;Flavobacteriaceae;
OTU99_004443	Bacteria;Bacteroidetes;Flavobacteriia;Flavobacteriales;Flavobacteriaceae;
OTU99_000477	Bacteria;Bacteroidetes;Flavobacteriia;Flavobacteriales;Flavobacteriaceae;
OTU99_004000	Bacteria;Bacteroidetes;Flavobacteriia;Flavobacteriales;Flavobacteriaceae;
OTU99_000768	Bacteria;Bacteroidetes;Flavobacteriia;Flavobacteriales;Flavobacteriaceae;
OTU99_000301	Bacteria;Bacteroidetes;Flavobacteriia;Flavobacteriales;Flavobacteriaceae;
OTU99_012723	Bacteria;Bacteroidetes;Flavobacteriia;Flavobacteriales;Flavobacteriaceae;
OTU99_011731	Bacteria;Bacteroidetes;Flavobacteriia;Flavobacteriales;Flavobacteriaceae;
OTU99_033138	Bacteria;Bacteroidetes;Flavobacteriia;Flavobacteriales;Flavobacteriaceae;
OTU99_002947	Bacteria;Bacteroidetes;Flavobacteriia;Flavobacteriales;Flavobacteriaceae;
OTU99_026277	Bacteria;Bacteroidetes;Flavobacteriia;Flavobacteriales;Flavobacteriaceae;
OTU99_025232	Bacteria;Bacteroidetes;Flavobacteriia;Flavobacteriales;Flavobacteriaceae;
OTU99_028522	Bacteria;Bacteroidetes;Flavobacteriia;Flavobacteriales;Flavobacteriaceae;
OTU99_010610	Bacteria;Bacteroidetes;Flavobacteriia;Flavobacteriales;Flavobacteriaceae;
OTU99_000874	Bacteria;Bacteroidetes;Flavobacteriia;Flavobacteriales;Flavobacteriaceae;
OTU99_000490	Bacteria;Bacteroidetes;Flavobacteriia;Flavobacteriales;Flavobacteriaceae;
OTU99_005089	Bacteria;Bacteroidetes;Flavobacteriia;Flavobacteriales;Flavobacteriaceae;
OTU99_023980	Bacteria;Bacteroidetes;Flavobacteriia;Flavobacteriales;Flavobacteriaceae;
OTU99_003433	Bacteria;Bacteroidetes;Flavobacteriia;Flavobacteriales;Flavobacteriaceae;
OTU99_009075	Bacteria;Bacteroidetes;Flavobacteriia;Flavobacteriales;Flavobacteriaceae;
OTU99_000161	Bacteria;Bacteroidetes;Flavobacteriia;Flavobacteriales;Flavobacteriaceae;
OTU99_032022	Bacteria;Bacteroidetes;Flavobacteriia;Flavobacteriales;Flavobacteriaceae;
OTU99_006840	Bacteria;Bacteroidetes;Flavobacteriia;Flavobacteriales;Flavobacteriaceae;
OTU99_007019	Bacteria;Bacteroidetes;Flavobacteriia;Flavobacteriales;Flavobacteriaceae;
OTU99_006118	Bacteria;Bacteroidetes;Flavobacteriia;Flavobacteriales;Flavobacteriaceae;
OTU99_030047	Bacteria;Bacteroidetes;Flavobacteriia;Flavobacteriales;Flavobacteriaceae;
OTU99_000011	Bacteria;Bacteroidetes;Flavobacteriia;Flavobacteriales;Flavobacteriaceae;
OTU99_006098	Bacteria;Bacteroidetes;Flavobacteriia;Flavobacteriales;Flavobacteriaceae;
OTU99_003794	Bacteria;Bacteroidetes;Flavobacteriia;Flavobacteriales;Flavobacteriaceae;

OTU	Rank taxonomy
OTU99_000394	Bacteria;Bacteroidetes;Flavobacteriia;Flavobacteriales;Flavobacteriaceae;
OTU99_009528	Bacteria;Bacteroidetes;Flavobacteriia;Flavobacteriales;Flavobacteriaceae;
OTU99_013267	Bacteria;Bacteroidetes;Flavobacteriia;Flavobacteriales;Flavobacteriaceae;
OTU99_000841	Bacteria;Bacteroidetes;Flavobacteriia;Flavobacteriales;Flavobacteriaceae;
OTU99_018542	Bacteria;Bacteroidetes;Flavobacteriia;Flavobacteriales;Flavobacteriaceae;
OTU99_017290	Bacteria;Bacteroidetes;Flavobacteriia;Flavobacteriales;Flavobacteriaceae;
OTU99_025698	Bacteria;Bacteroidetes;Flavobacteriia;Flavobacteriales;Flavobacteriaceae;
OTU99_006746	Bacteria;Bacteroidetes;Flavobacteriia;Flavobacteriales;Flavobacteriaceae;
OTU99_006175	Bacteria;Bacteroidetes;Flavobacteriia;Flavobacteriales;Flavobacteriaceae;
OTU99_021692	Bacteria;Bacteroidetes;Flavobacteriia;Flavobacteriales;Flavobacteriaceae;
OTU99_000103	Bacteria;Bacteroidetes;Flavobacteriia;Flavobacteriales;Flavobacteriaceae;
OTU99_027755	Bacteria;Bacteroidetes;Flavobacteriia;Flavobacteriales;Flavobacteriaceae;
OTU99_000515	Bacteria;Bacteroidetes;Flavobacteriia;Flavobacteriales;Flavobacteriaceae;
OTU99_015296	Bacteria;Bacteroidetes;Flavobacteriia;Flavobacteriales;Flavobacteriaceae;
OTU99_003908	Bacteria;Bacteroidetes;Flavobacteriia;Flavobacteriales;Flavobacteriaceae;
OTU99_005969	Bacteria;Bacteroidetes;Flavobacteriia;Flavobacteriales;Flavobacteriaceae;
OTU99_026021	Bacteria;Bacteroidetes;Flavobacteriia;Flavobacteriales;Flavobacteriaceae;
OTU99_001097	Bacteria;Bacteroidetes;Flavobacteriia;Flavobacteriales;Flavobacteriaceae;
OTU99_025904	Bacteria;Bacteroidetes;Flavobacteriia;Flavobacteriales;Flavobacteriaceae;
OTU99_000845	Bacteria;Bacteroidetes;Flavobacteriia;Flavobacteriales;Flavobacteriaceae;
OTU99_007758	Bacteria;Bacteroidetes;Flavobacteriia;Flavobacteriales;Flavobacteriaceae;
OTU99_009469	Bacteria;Bacteroidetes;Flavobacteriia;Flavobacteriales;Flavobacteriaceae;
OTU99_012351	Bacteria;Bacteroidetes;Flavobacteriia;Flavobacteriales;Flavobacteriaceae;
OTU99_000069	Bacteria;Bacteroidetes;Flavobacteriia;Flavobacteriales;Flavobacteriaceae;
OTU99_026260	Bacteria;Bacteroidetes;Flavobacteriia;Flavobacteriales;Flavobacteriaceae;
OTU99_004633	Bacteria;Bacteroidetes;Flavobacteriia;Flavobacteriales;Flavobacteriaceae;
OTU99_023970	Bacteria;Bacteroidetes;Flavobacteriia;Flavobacteriales;Flavobacteriaceae;
OTU99_029124	Bacteria;Bacteroidetes;Flavobacteriia;Flavobacteriales;Flavobacteriaceae;
OTU99_019742	Bacteria;Bacteroidetes;Flavobacteriia;Flavobacteriales;Flavobacteriaceae;
OTU99_000031	Bacteria;Bacteroidetes;Flavobacteriia;Flavobacteriales;Flavobacteriaceae;
OTU99_008297	Bacteria;Bacteroidetes;Flavobacteriia;Flavobacteriales;Flavobacteriaceae;
OTU99_025393	Bacteria;Bacteroidetes;Flavobacteriia;Flavobacteriales;Flavobacteriaceae;
OTU99_007165	Bacteria;Bacteroidetes;Flavobacteriia;Flavobacteriales;Flavobacteriaceae;
OTU99_003043	Bacteria;Bacteroidetes;Flavobacteriia;Flavobacteriales;Flavobacteriaceae;
OTU99_026247	Bacteria;Bacteroidetes;Flavobacteriia;Flavobacteriales;Flavobacteriaceae;
OTU99_010062	Bacteria;Bacteroidetes;Flavobacteriia;Flavobacteriales;Flavobacteriaceae;
OTU99_025624	Bacteria;Bacteroidetes;Flavobacteriia;Flavobacteriales;Flavobacteriaceae;
OTU99_018937	Bacteria;Bacteroidetes;Flavobacteriia;Flavobacteriales;Flavobacteriaceae;
OTU99_025034	Bacteria;Bacteroidetes;Flavobacteriia;Flavobacteriales;Flavobacteriaceae;Croceibacter;
OTU99_006248	Bacteria;Bacteroidetes;Flavobacteriia;Flavobacteriales;Flavobacteriaceae;Eudoraea;
OTU99_005062	Bacteria;Bacteroidetes;Flavobacteriia;Flavobacteriales;Flavobacteriaceae;Flavobacterium;
OTU99_015746	Bacteria;Bacteroidetes;Flavobacteriia;Flavobacteriales;Flavobacteriaceae;Flavobacterium;
OTU99_009084	Bacteria;Bacteroidetes;Flavobacteriia;Flavobacteriales;Flavobacteriaceae;Flavobacterium;
OTU99_013107	Bacteria;Bacteroidetes;Flavobacteriia;Flavobacteriales;Flavobacteriaceae;Flavobacterium;
OTU99_002339	Bacteria;Bacteroidetes;Flavobacteriia;Flavobacteriales;Flavobacteriaceae;Flavobacterium;
OTU99_013066	Bacteria;Bacteroidetes;Flavobacteriia;Flavobacteriales;Flavobacteriaceae;Flavobacterium;
OTU99_001529	Bacteria;Bacteroidetes;Flavobacteriia;Flavobacteriales;Flavobacteriaceae;Flavobacterium;
OTU99_005036	Bacteria;Bacteroidetes;Flavobacteriia;Flavobacteriales;Flavobacteriaceae;Flavobacterium;
OTU99_009382	Bacteria;Bacteroidetes;Flavobacteriia;Flavobacteriales;Flavobacteriaceae;Flavobacterium;
OTU99_000364	Bacteria;Bacteroidetes;Flavobacteriia;Flavobacteriales;Flavobacteriaceae;Flavobacterium;
OTU99_001765	Bacteria;Bacteroidetes;Flavobacteriia;Flavobacteriales;Flavobacteriaceae;Flavobacterium;
OTU99_000108	Bacteria;Bacteroidetes;Flavobacteriia;Flavobacteriales;Flavobacteriaceae;Flavobacterium;
OTU99_000149	Bacteria;Bacteroidetes;Flavobacteriia;Flavobacteriales;Flavobacteriaceae;Flavobacterium;
OTU99_003408	Bacteria;Bacteroidetes;Flavobacteriia;Flavobacteriales;Flavobacteriaceae;Flavobacterium;
OTU99_010564	Bacteria;Bacteroidetes;Flavobacteriia;Flavobacteriales;Flavobacteriaceae;Flavobacterium;
OTU99_029822	Bacteria;Bacteroidetes;Flavobacteriia;Flavobacteriales;Flavobacteriaceae;Flavobacterium;
OTU99_005695	Bacteria;Bacteroidetes;Flavobacteriia;Flavobacteriales;Flavobacteriaceae;Flavobacterium;
OTU99_004723	Bacteria;Bacteroidetes;Flavobacteriia;Flavobacteriales;Flavobacteriaceae;Flavobacterium;
OTU99_000772	Bacteria;Bacteroidetes;Flavobacteriia;Flavobacteriales;Flavobacteriaceae;Flavobacterium;
OTU99_000017	Bacteria;Bacteroidetes;Flavobacteriia;Flavobacteriales;Flavobacteriaceae;Flavobacterium;
OTU99_000896	Bacteria;Bacteroidetes;Flavobacteriia;Flavobacteriales;Flavobacteriaceae;Flavobacterium;
OTU99_001434	Bacteria;Bacteroidetes;Flavobacteriia;Flavobacteriales;Flavobacteriaceae;Flavobacterium;
OTU99_026993	Bacteria;Bacteroidetes;Flavobacteriia;Flavobacteriales;Flavobacteriaceae;Flavobacterium;
OTU99_025474	Bacteria;Bacteroidetes;Flavobacteriia;Flavobacteriales;Flavobacteriaceae;Flavobacterium;
OTU99_000600	Bacteria;Bacteroidetes;Flavobacteriia;Flavobacteriales;Flavobacteriaceae;Flavobacterium;
OTU99_000248	Bacteria;Bacteroidetes;Flavobacteriia;Flavobacteriales;Flavobacteriaceae;Flavobacterium;
OTU99_000295	Bacteria;Bacteroidetes;Flavobacteriia;Flavobacteriales;Flavobacteriaceae;Flavobacterium;
OTU99_008487	Bacteria;Bacteroidetes;Flavobacteriia;Flavobacteriales;Flavobacteriaceae;Flavobacterium;
OTU99_003446	Bacteria;Bacteroidetes;Flavobacteriia;Flavobacteriales;Flavobacteriaceae;Flavobacterium;
OTU99_001322	Bacteria;Bacteroidetes;Flavobacteriia;Flavobacteriales;Flavobacteriaceae;Flavobacterium;
OTU99_030314	Bacteria;Bacteroidetes;Flavobacteriia;Flavobacteriales;Flavobacteriaceae;Flavobacterium;
OTU99_000024	Bacteria;Bacteroidetes;Flavobacteriia;Flavobacteriales;Flavobacteriaceae;Flavobacterium;
OTU99_015965	Bacteria;Bacteroidetes;Flavobacteriia;Flavobacteriales;Flavobacteriaceae;Flavobacterium;
OTU99_033421	Bacteria;Bacteroidetes;Flavobacteriia;Flavobacteriales;Flavobacteriaceae;Flavobacterium;
OTU99_001368	Bacteria;Bacteroidetes;Flavobacteriia;Flavobacteriales;Flavobacteriaceae;Flavobacterium;
OTU99_007556	Bacteria;Bacteroidetes;Flavobacteriia;Flavobacteriales;Flavobacteriaceae;Flavobacterium;
OTU99_013756	Bacteria;Bacteroidetes;Flavobacteriia;Flavobacteriales;Flavobacteriaceae;Flavobacterium;
OTU99_026593	Bacteria;Bacteroidetes;Flavobacteriia;Flavobacteriales;Flavobacteriaceae;Flavobacterium;
OTU99_000339	Bacteria;Bacteroidetes;Flavobacteriia;Flavobacteriales;Flavobacteriaceae;Flavobacterium;
OTU99_003959	Bacteria;Bacteroidetes;Flavobacteriia;Flavobacteriales;Flavobacteriaceae;Flavobacterium;
OTU99_026676	Bacteria;Bacteroidetes;Flavobacteriia;Flavobacteriales;Flavobacteriaceae;Flavobacterium;
OTU99_002129	Bacteria;Bacteroidetes;Flavobacteriia;Flavobacteriales;Flavobacteriaceae;Flavobacterium;
OTU99_003763	Bacteria;Bacteroidetes;Flavobacteriia;Flavobacteriales;Flavobacteriaceae;Flavobacterium;

OTU	Rank taxonomy
OTU99_001613	Bacteria;Bacteroidetes;Flavobacteriia;Flavobacteriales;Flavobacteriaceae;Flavobacterium;
OTU99_017033	Bacteria;Bacteroidetes;Flavobacteriia;Flavobacteriales;Flavobacteriaceae;Flavobacterium;
OTU99_005153	Bacteria;Bacteroidetes;Flavobacteriia;Flavobacteriales;Flavobacteriaceae;Flavobacterium;
OTU99_005606	Bacteria;Bacteroidetes;Flavobacteriia;Flavobacteriales;Flavobacteriaceae;Flavobacterium;
OTU99_004376	Bacteria;Bacteroidetes;Flavobacteriia;Flavobacteriales;Flavobacteriaceae;Flavobacterium;
OTU99_001063	Bacteria;Bacteroidetes;Flavobacteriia;Flavobacteriales;Flavobacteriaceae;Flavobacterium;
OTU99_004652	Bacteria;Bacteroidetes;Flavobacteriia;Flavobacteriales;Flavobacteriaceae;Flavobacterium;
OTU99_011827	Bacteria;Bacteroidetes;Flavobacteriia;Flavobacteriales;Flavobacteriaceae;Flavobacterium;
OTU99_006612	Bacteria;Bacteroidetes;Flavobacteriia;Flavobacteriales;Flavobacteriaceae;Flavobacterium;
OTU99_000901	Bacteria;Bacteroidetes;Flavobacteriia;Flavobacteriales;Flavobacteriaceae;Flavobacterium;
OTU99_000620	Bacteria;Bacteroidetes;Flavobacteriia;Flavobacteriales;Flavobacteriaceae;Flavobacterium;
OTU99_006972	Bacteria;Bacteroidetes;Flavobacteriia;Flavobacteriales;Flavobacteriaceae;Flavobacterium;
OTU99_007861	Bacteria;Bacteroidetes;Flavobacteriia;Flavobacteriales;Flavobacteriaceae;Flavobacterium;
OTU99_014928	Bacteria;Bacteroidetes;Flavobacteriia;Flavobacteriales;Flavobacteriaceae;Flavobacterium;
OTU99_009077	Bacteria;Bacteroidetes;Flavobacteriia;Flavobacteriales;Flavobacteriaceae;Flavobacterium;
OTU99_006170	Bacteria;Bacteroidetes;Flavobacteriia;Flavobacteriales;Flavobacteriaceae;Flavobacterium;
OTU99_000493	Bacteria;Bacteroidetes;Flavobacteriia;Flavobacteriales;Flavobacteriaceae;Flavobacterium;
OTU99_009481	Bacteria;Bacteroidetes;Flavobacteriia;Flavobacteriales;Flavobacteriaceae;Flavobacterium;
OTU99_001696	Bacteria;Bacteroidetes;Flavobacteriia;Flavobacteriales;Flavobacteriaceae;Flavobacterium;
OTU99_002284	Bacteria;Bacteroidetes;Flavobacteriia;Flavobacteriales;Flavobacteriaceae;Flavobacterium;
OTU99_002608	Bacteria;Bacteroidetes;Flavobacteriia;Flavobacteriales;Flavobacteriaceae;Flavobacterium;
OTU99_000908	Bacteria;Bacteroidetes;Flavobacteriia;Flavobacteriales;Flavobacteriaceae;Flavobacterium;
OTU99_031259	Bacteria;Bacteroidetes;Flavobacteriia;Flavobacteriales;Flavobacteriaceae;Flavobacterium;
OTU99_004905	Bacteria;Bacteroidetes;Flavobacteriia;Flavobacteriales;Flavobacteriaceae;Flavobacterium;
OTU99_000777	Bacteria;Bacteroidetes;Flavobacteriia;Flavobacteriales;Flavobacteriaceae;Flavobacterium;
OTU99_025910	Bacteria;Bacteroidetes;Flavobacteriia;Flavobacteriales;Flavobacteriaceae;Formosa;
OTU99_000754	Bacteria;Bacteroidetes;Flavobacteriia;Flavobacteriales;Flavobacteriaceae;Formosa;
OTU99_025929	Bacteria;Bacteroidetes;Flavobacteriia;Flavobacteriales;Flavobacteriaceae;Lutibacter;
OTU99_009659	Bacteria;Bacteroidetes;Flavobacteriia;Flavobacteriales;Flavobacteriaceae;Muricauda;
OTU99_001835	Bacteria;Bacteroidetes;Flavobacteriia;Flavobacteriales;Flavobacteriaceae;NS2b
OTU99_008491	Bacteria;Bacteroidetes;Flavobacteriia;Flavobacteriales;Flavobacteriaceae;NS2b
OTU99_024007	Bacteria;Bacteroidetes;Flavobacteriia;Flavobacteriales;Flavobacteriaceae;NS3a
OTU99_028437	Bacteria;Bacteroidetes;Flavobacteriia;Flavobacteriales;Flavobacteriaceae;NS3a
OTU99_030835	Bacteria;Bacteroidetes;Flavobacteriia;Flavobacteriales;Flavobacteriaceae;NS3a
OTU99_000676	Bacteria;Bacteroidetes;Flavobacteriia;Flavobacteriales;Flavobacteriaceae;NS3a
OTU99_029161	Bacteria;Bacteroidetes;Flavobacteriia;Flavobacteriales;Flavobacteriaceae;NS3a
OTU99_013816	Bacteria;Bacteroidetes;Flavobacteriia;Flavobacteriales;Flavobacteriaceae;NS3a
OTU99_007724	Bacteria;Bacteroidetes;Flavobacteriia;Flavobacteriales;Flavobacteriaceae;NS3a
OTU99_000012	Bacteria;Bacteroidetes;Flavobacteriia;Flavobacteriales;Flavobacteriaceae;NS3a
OTU99_006926	Bacteria;Bacteroidetes;Flavobacteriia;Flavobacteriales;Flavobacteriaceae;NS3a
OTU99_001238	Bacteria;Bacteroidetes;Flavobacteriia;Flavobacteriales;Flavobacteriaceae;NS3a
OTU99_003345	Bacteria;Bacteroidetes;Flavobacteriia;Flavobacteriales;Flavobacteriaceae;NS3a
OTU99_003982	Bacteria;Bacteroidetes;Flavobacteriia;Flavobacteriales;Flavobacteriaceae;NS3a
OTU99_019650	Bacteria;Bacteroidetes;Flavobacteriia;Flavobacteriales;Flavobacteriaceae;NS3a
OTU99_030074	Bacteria;Bacteroidetes;Flavobacteriia;Flavobacteriales;Flavobacteriaceae;NS3a
OTU99_006594	Bacteria;Bacteroidetes;Flavobacteriia;Flavobacteriales;Flavobacteriaceae;NS3a
OTU99_017587	Bacteria;Bacteroidetes;Flavobacteriia;Flavobacteriales;Flavobacteriaceae;NS3a
OTU99_005820	Bacteria;Bacteroidetes;Flavobacteriia;Flavobacteriales;Flavobacteriaceae;NS4
OTU99_020216	Bacteria;Bacteroidetes;Flavobacteriia;Flavobacteriales;Flavobacteriaceae;NS4
OTU99_030486	Bacteria;Bacteroidetes;Flavobacteriia;Flavobacteriales;Flavobacteriaceae;NS4
OTU99_000206	Bacteria;Bacteroidetes;Flavobacteriia;Flavobacteriales;Flavobacteriaceae;NS4
OTU99_023595	Bacteria;Bacteroidetes;Flavobacteriia;Flavobacteriales;Flavobacteriaceae;NS4
OTU99_009928	Bacteria;Bacteroidetes;Flavobacteriia;Flavobacteriales;Flavobacteriaceae;NS4
OTU99_013439	Bacteria;Bacteroidetes;Flavobacteriia;Flavobacteriales;Flavobacteriaceae;NS4
OTU99_002447	Bacteria;Bacteroidetes;Flavobacteriia;Flavobacteriales;Flavobacteriaceae;NS4
OTU99_008078	Bacteria;Bacteroidetes;Flavobacteriia;Flavobacteriales;Flavobacteriaceae;NS4
OTU99_020387	Bacteria;Bacteroidetes;Flavobacteriia;Flavobacteriales;Flavobacteriaceae;NS4
OTU99_026912	Bacteria;Bacteroidetes;Flavobacteriia;Flavobacteriales;Flavobacteriaceae;NS4
OTU99_006821	Bacteria;Bacteroidetes;Flavobacteriia;Flavobacteriales;Flavobacteriaceae;NS4
OTU99_023799	Bacteria;Bacteroidetes;Flavobacteriia;Flavobacteriales;Flavobacteriaceae;NS4
OTU99_025060	Bacteria;Bacteroidetes;Flavobacteriia;Flavobacteriales;Flavobacteriaceae;NS5
OTU99_007050	Bacteria;Bacteroidetes;Flavobacteriia;Flavobacteriales;Flavobacteriaceae;NS5
OTU99_019768	Bacteria;Bacteroidetes;Flavobacteriia;Flavobacteriales;Flavobacteriaceae;NS5
OTU99_000119	Bacteria;Bacteroidetes;Flavobacteriia;Flavobacteriales;Flavobacteriaceae;NS5
OTU99_025685	Bacteria;Bacteroidetes;Flavobacteriia;Flavobacteriales;Flavobacteriaceae;NS5
OTU99_018504	Bacteria;Bacteroidetes;Flavobacteriia;Flavobacteriales;Flavobacteriaceae;NS5
OTU99_024755	Bacteria;Bacteroidetes;Flavobacteriia;Flavobacteriales;Flavobacteriaceae;NS5
OTU99_006838	Bacteria;Bacteroidetes;Flavobacteriia;Flavobacteriales;Flavobacteriaceae;NS5
OTU99_017849	Bacteria;Bacteroidetes;Flavobacteriia;Flavobacteriales;Flavobacteriaceae;NS5
OTU99_013403	Bacteria;Bacteroidetes;Flavobacteriia;Flavobacteriales;Flavobacteriaceae;NS5
OTU99_030803	Bacteria;Bacteroidetes;Flavobacteriia;Flavobacteriales;Flavobacteriaceae;NS5
OTU99_010608	Bacteria;Bacteroidetes;Flavobacteriia;Flavobacteriales;Flavobacteriaceae;NS5
OTU99_006436	Bacteria;Bacteroidetes;Flavobacteriia;Flavobacteriales;Flavobacteriaceae;NS5
OTU99_015551	Bacteria;Bacteroidetes;Flavobacteriia;Flavobacteriales;Flavobacteriaceae;NS5
OTU99_005724	Bacteria;Bacteroidetes;Flavobacteriia;Flavobacteriales;Flavobacteriaceae;NS5
OTU99_025774	Bacteria;Bacteroidetes;Flavobacteriia;Flavobacteriales;Flavobacteriaceae;NS5
OTU99_000272	Bacteria;Bacteroidetes;Flavobacteriia;Flavobacteriales;Flavobacteriaceae;NS5
OTU99_022262	Bacteria;Bacteroidetes;Flavobacteriia;Flavobacteriales;Flavobacteriaceae;NS5
OTU99_019770	Bacteria;Bacteroidetes;Flavobacteriia;Flavobacteriales;Flavobacteriaceae;NS5
OTU99_024948	Bacteria;Bacteroidetes;Flavobacteriia;Flavobacteriales;Flavobacteriaceae;NS5
OTU99_006312	Bacteria;Bacteroidetes;Flavobacteriia;Flavobacteriales;Flavobacteriaceae;NS5
OTU99_006861	Bacteria;Bacteroidetes;Flavobacteriia;Flavobacteriales;Flavobacteriaceae;NS5
OTU99_019731	Bacteria;Bacteroidetes;Flavobacteriia;Flavobacteriales;Flavobacteriaceae;NS5

OTU	Rank taxonomy
OTU99_000082	Bacteria;Bacteroidetes;Flavobacteriia;Flavobacteriales;Flavobacteriaceae;NS5
OTU99_009013	Bacteria;Bacteroidetes;Flavobacteriia;Flavobacteriales;Flavobacteriaceae;NS5
OTU99_004257	Bacteria;Bacteroidetes;Flavobacteriia;Flavobacteriales;Flavobacteriaceae;NS5
OTU99_014255	Bacteria;Bacteroidetes;Flavobacteriia;Flavobacteriales;Flavobacteriaceae;NS5
OTU99_007094	Bacteria;Bacteroidetes;Flavobacteriia;Flavobacteriales;Flavobacteriaceae;NS5
OTU99_025035	Bacteria;Bacteroidetes;Flavobacteriia;Flavobacteriales;Flavobacteriaceae;NS5
OTU99_002213	Bacteria;Bacteroidetes;Flavobacteriia;Flavobacteriales;Flavobacteriaceae;NS5
OTU99_006740	Bacteria;Bacteroidetes;Flavobacteriia;Flavobacteriales;Flavobacteriaceae;NS5
OTU99_004631	Bacteria;Bacteroidetes;Flavobacteriia;Flavobacteriales;Flavobacteriaceae;NS5
OTU99_023787	Bacteria;Bacteroidetes;Flavobacteriia;Flavobacteriales;Flavobacteriaceae;NS5
OTU99_009523	Bacteria;Bacteroidetes;Flavobacteriia;Flavobacteriales;Flavobacteriaceae;NS5
OTU99_010327	Bacteria;Bacteroidetes;Flavobacteriia;Flavobacteriales;Flavobacteriaceae;NS5
OTU99_002063	Bacteria;Bacteroidetes;Flavobacteriia;Flavobacteriales;Flavobacteriaceae;NS5
OTU99_005883	Bacteria;Bacteroidetes;Flavobacteriia;Flavobacteriales;Flavobacteriaceae;NS5
OTU99_000643	Bacteria;Bacteroidetes;Flavobacteriia;Flavobacteriales;Flavobacteriaceae;NS5
OTU99_003029	Bacteria;Bacteroidetes;Flavobacteriia;Flavobacteriales;Flavobacteriaceae;NS5
OTU99_000447	Bacteria;Bacteroidetes;Flavobacteriia;Flavobacteriales;Flavobacteriaceae;NS5
OTU99_009776	Bacteria;Bacteroidetes;Flavobacteriia;Flavobacteriales;Flavobacteriaceae;NS5
OTU99_006753	Bacteria;Bacteroidetes;Flavobacteriia;Flavobacteriales;Flavobacteriaceae;NS5
OTU99_001144	Bacteria;Bacteroidetes;Flavobacteriia;Flavobacteriales;Flavobacteriaceae;NS5
OTU99_000693	Bacteria;Bacteroidetes;Flavobacteriia;Flavobacteriales;Flavobacteriaceae;NS5
OTU99_018651	Bacteria;Bacteroidetes;Flavobacteriia;Flavobacteriales;Flavobacteriaceae;Polaribacter;
OTU99_013956	Bacteria;Bacteroidetes;Flavobacteriia;Flavobacteriales;Flavobacteriaceae;Polaribacter;
OTU99_000137	Bacteria;Bacteroidetes;Flavobacteriia;Flavobacteriales;Flavobacteriaceae;Polaribacter;
OTU99_008275	Bacteria;Bacteroidetes;Flavobacteriia;Flavobacteriales;Flavobacteriaceae;Polaribacter;
OTU99_024343	Bacteria;Bacteroidetes;Flavobacteriia;Flavobacteriales;Flavobacteriaceae;Polaribacter;
OTU99_028556	Bacteria;Bacteroidetes;Flavobacteriia;Flavobacteriales;Flavobacteriaceae;Polaribacter;
OTU99_029895	Bacteria;Bacteroidetes;Flavobacteriia;Flavobacteriales;Flavobacteriaceae;Polaribacter;
OTU99_007008	Bacteria;Bacteroidetes;Flavobacteriia;Flavobacteriales;Flavobacteriaceae;Polaribacter;
OTU99_025808	Bacteria;Bacteroidetes;Flavobacteriia;Flavobacteriales;Flavobacteriaceae;Polaribacter;
OTU99_002022	Bacteria;Bacteroidetes;Flavobacteriia;Flavobacteriales;Flavobacteriaceae;Polaribacter;
OTU99_010567	Bacteria;Bacteroidetes;Flavobacteriia;Flavobacteriales;Flavobacteriaceae;Robiginitalea;
OTU99_004603	Bacteria;Bacteroidetes;Flavobacteriia;Flavobacteriales;Flavobacteriaceae;Robiginitalea;
OTU99_017178	Bacteria;Bacteroidetes;Flavobacteriia;Flavobacteriales;Flavobacteriaceae;uncultured;
OTU99_025168	Bacteria;Bacteroidetes;Flavobacteriia;Flavobacteriales;Flavobacteriaceae;uncultured;
OTU99_028824	Bacteria;Bacteroidetes;Flavobacteriia;Flavobacteriales;Flavobacteriaceae;uncultured;
OTU99_030007	Bacteria;Bacteroidetes;Flavobacteriia;Flavobacteriales;Flavobacteriaceae;uncultured;
OTU99_014975	Bacteria;Bacteroidetes;Flavobacteriia;Flavobacteriales;Flavobacteriaceae;uncultured;
OTU99_000403	Bacteria;Bacteroidetes;Flavobacteriia;Flavobacteriales;Flavobacteriaceae;uncultured;
OTU99_006244	Bacteria;Bacteroidetes;Flavobacteriia;Flavobacteriales;Flavobacteriaceae;uncultured;
OTU99_026306	Bacteria;Bacteroidetes;Flavobacteriia;Flavobacteriales;Flavobacteriaceae;Winogradskyella;
OTU99_027023	Bacteria;Bacteroidetes;Flavobacteriia;Flavobacteriales;NS9
OTU99_032789	Bacteria;Bacteroidetes;Flavobacteriia;Flavobacteriales;NS9
OTU99_009172	Bacteria;Bacteroidetes;Flavobacteriia;Flavobacteriales;NS9
OTU99_003369	Bacteria;Bacteroidetes;Flavobacteriia;Flavobacteriales;NS9
OTU99_001512	Bacteria;Bacteroidetes;Sphingobacteriia;Sphingobacteriales;NS11-12
OTU99_005145	Bacteria;Bacteroidetes;Sphingobacteriia;Sphingobacteriales;NS11-12
OTU99_008286	Bacteria;Bacteroidetes;Sphingobacteriia;Sphingobacteriales;NS11-12
OTU99_000054	Bacteria;Bacteroidetes;Sphingobacteriia;Sphingobacteriales;NS11-12
OTU99_003022	Bacteria;Bacteroidetes;Sphingobacteriia;Sphingobacteriales;NS11-12
OTU99_008821	Bacteria;Bacteroidetes;Sphingobacteriia;Sphingobacteriales;NS11-12
OTU99_000214	Bacteria;Bacteroidetes;Sphingobacteriia;Sphingobacteriales;NS11-12
OTU99_005125	Bacteria;Bacteroidetes;Sphingobacteriia;Sphingobacteriales;NS11-12
OTU99_020819	Bacteria;Bacteroidetes;Sphingobacteriia;Sphingobacteriales;NS11-12
OTU99_009461	Bacteria;Bacteroidetes;Sphingobacteriia;Sphingobacteriales;NS11-12
OTU99_007069	Bacteria;Bacteroidetes;Sphingobacteriia;Sphingobacteriales;NS11-12
OTU99_025226	Bacteria;Bacteroidetes;Sphingobacteriia;Sphingobacteriales;NS11-12
OTU99_014864	Bacteria;Bacteroidetes;Sphingobacteriia;Sphingobacteriales;Sphingobacteriaceae;
OTU99_002375	Bacteria;Bacteroidetes;Sphingobacteriia;Sphingobacteriales;Sphingobacteriaceae;Pedobacter;
OTU99_003749	Bacteria;Candidate
OTU99_004078	Bacteria;Cyanobacteria;Melainabacteria;Vampirovibrionales;
OTU99_000799	Bacteria;Planctomycetes;Phycisphaerae;Phycisphaerales;Phycisphaeraceae;CL500-3;
OTU99_001806	Bacteria;Planctomycetes;Phycisphaerae;Phycisphaerales;Phycisphaeraceae;CL500-3;
OTU99_001883	Bacteria;Planctomycetes;Phycisphaerae;Phycisphaerales;Phycisphaeraceae;CL500-3;
OTU99_009367	Bacteria;Planctomycetes;Phycisphaerae;Phycisphaerales;Phycisphaeraceae;CL500-3;
OTU99_003412	Bacteria;Planctomycetes;Phycisphaerae;Phycisphaerales;Phycisphaeraceae;CL500-3;
OTU99_018811	Bacteria;Proteobacteria;
OTU99_032081	Bacteria;Proteobacteria;Alphaproteobacteria;Rhodobacterales;Rhodobacteraceae;
OTU99_000304	Bacteria;Proteobacteria;Alphaproteobacteria;Rhodobacterales;Rhodobacteraceae;
OTU99_002707	Bacteria;Proteobacteria;Alphaproteobacteria;Rhodobacterales;Rhodobacteraceae;Candidatus
OTU99_025793	Bacteria;Proteobacteria;Alphaproteobacteria;Rhodobacterales;Rhodobacteraceae;Candidatus
OTU99_006807	Bacteria;Proteobacteria;Alphaproteobacteria;Rickettsiales;mitochondria;
OTU99_004381	Bacteria;Proteobacteria;Alphaproteobacteria;Rickettsiales;mitochondria;
OTU99_007520	Bacteria;Proteobacteria;Alphaproteobacteria;Rickettsiales;mitochondria;
OTU99_014677	Bacteria;Proteobacteria;Alphaproteobacteria;SAR11
OTU99_002541	Bacteria;Proteobacteria;Alphaproteobacteria;SAR11
OTU99_000018	Bacteria;Proteobacteria;Alphaproteobacteria;SAR11
OTU99_001386	Bacteria;Proteobacteria;Alphaproteobacteria;SAR11
OTU99_014787	Bacteria;Proteobacteria;Alphaproteobacteria;SAR11
OTU99_008234	Bacteria;Proteobacteria;Alphaproteobacteria;SAR11
OTU99_020659	Bacteria;Proteobacteria;Alphaproteobacteria;SAR11
OTU99_022816	Bacteria;Proteobacteria;Alphaproteobacteria;SAR11
OTU99_001116	Bacteria;Proteobacteria;Alphaproteobacteria;SAR11

OTU	Rank taxonomy
OTU99_013789	Bacteria;Proteobacteria;Alphaproteobacteria;SAR11
OTU99_017422	Bacteria;Proteobacteria;Alphaproteobacteria;SAR11
OTU99_013432	Bacteria;Proteobacteria;Alphaproteobacteria;SAR11
OTU99_000165	Bacteria;Proteobacteria;Alphaproteobacteria;SAR11
OTU99_008397	Bacteria;Proteobacteria;Alphaproteobacteria;SAR11
OTU99_013079	Bacteria;Proteobacteria;Alphaproteobacteria;SAR11
OTU99_006165	Bacteria;Proteobacteria;Alphaproteobacteria;SAR11
OTU99_026623	Bacteria;Proteobacteria;Alphaproteobacteria;SAR11
OTU99_000200	Bacteria;Proteobacteria;Alphaproteobacteria;SAR11
OTU99_001852	Bacteria;Proteobacteria;Alphaproteobacteria;SAR11
OTU99_006291	Bacteria;Proteobacteria;Alphaproteobacteria;SAR11
OTU99_025745	Bacteria;Proteobacteria;Alphaproteobacteria;SAR11
OTU99_032648	Bacteria;Proteobacteria;Alphaproteobacteria;SAR11
OTU99_007812	Bacteria;Proteobacteria;Alphaproteobacteria;SAR11
OTU99_030081	Bacteria;Proteobacteria;Alphaproteobacteria;SAR11
OTU99_014103	Bacteria;Proteobacteria;Alphaproteobacteria;SAR11
OTU99_004819	Bacteria;Proteobacteria;Alphaproteobacteria;SAR11
OTU99_024850	Bacteria;Proteobacteria;Alphaproteobacteria;SAR11
OTU99_006644	Bacteria;Proteobacteria;Alphaproteobacteria;SAR11
OTU99_013054	Bacteria;Proteobacteria;Alphaproteobacteria;SAR11
OTU99_002056	Bacteria;Proteobacteria;Alphaproteobacteria;SAR11
OTU99_009608	Bacteria;Proteobacteria;Alphaproteobacteria;SAR11
OTU99_007343	Bacteria;Proteobacteria;Alphaproteobacteria;SAR11
OTU99_013654	Bacteria;Proteobacteria;Alphaproteobacteria;SAR11
OTU99_007449	Bacteria;Proteobacteria;Alphaproteobacteria;SAR11
OTU99_001395	Bacteria;Proteobacteria;Alphaproteobacteria;SAR11
OTU99_013292	Bacteria;Proteobacteria;Alphaproteobacteria;SAR11
OTU99_015536	Bacteria;Proteobacteria;Alphaproteobacteria;SAR11
OTU99_015291	Bacteria;Proteobacteria;Alphaproteobacteria;SAR11
OTU99_015509	Bacteria;Proteobacteria;Alphaproteobacteria;SAR11
OTU99_008490	Bacteria;Proteobacteria;Alphaproteobacteria;SAR11
OTU99_014485	Bacteria;Proteobacteria;Alphaproteobacteria;SAR11
OTU99_001873	Bacteria;Proteobacteria;Alphaproteobacteria;SAR11
OTU99_008446	Bacteria;Proteobacteria;Alphaproteobacteria;SAR11
OTU99_003335	Bacteria;Proteobacteria;Alphaproteobacteria;SAR11
OTU99_005275	Bacteria;Proteobacteria;Alphaproteobacteria;SAR11
OTU99_008059	Bacteria;Proteobacteria;Alphaproteobacteria;SAR11
OTU99_002644	Bacteria;Proteobacteria;Alphaproteobacteria;SAR11
OTU99_013242	Bacteria;Proteobacteria;Alphaproteobacteria;SAR11
OTU99_001752	Bacteria;Proteobacteria;Alphaproteobacteria;SAR11
OTU99_014150	Bacteria;Proteobacteria;Betaproteobacteria;Burkholderiales;Comamonadaceae;Limnohabitans;
OTU99_001659	Bacteria;Proteobacteria;Betaproteobacteria;Burkholderiales;Comamonadaceae;Limnohabitans;
OTU99_016947	Bacteria;Proteobacteria;Betaproteobacteria;Burkholderiales;Comamonadaceae;Limnohabitans;
OTU99_001306	Bacteria;Proteobacteria;Betaproteobacteria;Burkholderiales;Comamonadaceae;Polaromonas;
OTU99_007063	Bacteria;Proteobacteria;Gammaproteobacteria;
OTU99_002583	Bacteria;Proteobacteria;Gammaproteobacteria;
OTU99_014960	Bacteria;Proteobacteria;Gammaproteobacteria;
OTU99_009345	Bacteria;Proteobacteria;Gammaproteobacteria;
OTU99_008570	Bacteria;Proteobacteria;Gammaproteobacteria;
OTU99_007248	Bacteria;Proteobacteria;Gammaproteobacteria;
OTU99_019797	Bacteria;Proteobacteria;Gammaproteobacteria;
OTU99_002278	Bacteria;Proteobacteria;Gammaproteobacteria;
OTU99_004210	Bacteria;Proteobacteria;Gammaproteobacteria;
OTU99_004461	Bacteria;Proteobacteria;Gammaproteobacteria;
OTU99_001114	Bacteria;Proteobacteria;Gammaproteobacteria;
OTU99_003842	Bacteria;Proteobacteria;Gammaproteobacteria;
OTU99_024681	Bacteria;Proteobacteria;Gammaproteobacteria;Alteromonadales;
OTU99_006657	Bacteria;Proteobacteria;Gammaproteobacteria;Alteromonadales;Alteromonadaceae;
OTU99_015816	Bacteria;Proteobacteria;Gammaproteobacteria;Alteromonadales;Alteromonadaceae;Alteromonas;
OTU99_000584	Bacteria;Proteobacteria;Gammaproteobacteria;Alteromonadales;Alteromonadaceae;Alteromonas;
OTU99_009489	Bacteria;Proteobacteria;Gammaproteobacteria;Alteromonadales;Alteromonadaceae;Glaciecola;
OTU99_002292	Bacteria;Proteobacteria;Gammaproteobacteria;Alteromonadales;Alteromonadaceae;Glaciecola;
OTU99_010595	Bacteria;Proteobacteria;Gammaproteobacteria;Alteromonadales;Alteromonadaceae;Glaciecola;
OTU99_004535	Bacteria;Proteobacteria;Gammaproteobacteria;Alteromonadales;Colwelliaceae;Colwellia;
OTU99_000636	Bacteria;Proteobacteria;Gammaproteobacteria;Alteromonadales;Colwelliaceae;Colwellia;
OTU99_013358	Bacteria;Proteobacteria;Gammaproteobacteria;Alteromonadales;Colwelliaceae;Colwellia;
OTU99_004181	Bacteria;Proteobacteria;Gammaproteobacteria;Alteromonadales;Colwelliaceae;Colwellia;
OTU99_010600	Bacteria;Proteobacteria;Gammaproteobacteria;Alteromonadales;Idiomarinaceae;Idiomarina;
OTU99_025204	Bacteria;Proteobacteria;Gammaproteobacteria;Alteromonadales;Pseudoalteromonadaceae;Pseudoalteromonas;
OTU99_013100	Bacteria;Proteobacteria;Gammaproteobacteria;Alteromonadales;Psychromonadaceae;Psychromonas;
OTU99_025655	Bacteria;Proteobacteria;Gammaproteobacteria;Alteromonadales;Psychromonadaceae;Psychromonas;
OTU99_003314	Bacteria;Proteobacteria;Gammaproteobacteria;Alteromonadales;Psychromonadaceae;Psychromonas;
OTU99_006743	Bacteria;Proteobacteria;Gammaproteobacteria;Alteromonadales;Shewanellaceae;Shewanella;
OTU99_023894	Bacteria;Proteobacteria;Gammaproteobacteria;Alteromonadales;Shewanellaceae;Shewanella;
OTU99_016036	Bacteria;Proteobacteria;Gammaproteobacteria;Alteromonadales;Shewanellaceae;Shewanella;
OTU99_002415	Bacteria;Proteobacteria;Gammaproteobacteria;Alteromonadales;Shewanellaceae;Shewanella;
OTU99_003034	Bacteria;Proteobacteria;Gammaproteobacteria;Alteromonadales;Shewanellaceae;Shewanella;
OTU99_008419	Bacteria;Proteobacteria;Gammaproteobacteria;Chromatiales;Chromatiaceae;Rheinheimera;
OTU99_004197	Bacteria;Proteobacteria;Gammaproteobacteria;Chromatiales;Chromatiaceae;Rheinheimera;
OTU99_031272	Bacteria;Proteobacteria;Gammaproteobacteria;Chromatiales;Chromatiaceae;Rheinheimera;
OTU99_023852	Bacteria;Proteobacteria;Gammaproteobacteria;Chromatiales;Chromatiaceae;Rheinheimera;
OTU99_004600	Bacteria;Proteobacteria;Gammaproteobacteria;Chromatiales;Chromatiaceae;Rheinheimera;
OTU99_026309	Bacteria;Proteobacteria;Gammaproteobacteria;Chromatiales;Chromatiaceae;Rheinheimera;

OTU	Rank taxonomy
OTU99_002450	Bacteria;Proteobacteria;Gammaproteobacteria;Chromatiales;Chromatiaceae;Rheinheimera;
OTU99_025972	Bacteria;Proteobacteria;Gammaproteobacteria;Oceanospirillales;
OTU99_019353	Bacteria;Proteobacteria;Gammaproteobacteria;Oceanospirillales;
OTU99_002743	Bacteria;Proteobacteria;Gammaproteobacteria;Oceanospirillales;
OTU99_013239	Bacteria;Proteobacteria;Gammaproteobacteria;Oceanospirillales;
OTU99_013254	Bacteria;Proteobacteria;Gammaproteobacteria;Oceanospirillales;
OTU99_001654	Bacteria;Proteobacteria;Gammaproteobacteria;Oceanospirillales;
OTU99_005502	Bacteria;Proteobacteria;Gammaproteobacteria;Oceanospirillales;Oceanospirillaceae;Pseudospirillum;
OTU99_013056	Bacteria;Proteobacteria;Gammaproteobacteria;Oceanospirillales;Oceanospirillaceae;Pseudospirillum;
OTU99_002863	Bacteria;Proteobacteria;Gammaproteobacteria;Oceanospirillales;OM182
OTU99_006438	Bacteria;Proteobacteria;Gammaproteobacteria;Oceanospirillales;OM182
OTU99_001250	Bacteria;Proteobacteria;Gammaproteobacteria;Oceanospirillales;OM182
OTU99_002009	Bacteria;Proteobacteria;Gammaproteobacteria;Oceanospirillales;SAR86
OTU99_024849	Bacteria;Proteobacteria;Gammaproteobacteria;Oceanospirillales;SAR86
OTU99_008939	Bacteria;Proteobacteria;Gammaproteobacteria;Pseudomonadales;Moraxellaceae;Psychrobacter;
OTU99_027533	Bacteria;Proteobacteria;Gammaproteobacteria;Pseudomonadales;Pseudomonadaceae;Pseudomonas;
OTU99_003501	Bacteria;Proteobacteria;Gammaproteobacteria;Vibrionales;Vibrionaceae;
OTU99_010004	Bacteria;Proteobacteria;Gammaproteobacteria;Vibrionales;Vibrionaceae;Aliivibrio;
OTU99_025423	Bacteria;Proteobacteria;Gammaproteobacteria;Vibrionales;Vibrionaceae;Vibrio;
OTU99_005792	Bacteria;Proteobacteria;Gammaproteobacteria;Vibrionales;Vibrionaceae;Vibrio;
OTU99_000801	Unclassified;
OTU99_021625	Unclassified;
OTU99_003202	Unclassified;
OTU99_006218	Unclassified;
OTU99_006274	Unclassified;
OTU99_004961	Unclassified;
OTU99_003809	Unclassified;
OTU99_008246	Unclassified;
OTU99_015591	Unclassified;
OTU99_025841	Unclassified;
OTU99_024734	Unclassified;
OTU99_027677	Unclassified;
OTU99_003091	Unclassified;
OTU99_017136	Unclassified;
OTU99_005630	Unclassified;
